# General continuous-time Markov model of sequence evolution via insertions/deletions: local alignment probability computation

**DOI:** 10.1186/s12859-016-1167-6

**Published:** 2016-09-27

**Authors:** Kiyoshi Ezawa

**Affiliations:** 1 Department of Bioscience and Bioinformatics, Kyushu Institute of Technology, Iizuka, 820-8502 Japan; 2 Department of Biology and Biochemistry, University of Houston, Houston, TX 77204-5001 USA

**Keywords:** Stochastic evolutionary model, Insertion/deletion (indel), Sequence alignment probability, Indel likelihood, Power-law length distribution, Evolutionary simulation, Perturbation theory, Practically exact solution

## Abstract

**Background:**

Insertions and deletions (indels) account for more nucleotide differences between two related DNA sequences than substitutions do, and thus it is imperative to develop a method to reliably calculate the occurrence probabilities of sequence alignments via evolutionary processes on an entire sequence. Previously, we presented a perturbative formulation that facilitates the *ab initio* calculation of alignment probabilities under a continuous-time Markov model, which describes the stochastic evolution of an *entire* sequence via indels with quite general rate parameters. And we demonstrated that, under some conditions, the *ab initio* probability of an alignment can be factorized into the product of an overall factor and contributions from regions (or local alignments) delimited by gapless columns.

**Results:**

Here, using our formulation, we attempt to approximately calculate the probabilities of local alignments under space-homogeneous cases. First, for each of all types of local pairwise alignments (PWAs) and some typical types of local multiple sequence alignments (MSAs), we numerically computed the total contribution from all parsimonious indel histories and that from all next-parsimonious histories, and compared them. Second, for some common types of local PWAs, we derived two integral equation systems that can be numerically solved to give practically exact solutions. We compared the total parsimonious contribution with the practically exact solution for each such local PWA. Third, we developed an algorithm that calculates the first-approximate MSA probability by multiplying total parsimonious contributions from all local MSAs. Then we compared the first-approximate probability of each local MSA with its absolute frequency in the MSAs created via a genuine sequence evolution simulator, Dawg. In all these analyses, the total parsimonious contributions approximated the multiplication factors fairly well, as long as gap sizes and branch lengths are at most moderate. Examination of the accuracy of another indel probabilistic model in the light of our formulation indicated some modifications necessary for the model’s accuracy improvement.

**Conclusions:**

At least under moderate conditions, the approximate methods can quite accurately calculate *ab initio* alignment probabilities under biologically more realistic models than before. Thus, our formulation will provide other indel probabilistic models with a sound reference point.

**Electronic supplementary material:**

The online version of this article (doi:10.1186/s12859-016-1167-6) contains supplementary material, which is available to authorized users.

## Background

The evolution of DNA, RNA, and protein sequences is driven by mutations such as base substitutions, insertions and deletions (indels), recombination, and other genomic rearrangements (e.g., [1–3]). Thus far, analyses on substitutions have predominated in the field of molecular evolutionary study, in particular using the probabilistic (or likelihood) theory of substitutions that is now widely accepted (e.g., [4–6]). This is probably because evolutionary models describing residue substitutions are relatively easier to handle. However, it must be remembered that the study of indels is at least as crucial as the study of substitutions. There are two major reasons for this. First, it is indels but not substitutions that yield the *skeletons* (or the gap configurations) of the sequence alignments (reviewed, e.g., in [7]), which provide essential inputs to most homology-based analyses in computational biology. And second, some recent comparative genomic analyses have revealed that indels account for more base differences between the genomes of closely related species than substitutions (e.g., [8–12]). These circumstances make it imperative to develop a stochastic model that enables us to reliably calculate the probability of sequence evolution via mutations including insertions and deletions. Since the groundbreaking works by Bishop and Thompson [13] and by Thorne, Kishino and Felsenstein [14], many studies have been done to develop and apply methods to calculate the probabilities of pairwise alignments (PWAs) and multiple sequence alignments (MSAs) under the probabilistic models aiming to incorporate the effects of indels. Such methods have greatly improved in terms of the computational efficiency and the scope of application. See excellent reviews for details on this topic (e.g., [15–17]). A majority of these studies are based on hidden Markov models (HMMs) (e.g., [18]) or transducer theories (e.g., [19]). Both of them calculate the indel component of an alignment probability as a product of inter-column transition probabilities or of block-wise contributions. And the study on these methods is still advancing, strengthening their mathematical and algorithmic bases (e.g., [20, 21]). Unfortunately, most of these methods have at least either of two fundamental problems, one regarding the evolutionary consistency and the other regarding the biological realism. (For details, see Background of [22].) Regarding the evolutionary consistency, it is a priori unclear whether or how a HMM or a transducer is related with any *genuine* stochastic evolutionary model (or “evolutionary model” for short), which describes the evolution of an *entire* sequence via indels along a time axis. Regarding the biological realism, the standard HMMs or transducers can at best handle mixed geometric distributions of indel lengths (e.g., [23]) (and usually implement simple geometric distributions), whereas many empirical studies showed that the real indel lengths are distributed according to power-laws (e.g., [24] and references therein). Besides, very few studies thus far (e.g., [25]) addressed the issue of indel rate variation across regions.

In a previous study [22], we presented a theoretical formulation that facilitates the *ab initio* calculation of alignment probabilities under a *genuine* stochastic evolutionary model, specifically, a general continuous-time Markov model of sequence evolution via indels. Our evolutionary model was created as a result of incorporating the idea of position-specific evolutionary rates [25] into the most general “substitution/insertion/deletion model” [26]. Thus, the model is naturally devoid of the aforementioned two problems. Aided by some techniques of time-dependent perturbation theory in quantum mechanics [27–29], we formally expanded the *ab initio* probability of an alignment into a series of terms with different numbers of indels. This expansion gave an intuitively clearer representation of Feller’s theorems [30]. And it theoretically underpinned the stochastic evolutionary simulation method of Gillespie [31], which provides the foundation for genuine sequence evolution simulators (e.g., [32–34]). And we also showed that, if the indel model parameters satisfy a certain set of conditions, the *ab initio* probability of an alignment is indeed factorable into the product of an overall factor and contributions from local alignments delimited by preserved ancestral sites (PASs), i.e., gapless columns. This suggested that the evolutionary models satisfying such conditions could provide a sort of generalized HMMs, which extend the space- and time-homogeneous “long indel” model [26] to some space- and time-heterogeneous situations.

In this paper, we focus on how to concretely calculate the contribution from each local alignment, assuming that the indel model satisfies the conditions for factorable alignment probabilities. (To clearly illustrate the concrete computations, we deal with space-homogeneous models in the bulk of this manuscript (i.e., in sections R2-R6), and briefly discuss extensions to more general cases near the end (i.e., in subsection R7.1).) As noted in [26] and section R1 of Results and discussion of this manuscript, the contribution from each local alignment is a summation over an infinite number of local indel histories. Thus it cannot be computed *literally* exactly within a finite amount of time. This makes it necessary to devise some approximation methods, each of which sums contributions from a finite number of indel histories (as first proposed in [26]). An auspice is that indel rates (say, *λ*_*ID*_ indels per site per unit time) are known to be at most around 1/10 of the substitution rates (say, *λ*_*S*_ substitutions per site per unit time) (e.g., [24, 35]). And the probability of an indel history involving *N*_*ID*_ indels is roughly $O\left({\left({\lambda}_{ID}\;t\right)}^{N_{ID}}\right)$ times the probability of a history with no indel, where *t* is a time scale characteristic of the system under consideration. In conjunction, these suggest that taking account only of histories with minimum and near-minimum required numbers of indels may provide a good approximation to each local alignment probability, as long as the sequence divergences (e.g., *λ*_*S*_ *t*) are within the scope of phylogenetic analyses (i.e., at most *O*(1) substitutions per site).

In section R1 of Results and discussion, we briefly review the relevant portion of the theoretical basis that was established in our previous study [22]. We introduce simplified notation so that we can focus on a single local alignment. In sections R2-R4, we demonstrate how our perturbative formulation can be concretely used to approximately calculate the contributions to the *ab initio* alignment probabilities from local alignments, i.e., alignment regions separated by gapless columns. We examine all types of local pairwise alignments (PWAs) in section R2, and some typical types of local multiple sequence alignments (MSAs) in section R4. For each local alignment type, we calculate the total parsimonious contribution and the total next-parsimonious contribution to its probability (more precisely, its multiplication factor). In section R3, we discuss two systems of integral equations that can be numerically solved to give practically exact solutions (or “exact” solutions, for short) for some common types of local PWAs. There, we also study the behaviors of the “exact” solutions. Then, by comparing the total parsimonious contribution with the total next-parsimonious contribution, or with the “exact” solution, we investigate the parameter regions in which the total parsimonious contribution can approximate the alignment probability quite accurately (in sections R2-R4). In section R5, we perform simulation analyses with a genuine evolutionary simulator, Dawg [32], to examine whether or not the conclusions from sections R2-R4 also apply to local MSAs of more general types. For this purpose, we developed an algorithm to calculate the “first-approximate” probability of a given MSA under a given parameter setting (including a given tree) by multiplying the overall factor and the total parsimonious contributions from all local MSAs. And we examine the accuracy of the first-approximate multiplication factors calculated by the algorithm. In section R6, we use our *ab initio* formulation as a “yardstick” to measure the accuracy of other indel probabilistic models. As a representative model, we chose the generalized HMM of [36], which aims for the biological realism but not fully for the evolutionary consistency. In section R7, we discuss some outstanding issues and possible improvements, extensions and applications of the presented algorithm and methods. The topics include the risks associated with the naïve application of our algorithm or methods to *reconstructed* alignments. The sections in Methods describe the settings for numerical analyses (M1) and simulation analyses (M2). And the sections in Supplementary methods in Additional file 1 explain methodological details on concrete perturbation calculations and the first-approximate algorithm.

This paper basically uses the same conventions and notations as used in [22]. Briefly, a sequence state *s* (∈ *S*^*II*^) is represented as an array of sites, each of which is equipped with an ancestry index (*υ*_*x*_ ∈ *ϒ*).^1^ (In this study, we focus on indels. Hence, we do not consider the residue states of sequences. For the incorporation of residue states and substitutions, see, e.g., [37, 38].) And each indel event is represented as an operator acting on the bra-vector, 〈*s*|, representing a sequence state. More specifically, the operator ${\widehat{M}}_I\left(x,\kern0.5em l\right)$ denotes the insertion of *l* sites between the *x*-th and (*x* + 1) th sites, and the operator ${\widehat{M}}_D\left({x}_B,\kern0.5em {x}_E\right)$ denotes the deletion of a sub-array between (and including) the *x*_*B*_-th and the *x*_*E*_-th sites. Readers unfamiliar with the bra-ket notation (as adapted from theoretical physics (e.g., [27, 28])) can simply regard a bra-vector (〈*s*|), a ket-vector (|*s*^′^〉) and an operator $\left(\widehat{M}\right)$ as convenient reminders of a row vector, a column vector and a matrix, respectively, just as in the standard representation of a continuous-time Markov model. (See section SA-1 in Additional file 2 of [22] for the equivalence between them.) And, also as in [22], the following terminology is used. The term “an indel process” means a series of successive indel events with both the order and the timing specified. And the term “an indel history” means a series of successive indel events with only the order specified.

As a last note, an “alignment,” a “PWA” and a “MSA” in this paper will mean their homology structures [39]. Briefly, the homology structure of an alignment is a set of alignment columns (i.e., sets of homologous sites in the aligned sequences) that are spatially arranged in a looser way than in a usual alignment, i.e., constrained only by the spatial relationships between the sites within each aligned sequence.^2^

## Results and discussion

[Descriptions given here are somewhat sketchy. For methodological details, as well as the relationship with the results of [22], see the relevant sections of Supplementary methods in Additional file 1.]

### R1. Perturbation expansion of multiplication factor for local alignment

In this section, we briefly explain some results in [22] that are essential for this paper. Similarly to that of the probabilistic alignment methods in general, one of the main goals of our theoretical formulation (presented in [22]) is to calculate the absolute occurrence probabilities of the alignments and to compare the calculated alignment probabilities. Therefore, unless stated otherwise, the probabilities considered in this paper are *not* conditioned on a particular alignment (or even on extant sequences). (Once the absolute probabilities are calculated, such conditional probabilities (e.g., of indel histories) could be obtained by dividing the absolute probabilities of the outcomes (e.g., the indel histories) by the absolute probability of the condition (e.g., the resulting alignment), similarly to Eq. (SM-5.3.6a) in Additional file 1.)

Let *P*[(*α*(*s*^*A*^, *s*^*D*^), [*t*_*I*_, *t*_*F*_]) | (*s*^*A*^, *t*_*I*_)] be the probability that a PWA (*α*(*s*^*A*^, *s*^*D*^)) between an ancestral sequence state (*s*^*A*^) and a descendant (*s*^*D*^) result from the evolution of a sequence during a time interval ([*t*_*I*_, *t*_*F*_]), given *s*^*A*^ at *t*_*I*_. In [22], we formally showed that *P*[(*α*(*s*^*A*^, *s*^*D*^), [*t*_*I*_, *t*_*F*_]) | (*s*^*A*^, *t*_*I*_)] is given as a series:

1 $$ P\left[\left(\alpha \left({s}^A,{s}^D\right),\kern0.5em \left[{t}_I,\kern0.5em {t}_F\right]\right)\kern0.5em \left|\kern0.5em \left({s}^A,\kern0.5em {t}_I\right)\right.\right]={\displaystyle {\sum}_{N={N}_{min}\left[\alpha \left({s}^A,{s}^D\right)\right]}^{\infty }{P}_{(N)}\left[\left(\alpha \left({s}^A,{s}^D\right),\kern0.5em \left[{t}_I,\kern0.5em {t}_F\right]\right)\kern0.5em \left|\kern0.5em \left({s}^A,\kern0.5em {t}_I\right)\right.\right]} $$

Here, *N*_*min*_[*α*(*s*^*A*^, *s*^*D*^)] is the minimum number of indels required to create *α*(*s*^*A*^, *s*^*D*^). And *P*_(*N*)_[(*α*(*s*^*A*^, *s*^*D*^), [*t*_*I*_, *t*_*F*_]) | (*s*^*A*^, *t*_*I*_)] is the fraction of *P*[(*α*(*s*^*A*^, *s*^*D*^), [*t*_*I*_, *t*_*F*_]) | (*s*^*A*^, *t*_*I*_)] contributed from all *N*-event indel histories that can yield *α*(*s*^*A*^, *s*^*D*^). A “preserved ancestral site” (PAS) is a site of *s*^*A*^ that was hit by no indel and thus was preserved all through [*t*_*I*_, *t*_*F*_]. Now, using some (but not necessarily all) PASs, we partition *α*(*s*^*A*^, *s*^*D*^) into “local regions” (i.e., inter-PAS regions), ${\gamma}_1,\kern0.5em {\gamma}_2,\kern0.5em \dots, \kern0.5em {\gamma}_{\kappa_{max}}$, in which all potentially causative indels are confined. In [22], we derived the two conditions.

- **Condition (i):** Each indel rate parameter is independent of the portion of the sequence state outside of the local region where the indel occurred.
- **Condition (ii)**: The increment of the exit rate due to each indel event is independent of the portion of the sequence state outside of the local region where the indel occurred. (The “exit rate” of a state is the rate at which the system “exits” the state, that is, the total rate at which the state changes to any of other states.)

Under these conditions, the PWA probability, Eq. (1), can be factorized as:

2 $$ \begin{array}{l}\kern1em P\left[\left(\alpha \left({s}^A,{s}^D\right),\kern0.5em \left[{t}_I,\kern0.5em {t}_F\right]\right)\kern0.5em \left|\kern0.5em \left({s}^A,\kern0.5em {t}_I\right)\right.\right]\kern0.5em \\ {}=\kern0.5em P\left[\left(\left[,\kern.2em \right],\kern0.5em \left[{t}_I,\;{t}_F\right]\right)\kern0.5em \left|\kern0.5em \left({s}^A,\kern0.5em {t}_I\right)\right.\right]\kern0.5em {\displaystyle \prod_{\kappa =1}^{\kappa_{max}}{\tilde{\mu}}_P\left[{\gamma}_{\kappa };\kern0.5em \left(\alpha \left({s}^A,\kern0.5em {s}^D\right),\kern0.5em \left[{t}_I,{t}_F\right]\right)\kern0.5em \left|\kern0.5em \left({s}^A,\kern0.5em {t}_I\right)\right.\right]}\end{array} $$

Here *P*[([ ], [*t*_*I*_, *t*_*F*_]) | (*s*^*A*^, *t*_*I*_)] is the probability that the sequence underwent no indels during [*t*_*I*_, *t*_*F*_], given *s*^*A*^at *t*_*I*_. And ${\tilde{\mu}}_P\left[{\gamma}_{\kappa };\kern0.5em \left(\alpha \left({s}^A,\kern0.5em {s}^D\right),\kern0.5em \left[{t}_I,{t}_F\right]\right)\kern0.5em \left|\kern0.5em \left({s}^A,\kern0.5em {t}_I\right)\right.\right]$ is the multiplication factor contributed from the local region, *γ*_*κ*_. Because the multiplication factor is a summation of contributions over all local indel histories that can yield the local PWA confined in *γ*_*κ*_ , it can also be expressed as a series similar to Eq. (1):

3 $$ {\tilde{\mu}}_P\left[{\gamma}_{\kappa };\kern0.5em \left(\alpha \left({s}^A,\kern0.5em {s}^D\right),\kern0.5em \left[{t}_I,{t}_F\right]\right)\kern0.5em \left|\kern0.5em \left({s}^A,\kern0.5em {t}_I\right)\right.\right]\kern0.5em =\kern0.5em {\displaystyle {\sum}_{N={N}_{min}\left[\alpha \left({s}^A,\kern0.5em {s}^D\right);\kern0.5em {\gamma}_{\kappa}\right]}^{\infty }{\mu}_{P\kern0.5em (N)}\left[{\gamma}_{\kappa };\kern0.5em \left(\alpha \left({s}^A,\kern0.5em {s}^D\right),\kern0.5em \left[{t}_I,{t}_F\right]\right)\kern0.5em \left|\kern0.5em \left({s}^A,\kern0.5em {t}_I\right)\right.\right]} $$

Here, *N*_*min*_[*α*(*s*^*A*^, *s*^*D*^); *γ*_*κ*_] is the minimum number of indels required for the portion of *α*(*s*^*A*^, *s*^*D*^) in *γ*_*κ*_. And the term *μ*_*P* (*N*)_[*γ*_*κ*_; (*α*(*s*^*A*^, *s*^*D*^), [*t*_*I*_, *t*_*F*_]) | (*s*^*A*^, *t*_*I*_)] is the portion of the multiplication factor contributed from all local-PWA-consistent *N*-indel local histories in *γ*_*κ*_.^3^ (For more details, see the first half of SM-1 of Supplementary methods in Additional file 1.)

It should be noted that the multiplication factor, ${\tilde{\mu}}_P\left[\dots \right]$ (e.g., in Eq. (2)), is *not* a probability; actually, it is not even a conditional probability, and it can exceed 1 (unity) in some cases (in such manners that the entire right hand side of Eq. (2) will always be less than 1 (unity)). In this sense, the “generalized HMM” given by Eq. (2) differs from normal HMMs. The contribution of each local indel history to a multiplication factor is the ratio of the probability of the history (given an ancestral state) to the probability that the ancestral state underwent no indel. (See Eq. (SM-1.7) in Additional file 1 for the mathematical definition.) When comparing the contributions from two different sets of histories (potentially giving rise to the same local alignment), the denominator (i.e., the probability of no indel) is usually identical. Therefore, in general, the comparison of two multiplication factor contributions gives the same result as the comparison of the corresponding probabilities. (Similar notes apply also to the analyses of local MSAs below.)

Similar arguments hold also for the probability, $P\left[\alpha \left[{s}_1,\kern0.5em {s}_2,\dots, \kern0.5em {s}_{N^X}\right]\kern0.5em \left|\kern0.5em T\right.\right]$, that a MSA ($\alpha \left[{s}_1,\kern0.5em {s}_2,\dots, \kern0.5em {s}_{N^X}\right]$) of *N*^*X*^ sequences, ${s}_1,\kern0.5em {s}_2,\dots, \kern0.5em {s}_{N^X}$, results from the evolution along a given phylogenetic tree (*T*) [22]. Basically in line with the idea in [18, 19, 40], we can build up the probability of a MSA, first by multiplying the root state probability and the probabilities of ancestor–descendant PWAs along branches, and second by summing such products over all MSA-consistent ancestral states. The *ab initio* MSA probability thus composed can be expressed as a series:

4 $$ P\left[\alpha \left[{s}_1,\kern0.5em {s}_2,\dots, \kern0.5em {s}_{N^X}\right]\kern0.5em \left|\kern0.5em T\right.\right]={\displaystyle {\sum}_{N={N}_{min}}^{\infty }{P}_{(N)}\left[\alpha \left[{s}_1,\kern0.5em {s}_2,\dots, \kern0.5em {s}_{N^X}\right]\kern0.5em \left|\kern0.5em T\right.\right]} $$

Here, *N*_min_ is the minimum number of indels required for creating the MSA. (For simplicity, we omitted the obvious dependence of *N*_min_ on the MSA and the tree.) And ${P}_{(N)}\left[\alpha \left[{s}_1,\kern0.5em {s}_2,\dots, \kern0.5em {s}_{N^X}\right]\kern0.5em \left|\kern0.5em T\right.\right]$ is the portion of the probability contributed from all MSA-consistent *N*-event indel histories. A MSA-counterpart of a PAS is a gapless column, which indicates that the corresponding site was hit by no indel throughout the evolution along *T*. (Hereafter, a gapless column in a MSA is also called a “PAS.”) Using some PASs, we partition the MSA into local regions, ${C}_1,\kern0.5em {C}_2,\kern0.5em \dots, \kern0.5em {C}_{K_{max}}$. Meanwhile, there are infinitely many possible root sequence states (*s*^*Root*^ ' *s*) consistent with the MSA. Among them, we choose one as the “reference” root state (*s*_0_^*Root*^). Then, in addition to the aforementioned conditions (i) and (ii), we impose the following condition.

- **Condition (iii):** the (prior) probability of each root state (*s*^*Root*^) is given by the probability of *s*_0_^*Root*^ multiplied by the product of factors over the local regions, where each factor depends only on the difference between *s*^*Root*^ and *s*_0_^*Root*^ in a local region.

Under these conditions, the MSA probability is factorized as:

5 $$ P\left[\alpha \left[{s}_1,\kern0.5em {s}_2,\dots, \kern0.5em {s}_{N^X}\right]\kern0.5em \left|\kern0.5em T\right.\right]\kern0.5em =\kern0.5em {P}_0\left[{s}_0^{Root}\kern0.5em \left|\kern0.5em T\right.\right]\kern0.5em {\displaystyle \prod_{K=1}^{K_{max}}{\tilde{\overset{\smile }{M}}}_P\left[\alpha \left[{s}_1,\kern0.5em {s}_2,\dots, \kern0.5em {s}_{N^X}\right];\kern0.5em {s}_0^{Root};\kern0.5em {C}_K\kern0.5em \left|\kern0.5em T\right.\right]} $$

Here, *P*_0_[*s*_0_^*Root*^ | *T*] is the probability that the root sequence state is *s*_0_^*Root*^ and that it was hit by no indel all across *T*. And ${\tilde{\overset{\smile }{M}}}_P\left[\alpha \left[{s}_1,\kern0.5em {s}_2,\dots, \kern0.5em {s}_{N^X}\right];\kern0.5em {s}_0^{Root};\kern0.5em {C}_K\kern0.5em \left|\kern0.5em T\right.\right]$ is the multiplication factor contributed from the local region, *C*_K_. As in Eq. (4), the multiplication factor also can be expressed as a series:

6 $$ {\tilde{\overset{\smile }{M}}}_P\left[\alpha \left[{s}_1,\kern0.5em {s}_2,\dots, \kern0.5em {s}_{N^X}\right];\kern0.5em {s}_0^{Root};\kern0.5em {C}_K\kern0.5em \left|\kern0.5em T\right.\right]\kern0.5em =\kern0.5em {\displaystyle {\sum}_{N={N}_{min}\left[{C}_K\right]}^{\infty }{\overset{\smile }{M}}_{P\kern0.5em (N)}\left[\alpha \left[{s}_1,\kern0.5em {s}_2,\dots, \kern0.5em {s}_{N^X}\right];\kern0.5em {s}_0^{Root};\kern0.5em {C}_K\kern0.5em \left|\kern0.5em T\right.\right]} $$

Here, *N*_*min*_[*C*_*Κ*_] is the minimum number of indels required for the portion of the MSA in *C*_K_. And the term ${\overset{\smile }{M}}_{P\kern0.5em (N)}\left[\alpha \left[{s}_1,\kern0.5em {s}_2,\dots, \kern0.5em {s}_{N^X}\right];\kern0.5em {s}_0^{Root};\kern0.5em {C}_K\kern0.5em \left|\kern0.5em T\right.\right]$ is the fraction of the multiplication factor contributed from all local-MSA-consistent *N*-indel local histories in *C*_K_. (For more details, see the second half of SM-1 of Supplementary methods in Additional file 1.)

As agued above, under conditions (i) and (ii) (and, in addition, (iii) for a MSA), the probability of a given alignment is factorized into the product of an overall factor and local contributions (as in Eq. (2) and Eq. (5)). This factorization could drastically speed up the computation of the probability. However, each local contribution is still a summation over an infinite number of indel histories (Eq. (3) and Eq. (6)), and its *literally* exact calculation would take infinitely long. This study examines two kinds of approximation methods. One is the “first approximation,” which approximates each multiplication factor with the “total parsimonious contribution,” i.e., the summation of contributions over all possible parsimonious indel histories. (It corresponds to the term, *μ*_*P* (*N*)_[*γ*_*κ*_; (*α*(*s*^*A*^, *s*^*D*^), [*t*_*I*_, *t*_*F*_]) | (*s*^*A*^, *t*_*I*_)] with *N* = *N*_*min*_[*α*(*s*^*A*^, *s*^*D*^); *γ*_*κ*_] or ${\overset{\smile }{M}}_{P\kern0.5em (N)}\left[\alpha \left[{s}_1,\kern0.5em {s}_2,\dots, \kern0.5em {s}_{N^X}\right];\kern0.5em {s}_0^{Root};\kern0.5em {C}_K\kern0.5em \left|\kern0.5em T\right.\right]$ with *N* = *N*_*min*_[*C*_*Κ*_].) And the other is calculating a *practically* exact solution (or an “exact” solution, for short) of each local contribution from a local PWA of a certain type (see section R3). Especially, we examine the accuracy of the first approximation by comparing the total parsimonious contribution either with the total next-parsimonious contribution (sections R2 and R4), with the “exact solution” (section R3) or with the results of simulations (section R5). Here, the “total next-parsimonous contribution” is the summation of contributions over all next-parsimonious indel histories. (It usually corresponds to the above term with *N* = *N*_*min*_[*α*(*s*^*A*^, *s*^*D*^); *γ*_*κ*_] + 1 or with *N* = *N*_*min*_[*C*_*Κ*_] + 1).

**Fig. 1:**
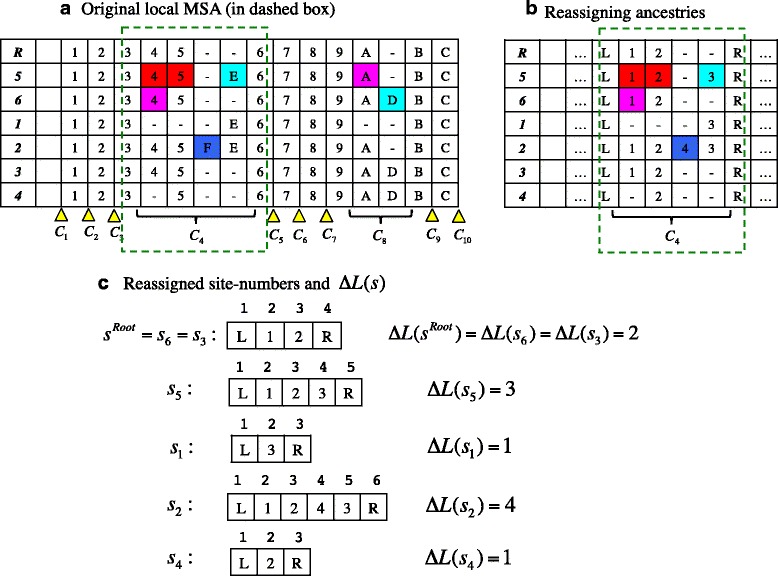
Notation and re-numbering of sites typical in this study. **a** An example MSA. The *C*_*κ*_’s (with *κ* = 1, 2, …, 10) label the regions that actually or potentially accommodate local indel histories (marked by bottom curly brackets and *yellow* wedges, respectively). As an illustration, we choose the local MSA confined in the region *C*_4_ (the portion in the *green* dashed box), and re-assign the ancestry indices (in the cells) as shown in panel (**b**). Ancestries *L* and *R* were newly assigned to the flanking PASs. The ancestry indices in between the PASs are just examples and not always assigned in this way. (The indices in panel (**a**) could be regarded as hexadecimal numerals, if preferred.) **c** Subsequences extracted from the local MSA. Shown above each site (i.e., each cell) is the site number (i.e., spatial coordinate) re-assigned to it. And the ∆*L*(*s*) on the right of each sequence (*s* = *s*_1_, …, *s*_6_ or *s* ^*Root*^) is the count of sites in between the PASs. In panels **a** and **b**, the boldface characters in the leftmost columns stand for the sequence IDs. (More precisely, the number ‘*i*’ stands for sequence *s*_*i*_, and the ‘*R*’ stands for the root sequence (*s* ^*Root*^).) A dash (‘-‘) in a cell represents a gap. In panel **b**, triple dots (‘…’) in a cell indicate that the alignment continues outwards. Panel **a** was adapted from Figure S2 b of [22]. Panels **b** and **c** were adapted from Figure 1 of [43]

In the following sections, we will work with a model that satisfies the conditions (i), (ii) and (iii), and we will focus on calculating the multiplication factor *that comes from a single local region* (i.e., a “local alignment”) flanked by a pair of PASs. As in [22], we will work in the state space *S*^*II*^. This means that we will calculate the probability of the *homology structure* of each local alignment (e.g., [39]). Let *ΔL*(*s*) be the number of sites that a sequence *s* ∈ *S*^*II*^ has between the pair of PASs. We will re-assign the site numbers so that the left- and right-flanking PASs are numbered 1 and *ΔL*(*s*) + 2, respectively, and the sites in between them are numbered 2, …, *ΔL*(*s*) + 1. This will make it easy to apply our theoretical formulation [22] to the current situation. We will re-assign the ancestries *υ*(1) = *L* and *υ*(*ΔL*(*s*) + 2) = *R* to the left- and right-flanking PASs, respectively. (See endnote **(1)** for a brief description of the ancestry.) And we will usually (but not always) re-assign the ancestries *υ*(*x*) = *x* − 1 to the sites in between the PASs, *x* = 2, …, *ΔL*(*s*) + 1, of the ancestral sequence, *s* = *s*^*A*^ (for a PWA), or the root sequence, *s* = *s*^*Root*^ (for a MSA). See Fig. 1 for an illustration.

Hereafter, we will often employ shorthand notations for the aforementioned (fractions of) multiplication factors, e.g., *μ*_*P* (*N*)_[*γ*_*κ*_; (*α*(*s*^*A*^, *s*^*D*^), [*t*_*I*_, *t*_*F*_]) | (*s*^*A*^, *t*_*I*_)] for a PWA and ${\overset{\smile }{M}}_{P(N)}\left[\alpha \left[{s}_1,\kern0.5em {s}_2,\dots, \kern0.5em {s}_{N^X}\right];\kern0.5em {s}_0^{Root};\kern0.5em {C}_K\kern0.5em \left|\kern0.5em T\right.\right]$ for a MSA, either by omitting the arguments (like “*μ*_*P* (*N*)_” and “${\overset{\smile }{M}}_{P\kern0.5em (N)}$”) or by replacing the arguments with simpler ones representing more concrete situations (like “*μ*_*P* (*N*)_[*case* (*ii*); *ΔL*^*A*^]” or “${\overset{\smile }{M}}_{P\kern0.5em (N)}\left[ case\kern0.5em (II);\kern0.5em \varDelta {L}^{D12}\right]$”). Unless stated otherwise, we consider the sequence evolution during time interval [*t*_*I*_, *t*_*F*_] (for a PWA) or along a given tree, *T* (for a MSA).

For illustration, we will use the indel evolutionary model of Dawg [32], though the analyses could be extended with due modifications to more general models (discussed in subsection R7.1).^4^ Its indel rates are space-homogeneous and time-homogeneous, and they are parametrized as follows. Let *L*(*s*) be the length of the sequence with state *s*. The rate of insertion ${\widehat{M}}_I\left(x,\kern0.5em l\right)$ is:

7 $$ {r}_I\left(x,l;s,t\right)\kern0.5em =\kern0.5em {\lambda}_I{f}_I(l)\kern1em \left(\mathrm{f}\mathrm{o}\mathrm{r}\kern0.5em x=0,1,\dots, L(s);\kern0.5em l=1,2,\dots {L}_I^{CO}\right) $$

Here, *λ*_*I*_ is the total insertion rate (per site), *f*_*I*_(*l*) is the insertion length distribution, and *L*_*I*_^*CO*^ is the cut-off insertion length. The rate of deletion ${\widehat{M}}_D\left({x}_B,\kern0.5em {x}_E\right)$ is:

8 $$ {r}_D\left({x}_B,{x}_E,;,, s,t\right)\kern0.5em =\kern0.5em {\lambda}_D{f}_D\left(l\kern0.5em =\kern0.5em {x}_E-{x}_B+1\right)\kern1em \left(\mathrm{f}\mathrm{o}\mathrm{r}\;{x}_B\le L(s);\kern0.5em {x}_E\ge 1;\kern0.5em l=1,2,\dots, {L}_D^{CO}\right) $$

Here, *λ*_*D*_ is the total deletion rate (per site), *f*_*D*_(*l*) is the deletion length distribution, and *L*_*D*_^*CO*^ is the cut-off deletion length. Consequently, the exit rate from state *s* is:

9 $$ {R}_X^{ID}\left(s,t\right)\kern0.5em \left(=\kern0.5em {\displaystyle {\sum}_{x=0}^{L(s)}{\displaystyle {\sum}_{l=1}^{L_I^{CO}}{r}_I\left(x,l;\kern0.5em s,t\right)}\kern0.5em +\kern0.5em {\displaystyle {\sum}_{x_B=-{L}_D^{CO}+2}^{L(s)}{\displaystyle {\sum}_{x_E= max\left\{{x}_B,\kern0.5em 1\right\}}^{x_B+{L}_D^{CO}-1}{r}_D\left({x}_B,\kern0.5em {x}_E;\kern0.5em s,t\right)}}}\right)=\kern0.5em \left({\lambda}_I\kern0.5em +\kern0.5em {\lambda}_D\right)L(s)+\kern0.5em {\varDelta}^{Dawg}\left[{\lambda}_I,{\lambda}_D,{f}_D(.)\right]. $$

Here, ${\varDelta}^{Dawg}\left[{\lambda}_I,{\lambda}_D,{f}_D(.)\right]\kern0.5em \equiv \kern0.5em {\lambda}_I\kern0.5em +\kern0.5em \left({\overline{l}}_D-1\right){\lambda}_D$ is a “universal” constant factor, and ${\overline{l}}_D\kern0.5em \equiv \kern0.5em {\displaystyle {\sum}_{l=1}^{L_D^{CO}}l\kern0.5em {g}_D\left(l,t\right)}$ is the average deletion length [32]. In this study, we use the power-law indel length distribution: ${f}_I(l)\kern0.5em =\kern0.5em {f}_D(l)\kern0.5em =\kern0.5em {l}^{-1.6}/\left[{\displaystyle {\sum}_{k=1}^{L_I^{CO}}{k}^{-1.6}}\right]$, which is among the typical ones empirically observed (e.g., [24] and references therein). We also set *λ*_*I*_ = *λ*_*D*_ according to a genome-wide data analysis [41], unless otherwise stated. As for the sequence state probabilities at the root, we assume a uniform sequence length distribution hereafter.^5^ See sections M1 and M2 of Methods for more specific settings.

### R2. Numerical comparison between parsimonious and next-parsimonious contributions (1): for local PWAs

Here, we examine how accurately the first approximation will estimate the multiplication factor from each local PWA by comparing the total parsimonious contribution with the total next-parsimonious contribution, both calculated via numerical computations of their analytical expressions (given in SM-2 of Supplementary methods in Additional file 1). In this study, we are concerned only with the homology structures [39] of alignments. Hence, local PWAs flanked by a pair of conserved ancestral sites (PASs) can be broadly classified into four cases, according to the sites between the PASs: (i) no ancestral or descendant sites (panel a of Additional file 1: Figure S1); (ii) some (*ΔL*^*A*^ > 0) ancestral sites but no descendant sites (panel b); (iii) some (*ΔL*^*D*^ > 0) descendant sites but no ancestral sites (panel c); and (iv) some (*ΔL*^*A*^ > 0) ancestral sites and some (*ΔL*^*D*^ > 0) descendant sites, but with no ancestor-descendant homology (panel d). 6 (See Additional file 1: Figure S2 for parsimonious and next-parsimonious indel histories in case (ii), and Figure S3 for parsimonious histories in case (iv).) Our numerical analyses indicated the following. In case (i), the total next-parsimonious contribution (*μ*_*P*(2)_[*case* (*i*)]) was negligibly smaller than the total parsimonious contribution (*μ*_*P*(0)_[*case* (*i*)] (=1)) for any realistic situation we likely encounter, as far as a single inter-PAS region is concerned. In case (ii) and case (iii), the total next-parsimonious contribution (*μ*_*P*(2)_[*case* (*ii*); *ΔL*^*A*^] or *μ*_*P*(2)_[*case* (*iii*); *ΔL*^*D*^]) amounted to 1/2 of the total parsimonious contribution (*μ*_*P*(1)_[*case* (*ii*); *ΔL*^*A*^] or *μ*_*P*(1)_[*case* (*iii*); *ΔL*^*D*^]), when the size of the local PWA (i.e., *ΔL*^*A*^ or *ΔL*^*D*^) is equal to a threshold value, (*ΔL*)_0.5_^(*NP*)^ ≈ 1.2/*E*[*n*_*ID*_] (Additional file 1: Table S1 and Figure S4). Here *E*[*n*_*ID*_] (=(*λ*_*I*_ + *λ*_*D*_)(*t*_*F*_ − *t*_*I*_)) is the expected number of indels per site during the sequence evolution. For example, in typical analyses of neutral genomic sequences from eutherian mammals, the branch length is around 0.2 expected substitutions per site (e.g., [42]). And the total indel rate was estimated as 1/8 of the total substitution rate [35]. Using these values, *E*[*n*_*ID*_] is approximately 0.2/8 = 0.025, which gives the threshold (*ΔL*)_0.5_^(*NP*)^ roughly equal to 50 sites. For the analyses of more closely related sequences, the threshold becomes longer. For example, in a comparison between primate sequences, a typical branch length would be 0.05 expected substitutions per site (e.g., [42]). Then, (*ΔL*)_0.5_^(*NP*)^ would be roughly equal to 200 sites. In case (iv), the total next-parsimonious contribution (*μ*_*P*(3)_[*case* (*iv*); *ΔL*^*A*^, *ΔL*^*D*^]) did not substantially exceed 1/2 of the total parsimonious contribution (*μ*_*P*(2)_[*case* (*iv*); *ΔL*^*A*^, *ΔL*^*D*^]) until the local PWA or the time interval became quite long (Table 1). For more details on these analyses, see SM-2 of Supplementary methods in Additional file 1. (Further details on the calculations for cases (iii) and (iv) are given in sections A1.1 and A1.2, respectively, in [43].)

**Table 1:** Perturbation analysis on local PWA probabilities in case (iv)

(∆*L* ^*A*^, ∆*L* ^*D*^)	0.01 indels/site	0.04 indels/site	0.1 indels/site	0.2 indels/site
(1, 1)	**0.003**	**0.010**	**0.024**	**0.045**
(3, 1)	**0.021**	**0.084**	**0.204**	**0.393**
(3, 3)	**0.042**	**0.166**	**0.402**	0.768
(5, 5)	**0.073**	**0.283**	0.672	1.256
(10, 1)	**0.064**	**0.246**	0.572	1.013
(10, 10)	**0.149**	0.561	1.292	2.288
(25, 1)	**0.151**	0.547	1.112	1.541
(25, 4)	**0.198**	0.723	1.519	2.234
(30, 10)	**0.288**	1.038	2.164	3.072
(100, 1)	0.537	1.333	1.507	1.574
(100, 3)	0.607	1.593	1.894	2.033
(300, 1)	1.165	1.394	1.427	1.527

Each cell shows the ratio of the total next-parsimonious contribution to the total parsimonious contribution, when there are ∆*L* ^*A*^ ancestral sites and ∆*L* ^*D*^ descendant sites in between the PASs. Each column is labeled with the expected number of indels per site ((*λ*_*I*_ + *λ*_*D*_)(*t*_*F*_ − *t*_*I*_)). See section M1  of Methods for the parameter setting. Because of the symmetry between probabilities under the time reversal, the ratio for (*ΔL* ^*A*^, *ΔL* ^*D*^) = (*L*_1_, *L*_2_) is identical to that for (*ΔL* ^*A*^, *ΔL* ^*D*^) = (*L*_2_, *L*_1_) when *λ*_*I*_ = *λ*_*D*_. Thus we only showed the results for *ΔL* ^*A*^ ≥ *ΔL* ^*D*^. The ratios that are less than 0.5 are shown in boldface. This table is identical to Table 2 of [43]

### R3. Numerical comparison between parsimonious contribution and "exact solution" for local PWAs

It is difficult to calculate the summed contributions from local histories involving more indels, especially in case (iv). We could exactly calculate the contribution from a single local history involving any number of indels if we use the algorithm for a “trajectory likelihood” given by Miklós et al. [26]. As we exemplified in Appendix A1.2 of [43], however, it is already quite hard to enumerate even all the possible next-parsimonious local indel histories for case (iv). Nevertheless, if we consider only cases (i), (ii), and (iii) under a (locally) space-homogeneous model, we can work out systems of exact integral equations that could in principle provide the numerical solutions for the total sum of contributions up to a desired level of accuracy, i.e., ${\tilde{\mu}}_P^{\left\langle {N}_{ID}\right\rangle}\equiv \kern0.5em {\displaystyle {\sum}_{N={N}_{min}}^{N_{ID}}{\mu}_{P(N)}}$ with a desired upper-bound indel count (*N*_*ID*_), at the expense of some time and memory.

**Fig. 2:**
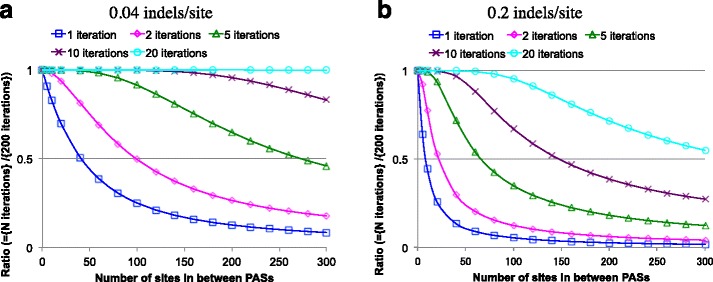
Iterative solutions of integral equation system for local PWA probabilities. The results shown in this figure apply to both case (ii) and case (iii) local PWAs. In both panels, the abscissa is the number of sites between PASs (i.e., ∆*L* ^*A*^ for case (ii) and ∆*L* ^*D*^ for case (iii)), with zero corresponding to case (i). Each panel shows the ratio of the total contribution from histories with up to (and including) *N*_*ID*_ indels to the “exact solution” of the multiplication factor. See section M1  of Methods for the parameter setting. Panel **a** is for 0.04 expected indels per site, and panel b is for 0.2 expected indels per site. Panels **a** and **b** in this figure are modified versions of panels B and C, respectively, of Figure 3 of [43]

Applying the fundamental defining integral equations of our evolutionary model (Eqs.(R4.4, R4.5) in [22]) to the local MSAs of cases (i), (ii) and (iii), two systems of integral equations can be derived. One system is for cases (i) and (ii) (see SM-3 of Supplementary methods in Additional file 1), and the other is for cases (i) and (iii) (described in Appendix A1.3 of [43]). 7 These systems of integral equations can be numerically solved by iteration, and the results after *N*_*ID*_ iterations give the aforementioned ${\tilde{\mu}}_P^{\left\langle {N}_{ID}\right\rangle }$ (see SM-3 of Supplementary methods). A naïve implementation of this iteration (based on Eq. (SM-3.2’) in Additional file 1) would be very slow, with the time complexity of *O*(*N*_*ID*_(*L*^*CO*^)^2^(*N*_*P*_)^2^). Here *L*^*CO*^ is the cut-off indel length and *N*_*P*_ is the number of equal-sized sub-time-intervals introduced for the numerical time integration. However, we devised a faster algorithm for this iteration (based on Eqs.(SM-3.4a,b) in Additional file 1), with the time complexity of *O*(*N*_*ID*_ *L*^*CO*^(*L*^*CO*^ + *N*_*P*_)*N*_*P*_). And we implemented it in Perl (available in Additional file 2). Typically, the computation finished in the order of an hour (using a single thread) in a Macintosh computer with two quad-core 2.26 GHz Intel Xeon processors and 8GB memory. One round of the computation provides the multiplication factors for all local PWA sizes (*ΔL* = 0, 1, …, *L*^*CO*^) and for time-interval sizes of *k* (*t*_*F*_ − *t*_*I*_)/*N*_*P*_ with *k* = 1, 2, …, *N*_*P*_. Our numerical analyses confirmed that *N*_*ID*_ = 200 would be enough to give the practically exact (or “exact”) solution for the local PWAs of 300 sites or less in likely situations of phylogenetic-level sequence analyses (data not shown). Thus, we used the results of *N*_*ID*_ = 200 iterations as the “exact” multiplication factors, and compared the parsimonious contributions with them (Fig. 2). We define another threshold value, (*ΔL*)_0.5_^(1)^, at which the parsimonious contribution becomes 1/2 of the “exact” solution. The results indicated (*ΔL*)_0.5_^(1)^ ≈ 1.6/*E*[*n*_*ID*_] (Additional file 1: Table S1). Thus, (*ΔL*)_0.5_^(1)^ is approximately 4/3 of (*ΔL*)_0.5_^(*NP*)^ in the previous section, implying that (*ΔL*)_0.5_^(*NP*)^ actually gives a somewhat conservative criterion for the goodness of the first approximation. A fringe benefit of these iteration analyses is that we can also assess the “*n*-th approximation,” which is given by ${\tilde{\mu}}_P^{\left\langle n\right\rangle }$ (Fig. 2). We define (*ΔL*)_0.5_^(*n*)^ as the local PWA size at which the *n*-th approximation becomes 1/2 of the “exact” solution. It seemed that (*ΔL*)_0.5_^(*n*)^ > *n* × (*ΔL*)_0.5_^(1)^ in general (Additional file 1: Table S1). This suggests the benefit of incorporating non-parsimonious local indel histories, especially when we deal with long local PWAs resulting from a long time evolution.

**Fig. 3:**
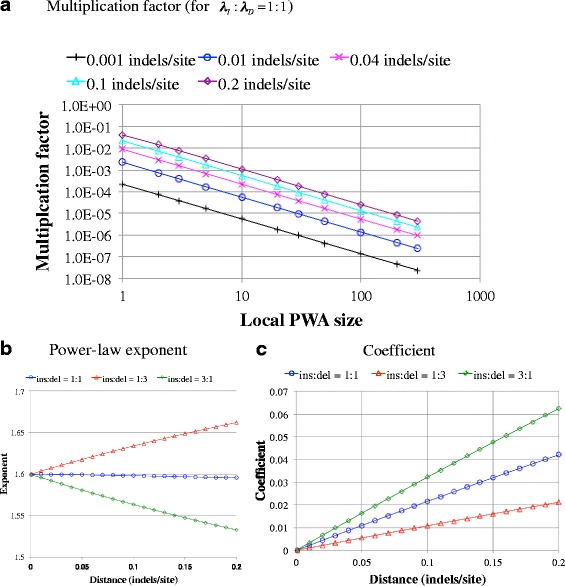
Power-law behaviors of “exact” multiplication factors from case (iii) local PWAs. **a** Log-log plots of the “exact” multiplication factors (${\mu}_P^{\left\langle {N}_{ID}=200\right\rangle}\left[\varDelta L\right]$, ordinate) against the local PWA size (∆*L*, abscissa), showing nearly perfect power-law behaviors. Although this panel shows the results under *λ*_*I*_ : *λ*_*D*_ = 1 : 1 only, the power-law approximation is actually very good also under *λ*_*I*_ : *λ*_*D*_ = 1 : 3 and *λ*_*I*_ : *λ*_*D*_ = 3 : 1 (Additional file 1: Table S2). Panels **b** and **c** show the power-law exponent (*γ*) and the coefficient (*A*), respectively, as functions of the distance ((*λ*_*I*_ + *λ*_*D*_)(*t* − *t*_*I*_) indels/site, abscissa) and the rate ratio (*λ*_*I*_ : *λ*_*D*_, different curves). Here, we assumed the approximate power-law relation, ${\mu}_P^{\left\langle {N}_{ID}=200\right\rangle}\left[\varDelta L\right]\kern0.5em \approx \kern0.5em A{\left(\varDelta L\right)}^{-\gamma }$. (See Additional file 1: Table S2 also for the results of correlation and regression analyses.) Note that the results apply also to case (ii) local PWAs with due modifications

Now that we have “exact” probabilities for local PWAs of cases (i), (ii), and (iii), it would be interesting to examine their behaviors. Panel a of Fig. 3 shows the log-log plots of exact solutions for different time intervals (in units of the expected number of indels per site). We see that even finite-time transition probabilities are well approximated by the power-law, with very high correlation coefficients for the log-log plots (0.9998 or more in the absolute value, see Additional file 1: Table S2). And panel b of Fig. 3 indicates that, as the time interval increases, the power-law exponent deviates gradually (yet only slightly**)** from its value for the instantaneous indel rates (1.6 here). Meanwhile, the coefficient seems almost proportional to the time interval (panel c). The slopes for these quantities differed for different values of the ratio, *λ*_*I*_ : *λ*_*D*_. (Additional file 1: Table S2 gives also their numerical values for some representative cases.) These results may be useful for future data analyses on indels, e.g., when inferring the power-law exponents for the indel rate parameters from the comparison of relatively divergent homologous sequences.

Similarly to our *ab initio* formulation itself, these systems of integral equations can accommodate any practical indel length distributions. Therefore, we could even examine cases where insertions and deletions follow different length distributions and/or models that incorporate transposon insertions (e.g., [44, 45]) as well. Such analyses should be interesting and important.

### R4. Numerical comparison between parsimonious and next-parsimonious contributions (2): for local MSAs

**Fig. 4:**
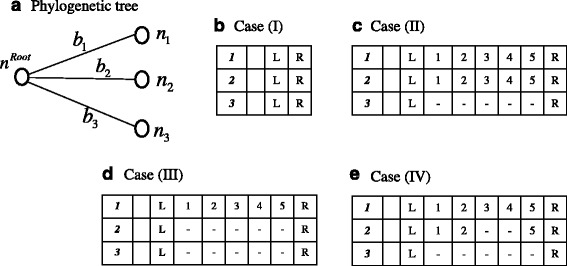
Gap configurations of local MSAs examined in perturbation analyses. **a** The 3-OTU tree used in the perturbation analyses. A node (open circle) is labeled *n*_*i*_ (*i* = 1,2 or 3) (external) or *n* ^*Root*^ (root). A branch is labeled *b*_*i*_ (*i* = 1,2 or 3). In the following, ∆*L* ^*Di*^ denotes the number of sites that the sequence at node *n*_*i*_ has between the PASs. **b** Local gap configuration in case (I). **c** Case (II) with *ΔL* ^*D*1^ = *ΔL* ^*D*2^ = 5. **d** Case (III) with *ΔL* ^*D*1^ = 5. **e** Case (IV) with *ΔL* ^*D*1^ = 5 and *ΔL* ^*D*2^ = 3. The figure is a modified version of Figure 4 of [43]

**Fig. 5:**
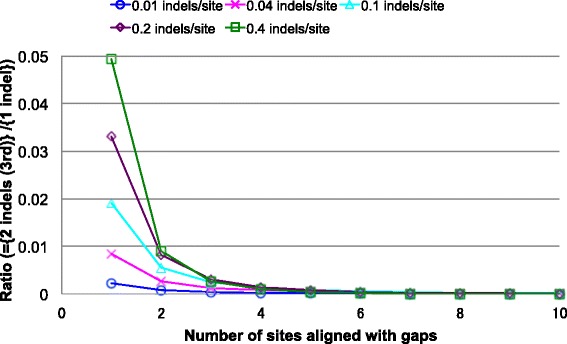
Perturbation analysis on local MSA probabilities in case (III). The graph shows the ratio of the contribution of the type (B) next-parsimonious history to that of the parsimonious history. The former history consists of a deletion along each of branches 2 and 3, and the latter consists of an insertion along branch 1. The ratio is shown as a function of the length of the gapped segment (∆*L* ^*D*1^, abscissa) and the expected number of indels per site along each branch (different curves). The figure is a reduced and modified version of Figure 5 of [43]

We next studied some typical cases of local MSAs. (The analytical calculations of the multiplication factors are detailed in SM-4 of Supplementary methods in Additional file 1.) We only examined MSAs resulting from the evolution along a 3-OTU tree (Fig. 4a). This is because a next-parsimonious indel history typically differs from its parsimonious counterpart in the sequence state at the internal node that phylogenetically delimits an indel event. We examined the following four cases, which differ in the sets of homologous sites in between the PASs: (I) none of the three sequences has any site (Fig. 4b); (II) two sequences share a homologous run of sites, but the third sequence has no site (Fig. 4c); (III) one sequence has a run of sites, but the other two sequences have no site (Fig. 4d); and (IV) one sequence (*s*_1_) has a run of sites, another sequence (*s*_3_) has no site, and yet another sequence (*s*_2_) shares the homologous sites with *s*_1_ except a contiguous subset of sites it lacks (Fig. 4e). In case (I), similarly to case (i) local PWAs, the total next-parsimonious contribution $\left({\overset{\smile }{M}}_{P(2)}\left[ case\kern0.5em (I)\right]\right)$ was negligibly smaller than the total parsimonious contribution $\left({\overset{\smile }{M}}_{P(0)}\left[ case\kern0.5em (I)\right]\right)$. The comparison in case (II) reduces to that in case (ii) local PWAs, thanks to the phylogenetic consistency condition that the ancestral sequence states must satisfy (e.g., [46, 47]). The next-parsimonious local indel histories in case (III) are classified into two broad types: (A) those that have the same ancestral sequence state as the parsimonious history, and (B) those that have different ancestral states than the parsimonious history. The comparison of the total parsimonious contribution (${\overset{\smile }{M}}_{P(1)}\left[ case\kern0.5em (III);\kern0.5em \varDelta {L}^{D1}\right]$) to the total contribution from type (A) histories $\left({\overset{\smile }{M}}_{P(2)}\left[ case\kern0.5em (III);\kern0.5em (A);\kern0.5em \varDelta {L}^{D1}\right]\right)$ reduces to the comparison in case (iii) local PWAs. Thus, we can focus on the total contribution from type (B) histories $\left({\overset{\smile }{M}}_{P(2)}\left[ case\kern0.5em (III);\kern0.5em (B);\kern0.5em \varDelta {L}^{D1}\right]\right)$. Our numerical analyses showed that this contribution is much smaller than the parsimonious contribution (Fig. 5), even if the branch or the local MSA is quite long. Actually, the relative contribution decreased as the local MSA got longer (Fig. 5). In case (IV), the total next-parsimonious contribution $\left({\overset{\smile }{M}}_{P(3)}\left[ case\kern0.5em (IV);\kern0.5em \varDelta {L}^{D1},\kern0.5em \varDelta {L}^{D2}\right]\right)$ did not substantially exceed 1/2 of the total parsimonious contribution $\left({\overset{\smile }{M}}_{P(2)}\left[ case\kern0.5em (IV);\kern0.5em \varDelta {L}^{D1},\kern0.5em \varDelta {L}^{D2}\right]\right)$ until the local MSA or the branches became quite long (Table 2).^8^

**Table 2:** Perturbation analysis on local MSA probabilities in case (IV)

(∆*L* ^*D*1^, ∆*L* ^*D*2^)	0.01 indels/site	0.04 indels/site	0.1 indels/site	0.2 indels/site
(2, 1)	**0.004**	**0.016**	**0.037**	**0.067**
(3, 1)	**0.016**	**0.063**	**0.150**	**0.279**
(3, 2)	**0.012**	**0.049**	**0.118**	**0.225**
(10, 1)	**0.050**	**0.190**	**0.432**	0.751
(10, 2)	**0.060**	**0.225**	0.509	0.895
(10, 8)	**0.060**	**0.231**	0.543	0.980
(10, 9)	**0.047**	**0.182**	**0.421**	0.740
(30, 1)	**0.140**	**0.487**	0.915	1.203
(30, 5)	**0.163**	0.563	1.118	1.645
(30, 25)	**0.173**	0.620	1.251	1.850
(30, 29)	**0.139**	**0.486**	0.909	1.189
(100, 1)	**0.418**	0.985	1.180	1.304
(100, 99)	**0.418**	0.981	1.170	1.290

Each cell shows the ratio of the total next-parsimonious contribution to the total parsimonious contribution. In each local MSA, the first, second and third sequences have ∆*L* ^*D*1^ sites, ∆*L* ^*D*2^ (<∆*L* ^*D*1^) sites and zero site, respectively, in between the PASs. Each column is labeled with the expected number of indels per site along each of the three branches. The ratios that are less than 0.5 are shown in boldface. This table is identical to Table 3 of [43]. See section M1  of Methods for the parameter setting

Taken together, the results in sections R2-R4 suggest that the first approximation by the parsimonious indel histories alone will estimate the multiplication factor for each local alignment fairly well, as long as the local alignment size and the branch lengths (or the time interval) are at most moderate.

### R5. Simulation analyses to see goodness of first approximation for local MSAs

Thus far, we examined all cases of local PWAs and some typical cases of local MSAs. To study a much wider variety of local MSAs, we developed an algorithm that calculates the first approximation of the probability of a given MSA under a given parameter setting including a phylogenetic tree. Briefly, the algorithm first chops the input MSA into gapped and gapless segments. Second, it attempts to enumerate all parsimonious indel histories that can give rise to each gapped segment (i.e. local MSA) via what we call a “local multi-path downhill search” algorithm. Third, it computes their contributions to the multiplication factor for each gapped segment. And finally, it computes the first-approximate MSA probability as the product of an overall factor and the total parsimonious contributions to the multiplication factors from all gapped segments. (For details on the algorithm, see SM-5 of Supplementary methods in Additional file 1, as well as Additional file 1: Figures S5-S8.)

After manually validating the sub-algorithm to enumerate all parsimonious indel histories (described in [48]), we conducted simulation analyses using Dawg [32]. (See section M2 of Methods for the settings of the simulations.) We created five homogeneous sets of simulated MSAs, namely, sets 1A, 1B, 3P, 3M and 3F. Each of sets 1A and 1B consists of 100,000 MSAs simulated along a three-OTU tree that has equally long branches and is rooted at its sole internal node. The expected number of indels per site along each branch (*E*[*n*_*ID*_]) is 0.01 (small) for set 1A and 0.04 (medium) for set 1B. Sets 3P, 3M and 3F consist of 10,000 MSAs each, which are simulated along the trees of 12 primates, 15 mammals and 9 fast-evolving mammals, respectively (Additional file 1: Figure S9). These sets were designed to mimic typically encountered MSAs of selectively neutral genome sequences whose sequence divergences are small, moderate and large, respectively.^9^

Every simulation started with a random DNA sequence that is 1000 bases long. For reasons of computational time, we excluded local MSAs containing gaps longer than 100 bases. The numbers of subject local MSAs in sets 1A, 1B, 3P, 3M and 3F were 2,676,332, 7,695,575, 397,455, 935,553 and 984,321, respectively. Among them, 0.15 %, 1.38 %, 0.12 %, 0.23 % and 0.49 %, respectively, exhibited non-parsimonious ancestral sequence states. (See SM-6 of Supplementary methods in Additional file 1 for how the MSAs were compared.)

**Fig. 6:**
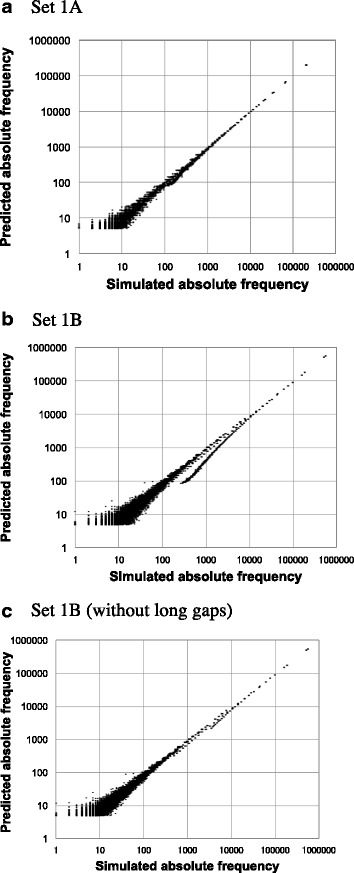
Simulation analyses on absolute alignment frequencies. Each panel compares the predicted absolute frequency of each local homology structure (ordinate) against the number of times that it actually occurred in a simulated dataset (abscissa). The predicted absolute frequency was calculated using Eq. (SM-7.1) in Additional file 1. Note the logarithmic scaling for both axes, which tends to exaggerate sampling errors on the lower-left region in each panel. Panels **a, b** and **c**, respectively, show the results with the simulated sets 1A, 1B and set 1B after removing long gapped segments. The panels a-c are reformatted versions of panels A-C of Figure 29 of [48]

For each of sets 1A and 1B, we compared the absolute occurrence frequency of each of the homology structures of local MSAs with its first approximate prediction (Fig. 6). (See SM-7 in Additional file 1 for how the analysis was performed.) The approximation was pretty good for both set 1A (Fig. 6a) and set 1B (Fig. 6b), with correlation coefficients 0.9996 and 0.9975, respectively (Additional file 1: Table S3). The scatter plot for set 1B (Fig. 6b) showed a thin downward projection from around the middle of the main diagonal, indicating the underestimation of the frequency. However, it disappeared after removing the local MSAs in each of which one or more unobservable indels are expected (Fig. 6c). This extends the conclusions in sections R2 and R3 that the first approximation estimates the probability of a local PWA fairly well as long as its size is within a threshold ((*ΔL*)_0.5_^(*NP*)^ or (*ΔL*)_0.5_^(1)^).

**Fig. 7:**
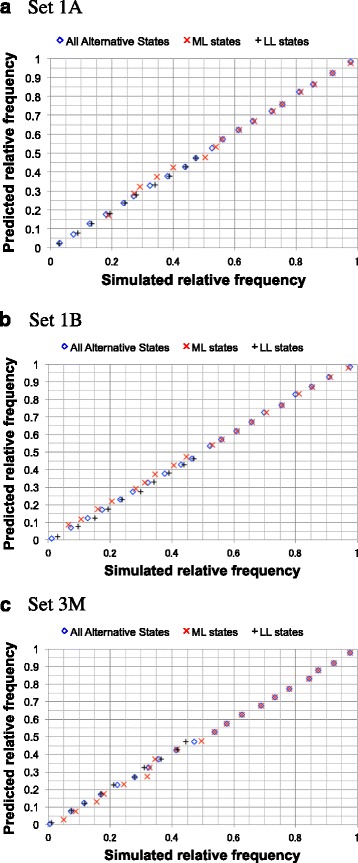
Simulation analyses on relative frequencies among local indel histories. Each panel compares the predicted relative frequencies (ordinate) against the actual relative frequencies in simulations (abscissa). The relative frequencies are among parsimonious local indel histories that potentially yield the same local MSA. A *blue* diamond, a red ‘X’ and a *black* cross represent a bin of all parsimonious local indel histories, that of most likely (ML) parsimonious histories, and that of least likely (LL) parsimonious histories, respectively. Panels **a, b** and **c** show the results with sets 1A, 1B and 3M, respectively. See section SM-8 in Additional file 1 for how the analysis was performed. Panels **a** and **b** are reformatted versions of panels D and E, respectively, of Figure 29 of [48]

Then, for each of simulated sets 1A, 1B, 3P, 3M and 3F, we first examined the relative frequencies of actual occurrences among different sets of parsimonious ancestral states consistent with each local MSA. Then we put the relative frequencies into 20 bins of width 0.05 each. And, finally, we compared the average frequencies in the bins with their first-approximate predictions (Fig. 7 and Additional file 1: Figure S10). The predictions were shown to estimate the actual relative frequencies quite well, with the correlation coefficients ranging from 0.997 to 0.99999 (Additional file 1: Table S4). (See SM-8 in Additional file 1 for how this analysis was done.)

The results in this section suggest that the first approximation would work fairly well also for a majority of local MSAs we are likely to encounter, as long as the local MSA and the branches are at most moderately long.^10^

### R6. Examining other indel models and methods in light of our formulation

One of the major merits of our *ab initio* perturbative formulation is that it can be applied to considerably realistic evolutionary models of indels [22]. Therefore, it will enable us to examine, e.g., the parameter ranges where other indel probabilistic models can well approximate the *ab initio* alignment probability under a fairly realistic evolutionary model.

First we briefly study the goodness of approximation by the geometric indel length distribution, which most of commonly used indel models are based on. As in the previous sections, we use the power-law distribution of the indel length (*l*), *f*_1.6_^*PL*^(*l*) = *l*^− 1.6^/[∑_*k* = 1_^∞^*k*^− 1.6^], as a reference. Then, we fitted the scaled geometric distribution, *f*^*SG*^(*l*; *A*, *q*) = *A* (1 − *q*) *q*^*l* − 1^, to the power-law under a least-square criterion. The best-fit parameters were *A*_*LS*_ = 0.7125 and *q*_*LS*_ = 0.3957. Calculating the ratio, *R*_*LS*_(*l*) ≡ *f*^*SG*^(*l*; *A*_*LS*_, *q*_*LS*_)/*f*_1.6_^*PL*^(*l*), for different indel lengths, we find, e.g., *R*_*LS*_(5) = 0.3168, *R*_*LS*_(7) = 0.08495 and *R*_*LS*_(13) = 8.775 × 10^− 3^. The ratio decreases rapidly as the indel length increases. If, for example, we allow the ratio as small as 1/3, the geometric distribution is regarded as a decent approximation only for *l* ≤ 4. With *f*_1.6_^*PL*^(*l*), the indels with *l* ≥ 5 account for about 30 % of all indels. These mean that, for 30 % of actually occurring indels, the geometric distribution substantially underestimates their frequencies *even according to the above lenient criterion*. This reconfirms the importance of using biologically realistic indel length distributions, as pointed out, e.g., in [23, 24]. 11

Next, as an example of indel models that incorporate some biological realism, we investigate the HMM of Kim and Sinha [36]. Their HMM can accommodate power-law indel length distributions. However, similarly to most other HMMs and transducers, it cannot correctly handle overlapping indels along the same branch, although it can handle overlapping indels along different branches. Another characteristic of their method is that it applies the same indel length distributions to all branches. The behaviors of the “exact” solutions (Fig. 3, Additional file 1: Table S2) indicate that their HMM could approximate the probabilities of local PWAs fairly well in cases (i), (ii) and (iii), as long as branch lengths are reasonable for phylogenetic analyses. Almost the same conclusions were drawn also from the analyses using up to next-parsimonious contributions in the perturbation expansion, Eq. (3). (See SM-9 of Supplementary methods in Additional file 1.) Regarding case (iv) local PWAs, however, our analysis indicated that their HMM could substantially underestimate the *ab initio* probabilities, especially when long indels are involved (Table 3). (How we performed this analysis is also described in SM-9.) This is because their HMM, like most HMMs and transducers, neglects 1/3 of the effects of non-overlapping indels (panels a, b and c of Additional file 1: Figure S3), as well as most effects of overlapping ones (panels d and e), that can yield each case (iv) local PWA. (Briefly, most HMMs neglect one of panels b and c. See SM-9 for details.) These results suggest that a potentially effective way to improve the accuracy of the HMM of Kim and Sinha [36] would be to modify the transition probabilities between a deletion-type block and an insertion-type block. This measure will enable to incorporate the effects of overlapping indels in case (iv).

**Table 3:** Comparison of Kim-Sinha’s probability with *ab initio* probability for case (iv) local PWA

(∆*L* ^*A*^, ∆*L* ^*D*^)	Ratio (= ref/KS) ^a^	Overlapping ^b^
(1, 1)	**1.667**	0.167
(3, 1)	**1.883**	0.383
(3, 3)	2.449	0.949
(5, 5)	3.325	1.825
(10, 1)	2.165	0.665
(10, 10)	5.572	4.072
(25, 1)	2.355	0.855
(25, 4)	4.714	3.214
(30, 10)	8.300	6.800
(100, 1)	2.561	1.061
(100, 3)	4.896	3.396
(300, 1)	2.659	1.159

Each row gives values for a local PWA with ∆*L* ^*A*^ ancestral sites and ∆*L* ^*D*^ descendant sites in between a pair of PASs. See section M1 of Methods for the parameter setting. When *λ*_*I*_ = *λ*_*D*_, the ratio for (*ΔL* ^*A*^, *ΔL* ^*D*^) = (*L*_1_, *L*_2_) is identical to that for (*ΔL* ^*A*^, *ΔL* ^*D*^) = (*L*_2_, *L*_1_). Thus, we only showed the results for *ΔL* ^*A*^ ≥ *ΔL* ^*D*^. The ratios less than 2 are in boldface. This table is a modified version of Table 4 of [43]

^a^ The ratio of the *ab initio* probability to the corresponding probability given by the HMM of Kim and Sinha [36], in the limit where the time interval (or branch length) approaches zero

^b^ The portion of the ratio contributed by overlapping indels (such as those in panels d and e of Additional file 1: Figure S3)

As exemplified above, our *ab initio* perturbative formulation provides other indel probabilistic models with a sound reference point, under which the models can be examined to improve their accuracy and evolutionary consistency. A related topic is the Chapman-Kolmogorov (CK) equation, which must be satisfied by genuine stochastic evolutionary models. Unfortunately, most of the currently common indel probabilistic models violate the CK equation (as argued, e.g., briefly in [41]). Because our perturbative formulation satisfies the CK equation up to any desired order of the perturbation expansion (Appendix A3 of [49]), our formulation could also examine the effects of the violation of the CK equation. For example, under a geometric indel length distribution, the effects become conspicuous only when the indel lengths exceed a “critical value” of *O*(10), where the geometric distribution substantially underestimates the real indel frequencies. (See, e.g., subsection 2.3 of [43].) This seems to explain the results of past studies (e.g., [50, 51]), which did not detect remarkable effects of the violation of the CK equation (or the effects of its cause, i.e., inadequately incorporating overlapping indels).

### R7. Outstanding issues and possible improvements, extensions and applications

Here, we briefly discuss some outstanding issues and their possible solutions in the forms of methodological improvements and extensions, and also possible applications of the (improved/extended) methods to practical problems. (For more details on most of these topics, see Discussion of [48].)

#### R7.1. Possible improvements and extensions of our computational methods and algorithms

In this study, we successfully constructed two integral equation systems to calculate “exact” multiplication factors for case (i), (ii) and (iii) local PWAs. For case (iv) local PWAs (Additional file 1: Figure S1d), we only provided methods to analytically calculate the total parsimonious (i.e., 2-indel) and total next-parsimonious (i.e., 3-indel) contributions. Although in principle we could calculate contributions from indel histories with more than 3 indels each, the question should be how we can do this within a reasonable amount of time. Even if we can construct an integral equation system for case (iv), it is expected to contain terms with complex gap configurations, and thus it would be difficult to solve it “exactly.” Therefore, a key for this case should be how we can reasonably quickly obtain approximate multiplication factors each of which estimates the exact factor more accurately than the summation over all parsimonious and next-parsimonious contributions.

The main purpose for the current algorithm to calculate the first-approximate probability of a given MSA (SM-5 in Additional file 1) was to see whether or not the first approximation works also for local MSAs of general types. This algorithm merely constitutes the first step toward an automatic application of our *ab initio* perturbative formulation. Consequently, the algorithm still has some rooms for improvements. For example, the algorithm could be very slow when applied to a local MSA containing a long gap. Roughly speaking, the length of time consumed by the algorithm applied to an input MSA is the summation of the lengths of the consumed time over all local MSAs in it. We estimated that the algorithm applied to each local MSA has the time complexity roughly greater than $O\left(B\;{2}^{N_{ShG}}\right)$. (See subsection D1.1 of [48] for details.) Here, *B* is the number of blocks of distinct gap patterns in the local MSA, and *N*_*ShG*_ is the number of short gaps that are spatially overlapping and phylogenetically neighboring the long gap. For example, if *B* = *N*_*ShG*_ = 20, the time complexity is greater than *O*(20 × 2^20^) ≈ *O*(10^7^). *B* should be roughly on the order of *N*_*ShG*_. And *N*_*ShG*_ is roughly expected to be around *E*[*N*_*ShG*_] ≈ (*E*[*n*_*ID*1_] + *E*[*n*_*ID*2_]) × *ΔL*. Here *E*[*n*_*ID*1_] and *E*[*n*_*ID*2_] are the expected numbers of indels per site along the two neighboring branches of the branch where the indel resulted in the long gap. (These three branches share the node that accommodates the subsequence aligned with the long gap.) And ∆*L* is the size of the local MSA. For example, if we assume a rather large value, *E*[*n*_*ID*1_] = *E*[*n*_*ID*2_] = 0.4/8 = 0.05, we have *E*[*N*_*ShG*_] ≈ 0.1 × *ΔL*. In this case, we expect *N*_*ShG*_ ≈ 20 almost always when *ΔL* = 200. Thus, such local MSAs could virtually stop the current algorithm. 12 One way to quickly process a local MSA containing long gaps should be to treat the gaps hierarchically, first long gaps alone and second the remaining short and medium gaps (Additional file 1: Figure S11; see Discussion D1.1 of [48] for more details). If this strategy indeed works, the $O\left({2}^{N_{ShG}}\right)$ component of the time complexity would reduce to *O*(2*N*_*ShG*_), because the short gaps that are phylogenetically neighboring the long gap can be handled independently of one another (panel d of Figure S11). Another very similar strategy should be to narrow down the ancestral sequence states to be searched for, *regardless of the presence/absence of gapless columns*, by exploiting the “phylogenetic correctness” condition (e.g., [46, 47]). The condition must always be satisfied by the ancestral sequence states consistent with MSAs, and thus it should be very powerful.

Another possible improvement should be to incorporate non-parsimonious indel histories so that we can enhance the accuracy of the probability estimation. As in section R4, we can classify non-parsimonious histories into two broad categories: (A) those each of which shares the set of all ancestral sequence states with a parsimonious history, and (B) those that share the set with no parsimonious history. Each non-parsimonious history in category (A) yields the same ancestor-descendant PWAs along all branches as a parsimonious history does (section R7 of [22]). Hence, we could easily incorporate the effects of category (A) histories by using local PWA multiplication factors that take account of non-parsimonious contributions, as we calculated in sections R2 and R3. As the result in section R4 (Fig. 5) indicates, this could considerably improve the accuracy relatively easily. Incorporating histories in category (B) should require an algorithm to systematically enumerate such histories. Some hints may come from the examples in section R4 and SM-4 of Supplementary methods in Additional file 1, and the “branch-and-merge” operation (SM-5.2 of Supplementary methods). The real challenge, though, should be to devise a method to enumerate such histories efficiently. 13

In this paper, we presented the results of computing local alignment probabilities (or multiplication factors) *under a space-homogeneous model* implemented in Dawg [32], mainly in order to avoid excessive presentational complications. At least theoretically, however, the computational methods (in Additional file 1) could be extended to *space-heterogeneous* situations relatively easily. All we have to do is substitute space-heterogeneous counterparts for the space-homogeneous indel rates (and exit rates) in the final formulas in SM-2, SM-3 and SM-4 (in Additional file 1), and replace multiplication by some integer factors (such as (*ΔL*^*A*^ + 1) in Eq. (SM-3.2) and Eq. (SM-3.4b) in Additional file 1) with summation over possible positions (of a position-flexible indel event). The time integration can be performed analytically (except in SM-3) as long as the model is time-homogeneous. And the computation could be automated as long as the indel rates are specified according to some programmable rules. In some cases, tricks or approximations may be necessary so that the computation (involving the aforementioned summations) can be finished quickly enough. It should be kept in mind, however, that such computation will make sense *only if* the probabilities of the entire alignments are factorable. This means that the indel rates (and the exit rates) must satisfy the conditions (i) and (ii) (and (iii) for MSAs) explained in section R1, which may bring in some complications. For example, in the most general indel model we currently know to have factorable alignment probabilities (described in subsection R8-3 of [22]), each *locally* heterogeneous set of indel rates is confined in a region that *does not necessarily* coincide perfectly with a gapped segment (i.e., a local alignment). When the region accommodates only one gapped segment (and some gapless columns), there should be no serious problem; although each position between contiguous gapless columns may experience some indels, the effects of such indels should be negligible (as shown in sections R2 and R3), allowing us to focus on the single gapped segment. On the other hand, a serious problem may arise when the region accommodates two or more gapped segments. In this case, the contributions from the gapped segments (overlapping the region) can no longer be factorized, and thus all possible relative orders will have to be considered among indels in different gapped segments overlapping the region (while retaining the order in each segment). This could substantially slow down the computation, especially regarding non-parsimonious indel histories (including practically exact solutions), and some new measures may be necessary for reasonably fast computation. In addition, it should be remembered that, in order to pursue further biological realism, one must also overcome some other hurdles, such as more realistic boundary conditions and mutations other than indels and substitutions (discussed, e.g., in section R9 of [22] and [37]).

#### R7.2. Risks associated with naive applications to reconstructed alignments

Some readers may consider conducting some evolutionary analyses by applying the algorithm presented here to a MSA reconstructed by one of the state-of-the-art aligners (reviewed, e.g., in [7]). We strongly caution the readers that it would be premature to conduct such analyses at this point. What we demonstrated here is that the algorithm estimates the probabilities quite accurately, *provided that it is fed a correct MSA*. Unfortunately, however, recent analyses (e.g., [38, 52, 53]) showed that reconstructed MSAs are considerably error-prone, even if they were reconstructed via state-of-the-art aligners. Thus, a naive application of the algorithm to a *reconstructed* MSA would likely lead to incorrect predictions. Therefore, the readers should avoid such analyses whenever possible. Even if they need to perform such analyses, the possibility of MSA errors must be fully taken into account when interpreting the results.

#### R7.3. Possible applications

Originally, we developed our theoretical formulation [22] and the algorithm presented here for the purpose of comparing candidate MSAs in terms of their occurrence probabilities, i.e., their likelihoods. This purpose should be adequately fulfilled considering the accuracy of the predicted probabilities under moderate conditions, as demonstrated in this paper. If the algorithm can be coupled with a sampler that can preferentially explore quite likely regions of the MSA space, we could obtain an approximate probability distribution of MSAs. Such a distribution should be very useful, because a substantial fraction of alignment errors turned out to be due to the stochastic nature of evolutionary processes [38]. In the previous study [22], we showed that the “long indel” model [26] is virtually equivalent to our *ab initio* formulation under space-homogeneous indel rates. Hence, their dynamic programming (DP) could be applicable to the problem, possibly with some modifications. Although the full version of their DP is quite slow, a device similar to those used recently (e.g., [21, 41, 54]) might speed up the MSA space exploration. It remains to be seen if such a device could be successfully adapted to our formulation or not. Most of currently available MSA aligners, whether they implement probabilistic or single-optimum-search algorithms, are based on geometric distributions. Because biologically realistic indel length distributions were shown to improve the accuracy of pairwise sequence comparisons (e.g., [23, 24]), we expect that this will be the case also with multiple sequence comparisons. (This expectation was partially confirmed in [38].)

Up to here, we assumed that the phylogenetic tree is given. In many cases, however, the phylogenetic trees must also be inferred from the input sequence data. A theoretically ideal way would be to infer the joint distribution of MSAs and phylogenetic trees, as it is expected to minimize possible prediction biases (e.g., [18, 39, 55, 56]). A major problem is that such an analysis would be very time-consuming in general. In this sense, the traditional method of inferring the phylogenetic tree from an input MSA (e.g., [5]) and the incorporation of indel information into the method (e.g., [57]) would still be useful. When trying to adapt our algorithm or formulation to any of these methods, we will have to further speed up the calculation of approximate alignment probabilities, especially under the moves in the tree space exploration, such as the nearest-neighbor interchange (NNI) and the sub-tree pruning and re-grafting (SPR). At present, it is a totally open question whether we can really do this without compromising the accuracy of the predicted probabilities. This should be a challenging, formidable yet crucial problem of phylogenetic-level molecular evolution.

## Conclusions

In the previous study [22], we proposed a theoretical formulation that facilitates the *ab initio* calculation of the probabilities of given PWAs and MSAs under the general continuous-time Markov model, which describes the evolution of an *entire* sequence along a time axis via indels. And we explicitly demonstrated that, under a certain set of conditions, each *ab initio* alignment probability is factorable into the product of an overall factor and multiplication factors originated from local alignments delimited by preserved ancestral sites, thus providing a sort of generalized HMMs (Eq. (2) and Eq. (5)).

In this study (especially in Supplementary methods in Additional file 1), we provided some methods and an algorithm to concretely calculate the total parsimonious contribution and the total next-parsimonious contribution to the multiplication factor, Eq. (3) or Eq. (6), originated from each local alignment, under space-homogeneous situations for illustration purposes. Our analyses indicated that even the total parsimonious contribution approximates the multiplication factor fairly well as long as (*λ*_*I*_ + *λ*_*D*_)(*t*_*F*_ − *t*_*I*_)*ΔL* is within an *O*(1) threshold. Here, (*λ*_*I*_ + *λ*_*D*_)(*t*_*F*_ − *t*_*I*_) is the expected number of indels per site and ∆*L* is the local alignment size. Moreover, again under space-homogeneous situations, we deduced two systems of integral equations that can be numerically solved to give practically exact multiplication factors for local PWAs of cases (i), (ii) and (iii). An inspection of the practically exact factors indicated that the finite-time transition probabilities in these local PWAs keep following the power-law, and that the exponent only slightly deviates from the original exponent for the instantaneous indel length distribution. Equipped with these results and new methods, the theoretical formulation we proposed in [22] is expected to provide other indel probabilistic models with a sound reference point, which could suggest necessary modifications to improve the accuracy of the models (as exemplified in section R6).

However, considering that the commonly used aligners are considerably error-prone (as shown e.g., in [38, 52, 53]), it would be very risky to naively apply the presented algorithm or methods to *reconstructed* MSAs. Thus, it should be preferable to first develop programs that exploit the fruits of the previous study [22] and this study to accurately estimate the uncertainties in, and rectify the errors of, reconstructed alignments under a genuine stochastic model of sequence evolution via indels that is biologically more realistic than almost all models studied in the past.

## Methods

### M1. Parameter settings for numerical analyses

We performed all numerical analyses in this paper using the space-homogeneous indel model implemented in Dawg [32] (see Eqs.(7-9)). Unless otherwise stated, the total insertion rate was set equal to the total deletion rate (that is, *λ*_*I*_ = *λ*_*D*_), according to a genome-wide data analysis [41]. We used the power-law indel length distribution for both insertions and deletions: ${f}_I(l)\kern0.5em =\kern0.5em {f}_D(l)\kern0.5em =\kern0.5em {f}_{1.6}^{PL}\left(l;\kern0.5em {L}^{CO}\right)\kern0.5em \equiv \kern0.5em {l}^{-1.6}/\left({\displaystyle {\sum}_{k=1}^{L^{CO}}{k}^{-1.6}}\right)$. The power-law exponent of 1.6 is among the typical values observed empirically (e.g., [24] and references therein). The cut-off indel length, *L*^*CO*^, was set at 500 sites for the perturbation analyses to assess the goodness of the first approximation (Tables 1 and 2, Figs. 2, 3 and 5; Additional file 1: Tables S1, S2 and Figure S4), whose results were almost independent of *L*^*CO*^. It was set at 5000 sites when assessing the goodness of approximation by the HMM of Kim and Sinha [36] (Table 3), because the result stabilized around this value of *L*^*CO*^. In the perturbation analyses on local MSAs (e.g., Table 2 and Fig. 5), we used a 3-OTU tree with equally long branches (Fig. 4a). The tree was rooted at its sole internal node. For the iterative perturbation analysis (Figs. 2 and 3; Additional file 1: Tables S1 and S2), the sub-time-interval for the numerical time integration was set at *E*[*n*_*ID*_]/*N*_*P*_ = 0.001 (indels per site). 14 For local MSA analyses (both perturbative and simulation-based), the uniform sequence length distribution was employed as the prior probability of the root sequence state.

### M2. Simulations to prepare input MSA sets

To validate the entire algorithm described in section SM-5 of Supplementary methods in Additional file 1, we prepared five sets of MSAs using a genuine sequence evolution simulator, Dawg [32]. We performed all simulations using the same Zipf power-law distribution, ${f}_D\;(l)\kern0.5em =\kern0.5em {f}_I\;(l)\kern0.5em =\kern1em {l}^{-\gamma }/\left({\displaystyle {\sum}_{k=1}^{L^{CO}}{k}^{-\gamma }}\right)$, with the exponent *γ* = 1.6 and the cut-off indel length of *L*^*CO*^(=*L*_*I*_^*CO*^ = *L*_*D*_^*CO*^) = 100 bases. The exponent *γ* = 1.6 is typical among empirically observed values (e.g., [24] and references therein). The cut-off length was chosen in order to prevent it from taking extremely long to search for parsimonious local indel histories. This is because, with our current implementation, the search could be very time-consuming when a gapped segment contains at least one long gap (see subsection R7.1 of Results and discussion for a possible solution). Each of the simulations started with a random ancestral DNA sequence that is l000 bases long. In each simulation, we labeled all the internal nodes of the input tree, in order to keep the ancestral sequences aligned with the “extant” sequences (at the external nodes). Other parameters and options were set at default values unless otherwise stated. We created five input MSA sets, namely, 1A, 1B, 3P, 3M and 3F.

**Set 1A** consists of 100,000 MSAs, each of which was simulated along a 3-taxon tree starting at a root with three child branches. The lengths of the three branches were all set at 0.05 (substitutions per base). The total rates of insertions and deletions were set at *λ*_*I*_ = *λ*_*D*_ = 0.1 (per expected substitution), which are close to the upper-bounds for neutrally evolving mammalian DNA sequences [24, 35].

**Set 1B** is nearly the same as Set 1A, expect that all branch lengths were set at 0.2 (substitutions per base).

We prepared 1A and 1B, because validating the theoretically predicted occurrence probabilities of local homology structures necessitated a large number of MSAs simulated under identical parameter settings.

Each of **Sets 3P**, **3M** and **3F** consists of 10,000 MSAs. The settings for these three sets differ only in the phylogenetic tree used for the simulations. The MSAs in these sets were simulated along the tree of 12 primates (panel a of Additional file 1: Figure S9), the tree of 15 mammals (panel b) and the tree of 9 fast-evolving mammals (panel c), respectively. These three sets, 3P, 3M and 3F, were intended to mimic typically encountered MSAs among selectively neutral DNA sequences with small, moderate and large sequence divergences, respectively. The total indel rates for these three sets were set at *λ*_*I*_ = *λ*_*D*_ = 1/16 = 0.0625 (per expected substitution), according to genome-wide data analyses [35, 41]. For more details on these three sets, see [38].

The Dawg control files used to generate these simulated datasets, including the phylogenetic trees and indel model parameters, are available as a part of Additional file 2.

Before the analyses, all simulated MSAs were pre-processed so that the MSAs with an identical homology structure will be replaced with a unique representative MSA. See Methods of [38] for details.

### M3. Program implementation

The Perl modules and main Perl scripts used in this study are available (under the GNU General Public License) as a package named “LOLIPOG” (for “LOg-LIkelihood for the Pattern Of Gaps (in MSA)”) (version: “FA_LOLIPOG_P.ver0.6.1.6”), which is archived in Additional file 2. The latest version of the package will be available in the “lolipog” directory at the FTP repository of http://Bioinformatics.Org [58].

## Additional files

Additional file 1: Supplementary methods (sections SM-1 through SM-9), Tables S1 through S4, and Figures S1 through S11. The sections of Supplementary methods describe methodological details on the (analytical and algorithmic) computations of local alignment probabilities (SM-1,2,3,4,5,9) and on the simulation analyses for validating the algorithm (SM-6,7,8). Tables S1-S4 show the results of some analyses. Figures S1, S2, S3 and S11 illustrate some important concepts. Figures S4 and S10 show the results of some analyses. Figures S5, S6, S7 and S8 explain the algorithm implemented in LOLIPOG. Figure S9 shows the phylogenetic trees used for the simulations. (PDF 12355 kb) Additional file 2: A package (the version used for the analyses in this paper), which contains Perl modules that implement the key algorithms and formulas, as well as some main Perl scripts that we used for the actual data analyses. (The package is available under the GNU General Public License. The modules and scripts will run on a Mac OS X terminal, and were confirmed to run also on Red Had Enterprise Linux (6.4). And they will probably run on other UNIX platforms as well, although we have not tested whether they indeed do.) The package also contains Dawg control files necessary for creating the simulated MSA sets 1A, 1B, 2, 3P, 3M and 3F. The latest version of the package (“FA_LOLIPOG_P.verxxx”) will be available from the “LOLIPOG” (LOg-LIkelihood for the Pattern Of Gaps) project at the FTP repository of the Bioinformatics Organization [58]. (ZIP 7548 kb)

## Footnotes

1 An ancestry index (or an “ancestry” for short) is assigned to each site, in order to distinguish between the sites of different evolutionary origins. Once an ancestry is assigned to a site, it will not change throughout the evolutionary history. Sharing of the same ancestry between the sites of different sequences indicates that the sites are mutually homologous (more precisely, orthologous). See section R2 of [22] for more details. The ancestry indices also help realize the rate heterogeneity across regions (section R3 of [22]).

2 In this study, the homology structures we consider will usually be among extant sequences, i.e., excluding ancestral sequences. When dealing with each particular indel history, however, ancestral sequences will also be included.

3 This “perturbative” calculation of a multiplication factor may look similar to the calculation of a “chop-zone” probability proposed by Miklós et al. [26] under their “long indel” model. In fact, our “perturbative” calculation could be regarded as an (modified) extension of their calculation method to somewhat more general evolutionary models. (See section SA-3 of Additional file 2 of [22] for their mutual equivalence with caveats.)

4 For example, the results under the “long indel” model [26] are essentially the same as those obtained here, if the former is freed from the time-reversibility.

5 It should be noted that this setting is just a representative of seemingly common situations. For example, the empirically estimated power-law exponents vary between near 1 and near 2, although the values within 1.4-1.8 are quite common ([24] and references therein), regardless of whether the sequences are genomic or protein-coding. In the past, the deletion rate was often observed as greater than the insertion rate (e.g., [59]). But a simulation study [52] indicated that such observations are likely due to a bias intrinsic to the similarity-based aligners (as classified in [60]). And a recent analysis of mammalian sequences via an evolution-based probabilistic aligner (expected to be devoid of the above bias) indicated that the deletion rate is nearly equal to the insertion rate [41]. It remains to be seen whether this rate equality applies also to the taxa other than mammals. Regarding the sequence length distribution, we do not know any empirical results. Although we believe that our choice of the uniform distribution should be theoretically very reasonable for neutrally evolving regions sampled randomly from long genomes/chromosomes (see endnote 14 of [22]), distributions of regions under selection may show quite different behaviors. It should be interesting, and important, to examine how the results in this paper will change in response to the deviation of the setting from that given here.

6 These cases correspond to the “chop-zones” having probabilities *N*_*ij*_ [26], with: (i) *i* = *j* = 0; (ii) *i* > 0, *j* = 0; (iii) *i* = 0, *j* > 0; and (iv) *i* > 0, *j* > 0.

7 Because of the symmetry between the finite-time transition probabilities under the time reversal, the latter results become equal to the former when the insertion and deletion parameters are swapped.

8 The analytical expressions are somewhat complex in case (IV). See Appendix A2 of [43] for details.

9 On top of them, we also prepared yet another set, Set 2, created by simulations under 33 sets of parameters typical of the structure-based benchmark MSAs. (See section M2.2 of Methods and Figure 28, both of [48], for details on this input MSA set.) The validation analyses on this Set 2 gave nearly as good results as those on the 5 sets described here. (See Results of [48] for details.)

10 Incidentally, for the analysis on relative probabilities, we only used local MSAs each of which can result from two or more parsimonious local indel histories. The instances of such gapped segments accounted for 4.5 %, 12.0 %, 17.5 %, 19.2 % and 14.2 % of all “parsimonious” instances in sets 1A, 1B, 3P, 3 M and 3 F, respectively. Out of them, the most likely (ML) histories were wrong in 42.3 %, 43.4 %, 36.1 %, 13.1 % and 38.4 % of the instances in the respective sets. Therefore, even if we make an unlikely assumption that the aforementioned “non-parsimonious” instances were all due to the ML histories that are non-parsimonious, any algorithm that searches for a single ML history would have overlooked the true indel history in 1.9 %, 5.2 %, 6.3 %, 2.5 % and 5.4 % of the cases in the respective sets. These frequencies are much larger than those of the “non-parsimonious” instances, i.e.*,* 0.15 %, 1.4 %, 0.12 %, 0.23 % and 0.49 %, respectively. This indicates that, given correct MSAs and correct trees, our algorithm can recover the true indel histories more frequently than any algorithm to search for a *single* ML history.

11 Mixed geometric distributions could decently approximate a power-law distribution in wider ranges. For example, the approximation by a mixed distribution with two geometric components (used, e.g., in [23]) is fairly good up to a few dozen residues, but it gets poor when indels become as long as hundreds of residues. To decently approximate the realistic distributions of such long indels, more geometric components would be necessary.

12 In SM-8 of Supplementary methods of [38], we roughly estimated the expected mean length $\left({\overline{L}}_{loc}(T)\right)$ of a *correct* (i.e., not reconstructed) local MSA as: ${\overline{L}}_{loc}(T)\kern0.5em \approx \kern0.5em \left[ exp\left(\left|T\right|\kern0.5em {\lambda}_D\kern0.5em {\overline{l}}_D\right)\kern0.5em +\kern0.5em  exp\left(\left|T\right|\kern0.5em {\lambda}_I\kern0.5em {\overline{l}}_I\right)\kern0.5em -\kern0.5em 2\right]/\;\left[1- exp\left(-\left|T\right|\kern0.2em \left({\lambda}_D\kern0.5em +\kern0.5em {\lambda}_I\right)\right)\right]$. Here, |*T*| is the total branch length across the tree (*T*) (in units of the expected number of substitutions per site). *λ*_*D*_ and *λ*_*I*_, respectively, denote the deletion rate and the insertion rate, both per expected substitution. And ${\overline{l}}_D$ and ${\overline{l}}_I$ are the average lengths of a deleted subsequence and an inserted subsequence, respectively. Under the parameter setting for the simulation analyses of this study, we have ${\overline{l}}_D\kern0.5em =\kern0.5em {\overline{l}}_I\kern0.5em =\kern0.5em 6.35$. If we set *λ*_*D*_ = *λ*_*I*_ = 1/16 = 0.0625 according to [35, 41], then, ${\overline{L}}_{loc}(T)$ exceeds 100 when |*T*| is greater than 9, which is quite large. Thus, *as long as we are dealing with correct MSAs*, our first-approximate algorithm is expected to work on a majority of local MSAs until |*T*| becomes this large. It should be noted, however, that *reconstructed* MSAs could be seriously erroneous even if |*T*| is, e.g., less than 3 [38].

13 Strictly speaking, the current “local-multi-path downhill search” algorithm is not perfect, in the sense that it misses some deletion-dominated parsimonious local histories that can yield gap configurations belonging to the class of “intersection between cousins” described in [61]. Fortunately, this drawback is not expected to be so serious, because such local histories require at least four indels each and thus should be very rare.

14 We calculated the multiplication factors, ${\tilde{\mu}}_P^{\left\langle {N}_{ID}=200\right\rangle}\left[ case\kern0.5em (iii);\kern0.5em \varDelta {L}^D;\kern0.5em \left[{t}_I,\kern0.5em t\right]\right]$ with *t* ∈ [*t*_*I*_, *t*_*F*_], using *E*[*n*_*ID*_]/*N*_*P*_ = 0.001, and compared them to those calculated using *E*[*n*_*ID*_]/*N*_*P*_ = 0.0005. In each pair of values of ∆*L*^*D*^ and *t*, the difference was within 0.2 % of the multiplication factor itself. Thus we concluded that they are virtually exact.
