# Supplementary material for: General continuous-time Markov model of sequence evolution via insertions/deletions: local alignment probability computation
Source: BMC Bioinformatics. 2016 Sep 27;17:397. doi: 10.1186/s12859-016-1167-6 (PMC5039815; doi:10.1186/s12859-016-1167-6)
Supplement: Additional file 1: — Supplementary methods (sections SM-1 through SM-9), Tables S1 through S4, and Figures S1 through S11. The sections of Supplementary methods describe methodological details on the (analytical and algorithmic) computations of local alignment probabilities (SM-1,2,3,4,5,9) and on the simulation analyses for validating the algorithm (SM-6,7,8). Tables S1-S4 show the results of some analyses. Figures S1, S2, S3 and S11 illustrate some important concepts. Figures S4 and S10 show the results of some analyses. Figures S5, S6, S7 and S8 explain the algorithm implemented in LOLIPOG. Figure S9 shows the phylogenetic trees used for the simulations. (PDF 12355 kb) [file 12859_2016_1167_MOESM1_ESM.pdf]

Additional file 1 of
"General continuous-time Markov model of sequence evolution via insertions/deletions: Local alignment probability computation"

by Kiyoshi Ezawa

Table of contents

Supplementary methods **pp. 2-39**

SM-1. Perturbation expansion of multiplication factor for local alignment: details pp. 2-7

SM-2. Analytical expressions of parsimonious and next-parsimonious contributions (1): for local PWAs pp. 7-12

SM-3. Integral equation system for "exact" solutions of multiplication factors for local PWAs pp. 12-17

SM-3.1. Fitting power-law to finite-time transition probabilities pp. 16-17

SM-4. Analytical expressions of parsimonious and next-parsimonious contributions (2): for local MSAs pp. 17-23

SM-5. Algorithm to compute first-approximate MSA probability pp. 23-30

SM-5.1. Outline pp. 24-25

SM-5.2. Enumerating all parsimonious local indel histories pp. 25-28

SM-5.2.1. Assigning virtual temporal directions and ordering indel events pp. 27-28

SM-5.3. First-approximate calculation of absolute occurrence probability and relative probabilities pp. 28-30

SM-6. Comparing parsimonious local indel histories with true history pp. 30-31

SM-7. Correlation analysis to validate predicted absolute occurrence probabilities of gapped segments pp. 31-32

SM-8. Correlation analysis to validate predicted relative probabilities among parsimonious local indel histories pp. 32-33

SM-9. Accuracy of HMM of Kim and Shinha applied to case (iv) local PWAs pp. 33-39

Additional references **p. 40**

Supplementary tables **pp. 41-46**

Supplementary figures (with legends) **pp. 47-58**

Supplementary methods

As we noted in [22], a bra-vector (*e.g.*, $\langle s|$), a ket-vector (*e.g.*, $|s'\rangle$), and a linear operator (*e.g.*, $\hat{M}$) could be considered as the convenient reminders of a row vector, a column vector, and a matrix, respectively, in the normal formulation of a continuous-time Markov model, if you want. Especially, the operator, $\hat{M}_I(x,l)$, represents the insertion of l sites between the x-th and ($x+1$)-th sites, and the operator, $\hat{M}_D(x_B, x_E)$, represents the deletion of the subsequence between (and including) the x_B-th and x_E-th sites.

SM-1. Perturbation expansion of multiplication factor for local alignment: details

Here, we give details of the mathematical expressions in section R1 of the main Results and discussion, from the beginning down to Eq.(R1.6).

As in section R1, let $P\left[\left(\alpha(s^A,s^D),[t_I,t_F]\right)\middle|\,(s^A,t_I)\right]$ be the probability that a PWA ($\alpha(s^A,s^D)$) between an ancestral sequence state (s^A) and a descendant (s^D) result from evolution during a time interval ($[t_I,t_F]$), given s^A at t_I. (We ignore the residue states.) In [22], we formally proved that $P\left[\left(\alpha(s^A,s^D),[t_I,t_F]\right)\middle|\,(s^A,t_I)\right]$ is given as a series:

$$P\left[\left(\alpha(s^A,s^D),[t_I,t_F]\right)\middle|\,(s^A,t_I)\right] = \sum_{N=N_{\min}\left[\alpha(s^A,s^D)\right]}^{\infty} P_{(N)}\left[\left(\alpha(s^A,s^D),[t_I,t_F]\right)\middle|\,(s^A,t_I)\right],$$

--- Eq.(SM-1.1)

(It corresponds to Eq.(R1.1).) Here, $N_{\min}\left[\alpha(s^A,s^D)\right]$ is the minimum number of indels required to create $\alpha(s^A,s^D)$. The term $P_{(N)}\left[\left(\alpha(s^A,s^D),[t_I,t_F]\right)\middle|\,(s^A,t_I)\right]$ is the fraction of $P\left[\left(\alpha(s^A,s^D),[t_I,t_F]\right)\middle|\,(s^A,t_I)\right]$ contributed from all N-event indel histories that can result in $\alpha(s^A,s^D)$. Let $\mathrm{H}^{ID}\left[N;\alpha(s^A,s^D)\right]$ be the set of such N-event indel histories, and let $[\hat{M}_1,\hat{M}_2,\cdots,\hat{M}_N]$ be an N-event indel history, where each $\hat{M}_\nu$ ($\nu=1,2,...,N$) is an operator representing the ν-th indel event in the history. Then, the above term is expressed as:

$$P_{(N)}\left[\left(\alpha(s^A,s^D),[t_I,t_F]\right)\middle|\,(s^A,t_I)\right] \;=\; \sum_{\substack{[\hat{M}_1,\hat{M}_2,\cdots,\hat{M}_N]\\ \in \mathrm{H}^{ID}\left[N;\,\alpha(s^A,s^D)\right]}} P\left[\left([\hat{M}_1,\hat{M}_2,\cdots,\hat{M}_N],[t_I,t_F]\right)\middle|\,(s^A,t_I)\right].$$

--- Eq.(SM-1.2)

Each term on the right hand side of this equation is given as:

$$P\left[\left([\hat{M}_1,\hat{M}_2,\cdots,\hat{M}_N],[t_I,t_F]\right)\middle|\,(s^A,t_I)\right]$$

$$= \int\cdots\int_{t_I=\tau_0<\tau_1<\cdots<\tau_N<\tau_{N+1}=t_F} d\tau_1\cdots d\tau_N \left(\prod\nolimits_{\nu=1}^{N} r(\hat{M}_\nu;\,s_{\nu-1},\tau_\nu)\right)\,\exp\left\{-\sum_{\nu=0}^{N}\int_{\tau_\nu}^{\tau_{\nu+1}} d\tau\, R_X^{ID}(s_\nu,\tau)\right\}\Bigg|_{\substack{s_0=s^A,\\ \left\{\langle s_\nu|=\langle s_{\nu-1}|\hat{M}_\nu \,|\, \nu=1,\ldots,N\right\}}}.$$

--- Eq.(SM-1.3)

Here, $r(\hat{M}_\nu;\,s_{\nu-1},\tau_\nu)$ is the (generally time- and state-dependent) rate that the sequence state ($s_{\nu-1}$) undergoes the indel ($\hat{M}_\nu$) at time τ_ν, and $R_X^{ID}(s_\nu,\tau)$ is the (generally time- and state-dependent) exit rate of the state (s_ν) at time τ. **These equations, Eq.(SM-1.2) and Eq.(SM-1.3), are essential when we calculate the probability terms under specific situations.**

Now, using some (but not necessarily all) preserved ancestral sites (PASs), we partition the PWA, $\alpha(s^A,s^D)$, into "local regions" (*i.e.*, inter-PAS regions), $\gamma_1,\gamma_2,\ldots,\gamma_{\kappa_{\max}}$, in which all potentially causative indels are confined. In [22], we derived the two conditions.

Condition (i): each indel rate parameter is independent of the portion of the sequence state outside of the local region where the indel occurred; and

Condition (ii): the increment of the exit rate due to each indel event is independent of the portion of the sequence state outside of the local region where the indel occurred.

Under these conditions, the above PWA probability, Eq.(SM-1.1) supplemented with Eqs.(SM-1.2,3), can be factorized as:

$$P\left[\left(\alpha(s^A,s^D),[t_I,t_F]\right)\middle|\,(s^A,t_I)\right]$$
$$= P\left[\left([\,],[t_I,t_F]\right)\middle|\,(s^A,t_I)\right]\prod_{\kappa=1}^{\kappa_{\max}}\tilde{\mu}_P\left[\gamma_\kappa;\left(\alpha(s^A,s^D),[t_I,t_F]\right)\middle|\,(s^A,t_I)\right]. \quad \text{--- Eq.(SM-1.4)}$$

(It corresponds to Eq.(R1.2).) Here, $P\left[\left([\,],[t_I,t_F]\right)\middle|\,(s^A,t_I)\right]=\exp\left\{-\int_{t_I}^{t_F} d\tau\, R_X^{ID}(s^A,\tau)\right\}$ is the probability that the sequence underwent no indels during $[t_I,t_F]$, given s^A at t_I. And $\tilde{\mu}_P\left[\gamma_\kappa;\left(\alpha(s^A,s^D),[t_I,t_F]\right)\middle|\,(s^A,t_I)\right]$, which was denoted as

$\tilde{\mu}_P\left[\left(\tilde{\Lambda}^{ID}\left[\gamma_\kappa;\alpha(s^A,s^D)\right],[t_I,t_F]\right)\middle|\,(s^A,t_I)\right]$ in [22], is the multiplication factor contributed from

the local region, γ_κ. The multiplication factor is a summation of contributions over all local indel histories that can yield the local PWA confined in γ_κ. (The symbol, $\tilde{\Lambda}^{ID}\left[\gamma_\kappa; \alpha(s^A, s^D)\right]$, denotes the set of all such local indel histories.) Thus, it can also be expressed as a series similar to Eq.(SM-1.1):

$$\tilde{\mu}_P\left[\gamma_\kappa; \left(\alpha(s^A, s^D), [t_I, t_F]\right) \middle| (s^A, t_I)\right] = \sum_{N=N_{\min}\left[\alpha(s^A, s^D);\gamma_\kappa\right]}^{\infty} \mu_{P(N)}\left[\gamma_\kappa; \left(\alpha(s^A, s^D), [t_I, t_F]\right) \middle| (s^A, t_I)\right].$$

--- Eq.(SM-1.5)

(It corresponds to Eq.(R1.3).) Here, $N_{\min}\left[\alpha(s^A, s^D); \gamma_\kappa\right]$ is the minimum required number of indels in γ_κ. And the term $\mu_{P(N)}\left[\gamma_\kappa; \left(\alpha(s^A, s^D), [t_I, t_F]\right) \middle| (s^A, t_I)\right]$ is the portion of the multiplication factor contributed from all local-PWA-consistent N-indel local histories in γ_κ. Letting $\Lambda^{ID}\left[N; \gamma_\kappa; \alpha(s^A, s^D)\right]$ be the set of all such local histories, the term is expressed as:

$$\mu_{P(N)}\left[\gamma_\kappa; \left(\alpha(s^A, s^D), [t_I, t_F]\right) \middle| (s^A, t_I)\right] = \sum_{\substack{[\hat{M}_1, \hat{M}_2, \cdots, \hat{M}_N] \\ \in \Lambda^{ID}\left[N; \gamma_\kappa; \alpha(s^A, s^D)\right]}} \mu_P\left[\left([\hat{M}_1, \hat{M}_2, \cdots, \hat{M}_N], [t_I, t_F]\right) \middle| (s^A, t_I)\right] \quad \text{, --- Eq.(SM-1.6)}$$

where the probability quotient for a (local) indel history is defined as:

$$\mu_P\left[\left([\hat{M}_1, \hat{M}_2, \cdots, \hat{M}_N], [t_I, t_F]\right) \middle| (s^A, t_I)\right] \equiv P\left[\left([\hat{M}_1, \hat{M}_2, \cdots, \hat{M}_N], [t_I, t_F]\right) \middle| (s^A, t_I)\right] \Big/ P\left[\left([\,], [t_I, t_F]\right) \middle| (s^A, t_I)\right] . \quad \text{--- Eq.(SM-1.7)}$$

Similar arguments hold also for the probability, $P\left[\alpha[s_1, s_2, ..., s_{N^X}] \middle| T\right]$, that a MSA ($\alpha[s_1, s_2, ..., s_{N^X}]$) of N^X sequences, $s_1, s_2, ..., s_{N^X}$, results from the evolution along a given phylogenetic tree (T)[22]. Basically in line with the idea in [18,19, 40], we can build up the probability of a MSA, first by multiplying the root state probability and the probabilities of ancestor-descendant PWAs along branches, and second by summing such products over all MSA-consistent ancestral states. The MSA probability thus composed can be expressed as a series:

$$P\left[\alpha[s_1, s_2, ..., s_{N^X}] \middle| T\right] = \sum_{N=N_{\min}}^{\infty} P_{(N)}\left[\alpha[s_1, s_2, ..., s_{N^X}] \middle| T\right]. \quad \text{--- Eq.(SM-1.8)}$$

(It corresponds to Rq.(R1.4).) Here, $N_{\min}$ is the minimum number of indels required for creating the MSA. (For simplicity, we omitted the obvious dependence of $N_{\min}$ on the MSA and the tree.) And $P_{(N)}\left[\alpha[s_1, s_2, ..., s_{N^X}] \,|\, T\right]$ is the portion of the probability contributed from all MSA-consistent N -event indel histories along T. Let $\Psi^{ID}\left[N; \alpha[s_1, s_2, ..., s_{N^X}]; T\right]$ be the set of all such indel histories. Then, we have:

$$P_{(N)}\left[\alpha[s_1, s_2, ..., s_{N^X}] \,|\, T\right] = \sum_{\substack{\left(s^{Root}, \left\{\vec{\hat{M}}(b)\right\}_T\right) \\ \in \Psi^{ID}\left[N;\, \alpha[s_1, s_2, ..., s_{N^X}];\, T\right]}} P\left[\left(s^{Root}, n^{Root}\right)\right] P\left[\left\{\vec{\hat{M}}(b)\right\}_T \,\Big|\, \left(s^{Root}, n^{Root}\right)\right] .$$

--- Eq.(SM-1.9)

Here, $\left(s^{Root}, \left\{\vec{\hat{M}}(b)\right\}_T\right)$ denotes an indel history along T that starts with the sequence state (s^{Root}) at the root node (n^{Root}) and that consists of (mutually interdependent) indel histories ($\vec{\hat{M}}(b)$'s) along branches (b's). ($\vec{\hat{M}}(b)$ will also be denoted as: $\left[\hat{M}_1(b), \cdots, \hat{M}_{N(b)}(b)\right]$.)

$P\left[\left(s^{Root}, n^{Root}\right)\right]$ denotes the (prior) probability that we have s^{Root} at n^{Root}. And $P\left[\left\{\vec{\hat{M}}(b)\right\}_T \,\Big|\, \left(s^{Root}, n^{Root}\right)\right]$ is the probability that all $\vec{\hat{M}}(b)$'s occur, given s^{Root} at n^{Root}. Its specific expression is:

$$P\left[\left\{\vec{\hat{M}}(b)\right\}_T \,\Big|\, \left(s^{Root}, n^{Root}\right)\right] = \left(\prod_{b \in \{b\}_T} P\left[\left(\vec{\hat{M}}(b), b\right) \Big| \left(s^A(b), n^A(b)\right)\right] \right)\Bigg|_{\substack{s(n^{Root}) = s^{Root}, \\ \langle s^D(b)| = \langle s^A(b)| \hat{M}_1(b) \cdots \hat{M}_{N(b)}(b) \\ for\ {}^\forall b \in \{b\}_T}} ,$$

--- Eq.(SM-1.10)

with

$$P\left[\left(\vec{\hat{M}}(b), b\right) \Big| \left(s^A(b), n^A(b)\right)\right]$$
$$\equiv P\left[\left(\left[\hat{M}_1(b), \cdots, \hat{M}_{N(b)}(b)\right], \left[t(n^A(b)),\ t(n^D(b))\right]\right) \Big| \left(s^A(b), t(n^A(b))\right)\right]\Bigg|_{\Theta_{ID}(b)} .$$

--- Eq.(SM-1.11)

Here $\{b\}_T$ is the set of all branches in T. $n^A(b)$ and $n^D(b)$ are the upstream and downstream nodes, respectively, of branch b; $s^A(b)$ and $s^D(b)$ are the sequence states at the respective nodes. And Eq.(SM-1.11) explicitly records the dependence on the (possibly

branch-dependent) model parameter setting ($\Theta_{ID}(b)$). **Eqs.(SM-1.10,11) give essential building blocks for MSA probabilities.**

A MSA-counterpart of a PAS is a gapless column, which indicates that the corresponding site was hit by no indel throughout the evolution along T. (Hereafter, a gapless column in a MSA is also called a "PAS.") Using some PASs, we partition the MSA into local regions, $C_1, C_2, ..., C_{\mathrm{K}_{\max}}$. Meanwhile, there are infinitely many possible root sequence states (s^{Root}'s) consistent with the MSA. Among them, we choose one as the "reference" root state (s_0^{Root}). Then, in addition to the aforementioned conditions (i) and (ii), we impose the following condition.

Condition (iii): the (prior) probability of each root state (s^{Root}) is given by the probability of s_0^{Root} multiplied by the product of factors over the local regions, where each factor depends only on the difference between s^{Root} and s_0^{Root} in a local region.

Under these conditions, the MSA probability is factorized as:

$$P\left[\alpha[s_1, s_2, ..., s_{N^X}] \mid T\right] = P_0\left[s_0^{Root} \mid T\right] \prod_{\mathrm{K}=1}^{\mathrm{K}_{\max}} \tilde{\breve{\mathrm{M}}}_P\left[\alpha[s_1, s_2, ..., s_{N^X}]; s_0^{Root}; C_{\mathrm{K}} \mid T\right]. \text{ --- Eq.(SM-1.12)}$$

(It corresponds to Eq.(R1.5).) Here,

$$\begin{aligned} P_0\left[s_0^{Root} \mid T\right] &\equiv P\left[\left(s_0^{Root}, n^{Root}\right)\right] P\left[\{[\,]\}_T \,\middle|\, \left(s_0^{Root}, n^{Root}\right)\right] \\ &= P\left[\left(s_0^{Root}, n^{Root}\right)\right] \exp\left\{-\sum_{b \in \{b\}_T} \int_{t(n^A(b))}^{t(n^D(b))} d\tau \, R_X^{ID}(s_0^{Root}, \tau)\right\} \end{aligned} \text{ --- Eq.(SM-1.13)}$$

is the probability that the root sequence state is s_0^{Root} and that it was hit by no indel all across T. And $\tilde{\breve{\mathrm{M}}}_P\left[\alpha[s_1, s_2, ..., s_{N^X}]; s_0^{Root}; C_{\mathrm{K}} \mid T\right]$ is the multiplication factor contributed from the local region, C_{K}. As in Eq.(SM-1.8), the multiplication factor also can be expressed as a series:

$$\tilde{\breve{\mathrm{M}}}_P\left[\alpha[s_1, s_2, ..., s_{N^X}]; s_0^{Root}; C_{\mathrm{K}} \mid T\right] = \sum_{N=N_{\min}[C_{\mathrm{K}}]}^{\infty} \breve{\mathrm{M}}_{P(N)}\left[\alpha[s_1, s_2, ..., s_{N^X}]; s_0^{Root}; C_{\mathrm{K}} \mid T\right].$$

--- Eq.(SM-1.14)

(It corresponds to Eq.(R1.6).) Here, $N_{\min}\left[C_{\mathrm{K}}\right]$ is the minimum required number of indels in C_{K}. And the term $\breve{\mathrm{M}}_{P(N)}\left[\alpha[s_1, s_2, ..., s_{N^X}]; s_0^{Root}; C_{\mathrm{K}} \mid T\right]$ is the fraction of the multiplication factor contributed from all local-MSA-consistent N-indel local histories in C_{K}. Letting $\Lambda_{\Psi}^{ID}\left[N; C_{\mathrm{K}}; \alpha[s_1, s_2, ..., s_{N^X}]; T\right]$ be the set of all such local indel histories (accompanied by the root states), the term is expressed as:

$$\breve{\mathrm{M}}_{P(N)}\left[\alpha[s_1, s_2,..., s_{N^X}]; s_0^{Root}; C_{\mathrm{K}} \mid T\right] \equiv \sum_{\substack{\left(s^{Root},\, \left\{\hat{\vec{M}}(b)\right\}_T\right) \\ \in \Lambda_{\Psi}^{ID}\left[N; C_{\mathrm{K}}; \alpha[s_1,s_2,...,s_{N^X}]; T\right]}} \left\{ \begin{array}{l} \mu_P\left[s^{Root}, s_0^{Root}, n^{Root}; C_{\mathrm{K}}\right] \mathrm{M}_P\left[\left\{\hat{\vec{M}}(b)\right\}_T \,\middle|\, \left(s^{Root}, n^{Root}\right)\right] \\ \times \exp\left\{ - \sum_{b \in \{b\}_T} \int_{t(n^A(b))}^{t(n^D(b))} d\tau\, \delta R_X^{ID}(s^A(b), s_0^{Root}, \tau)[C_{\mathrm{K}}] \right\} \end{array} \right\} \Bigg|_{\substack{s(n^{Root})=s^{Root}, \\ \left\langle s^D(b)\right| = \left\langle s^A(b)\right| \hat{M}_1(b) \cdots \hat{M}_{N(b)}(b) \\ for\ \forall b \in \{b\}_T}}$$

. --- Eq.(SM-1.15)

Here, $\delta R_X^{ID}(s^A(b), s_0^{Root}, \tau)[C_{\mathrm{K}}]$ is the difference of the exit rate of $s^A(b)$ from that of s_0^{Root}. And $\mu_P\left[s^{Root}, s_0^{Root}, n^{Root}; C_{\mathrm{K}}\right]$ is the (multiplicative) difference in the (prior) probability at n^{Root} between s^{Root} and s_0^{Root}. Both of them originated from C_{K}. (It should be noted that, in Eq.(SM-1.15), $s^A(b)$ differs from s_0^{Root} only within C_{K}.) And the "raw" factor, $\mathrm{M}_P\left[\left\{\hat{\vec{M}}(b)\right\}_T \,\middle|\, \left(s^{Root}, n^{Root}\right)\right]$, is given as:

$$\mathrm{M}_P\left[\left\{\hat{\vec{M}}(b)\right\}_T \,\middle|\, \left(s^{Root}, n^{Root}\right)\right] \equiv \left(\prod_{b \in \{b\}_T} \mu_P\left[\left(\hat{\vec{M}}(b), b\right) \,\middle|\, \left(s^A(b), n^A(b)\right)\right] \right) \Bigg|_{\substack{s(n^{Root})=s^{Root}, \\ \left\langle s^D(b)\right| = \left\langle s^A(b)\right| \hat{M}_1(b) \cdots \hat{M}_{N(b)}(b) \\ for\ \forall b \in \{b\}_T}} .$$

--- Eq.(SM-1.16)

See subsection 4.2 of [49] for the derivation of almost identical (but slightly different) equations.

SM-2. Analytical expressions of parsimonious and next-parsimonious contributions (1): for local PWAs

In case (i) (Figure S1a), the sequence states could be represented as $s^A = s^D = [L, R]$. In this case, $N_{\min}\left[\gamma_K; \alpha(s^A, s^D)\right] = 0$, and thus there is only one parsimonious indel history, $[\,]$, where no indel event takes place. Therefore, in this case, total parsimonious contribution to the multiplication factor is:

$$\mu_{P(0)}\left[case\ (i)\right] = 1 . \quad \text{--- Eq.(SM-2.1)}$$

In this case, there is no history consisting only of one indel event. And each next-parsimonious history should be a two-event history of the form, $\left[\hat{M}_I(1,l), \hat{M}_D(2,l+1)\right]$ with $l = 1,...,L^{CO}$, where $L^{CO} \equiv \min\{L_I^{CO}, L_D^{CO}\}$. Thus, the total next-parsimonious contribution is:

$$\mu_{P(2)}\left[case\ (i)\right]=\sum_{l=1}^{L^{CO}}\mu_P\left[\left(\left[\hat{M}_I(1,l),\hat{M}_D(2,l+1)\right],[t_I,t_F]\right)\middle|\ (s^A,t_I)\right].\qquad \text{--- Eq.(SM-2.2a)}$$

Let $\langle s[l]|\equiv\langle s^A|\hat{M}_I(1,l)$ be the state resulting from the action of an insertion of l sites on s^A. Then, using Eq.(SM-1.3) and Eq.(SM-1.7), each summand is calculated as:

$$\begin{aligned}&\mu_P\left[\left(\left[\hat{M}_I(1,l),\hat{M}_D(2,l+1)\right],[t_I,t_F]\right)\middle|\ (s^A,t_I)\right]\\&=\int_{t_I}^{t_F}dt\int_t^{t_F}dt'\left[\begin{array}{l}r_I(1,l;s^A,t)\,r_D(2,l+1;s[l],t')\\ \times\exp\left\{-\int_{t_I}^{t}d\tau\,\delta R_X^{ID}(s^A,s^A,\tau)-\int_t^{t'}d\tau\,\delta R_X^{ID}(s[l],s^A,\tau)-\int_{t'}^{t_F}d\tau\,\delta R_X^{ID}(s^D,s^A,\tau)\right\}\end{array}\right]\\&=\int_{t_I}^{t_F}dt\int_t^{t_F}dt'\,r_I(1,l;s^A,t)\,r_D(2,l+1;s[l],t')\exp\left\{-\int_t^{t'}d\tau\,\delta R_X^{ID}(s[l],s^A,\tau)\right\}.\end{aligned}$$

--- Eq.(SM-2.2b)

Here, $\delta R_X^{ID}(s,s',t)\equiv R_X^{ID}(s,t)-R_X^{ID}(s',t)$ is the increment of the exit rate. The second equation of Eq.(SM-2.2b) follows from $\delta R_X^{ID}(s^A,s^A,\tau)=\delta R_X^{ID}(s^D,s^A,\tau)=0$. We could at least numerically calculate Eq.(SM-2.2b) once the specific functional forms of the indel rates and the exit rates are given. For example, in a space-time-homogeneous model, like Dawg's indel model [32] (see Eqs.(R1.7,8,9) in the main text), we have $\delta R_X^{ID}(s[l],s^A,\tau)=(\lambda_I+\lambda_D)l$, and Eq.(SM-2.2b) is calculated as:

$$\begin{aligned}&\mu_P\left[\left(\left[\hat{M}_I(1,l),\hat{M}_D(2,l+1)\right],[t_I,t_F]\right)\middle|\ (s^A,t_I)\right]\\&=\lambda_I f_I(l)\,\lambda_D f_D(l)\,\frac{\exp\left(-(\lambda_I+\lambda_D)l(t_F-t_I)\right)-1+(\lambda_I+\lambda_D)l(t_F-t_I)}{\left((\lambda_I+\lambda_D)l\right)^2}.\qquad\text{--- Eq.(SM-2.2b')}\end{aligned}$$

Eq.(SM-2.2b') (or Eq.(SM-2.2b) itself) indicates that

$\mu_P\left[\left(\left[\hat{M}_I(1,l),\hat{M}_D(2,l+1)\right],[t_I,t_F]\right)\middle|\ (s^A,t_I)\right]<\frac{1}{2}\lambda_I f_I(l)\,\lambda_D f_D(l)\,(t_F-t_I)^2$ for each $l=1,...,L^{CO}$.

Applying this inequality to Eq.(SM-2.2a) and using another inequality,

$\sum_{l=1}^{L^{CO}}f_I(l)\,f_D(l)\le\sum_{l=1}^{L_I^{CO}}f_I(l)\sum_{l=1}^{L_D^{CO}}f_D(l)=1$, we have:

$$\mu_{P(2)}\left[case\ (i)\right]<\tfrac{1}{2}\lambda_I\,\lambda_D\,(t_F-t_I)^2.\quad\text{--- Eq.(SM-2.3)}$$

Empirically, the rate of indels ($\lambda_I+\lambda_D$) is estimated to be at most on the order of 1/10 of the substitution rate [24,35]. Eq.(SM-2.3) indicates that, in case (i), even if the elapsed time (t_F-t_I) is such that the substitution process is nearly saturated (*e.g.*, $\lambda_S(t_F-t_I)\approx 4$, where λ_S is the total substitution rate per site), Eq.(SM-2.2a) is at most on the order of 1/10 of Eq.(SM-2.1). Thus, in case (i), we expect that the parsimonious contribution should approximate the entire multiplication factor (Eq.(R1.3)) very well, as far as a single inter-PAS

position is concerned. [NOTE: Incidentally, for a gapless PWA segment consisting of L^P (> 2) PASs, the multiplication factor is formally given between Eq.(1.2.3) and Eq.(1.2.4) in [43].]

In case (ii) (Figure S1b), we assume that the ancestral state has ΔL^A sites in between the flanking PASs. Thus, the ancestral and descendant states could be represented as $s^A = \left[L, 1, ..., \Delta L^A, R\right]$ and $s^D = \left[L, R\right]$, respectively. As long as $\Delta L^A \le L_D^{CO}$,

$N_{\min}\left[case\,(i)\right] = 1$, and there is only one parsimonious indel history, $\left[\hat{M}_D(2, \Delta L^A + 1)\right]$, which consists of a single event that deletes the ancestral sites in between the PASs (Figure S2a). Therefore, the contribution by the parsimonious indel history is:

$$\mu_{P(1)}\left[case\,(ii);\, \Delta L^A\right] = \int_{t_I}^{t_F} dt\; r_D(2, \Delta L^A + 1;\, s^A, t) \exp\left\{-\int_{t_I}^{t} d\tau\; \delta R_X^{ID}(s^A, s^A, t) - \int_{t}^{t_F} d\tau\; \delta R_X^{ID}(s^D, s^A, t)\right\}$$
$$= \int_{t_I}^{t_F} dt\; r_D(2, \Delta L^A + 1;\, s^A, t) \exp\left\{-\int_{t}^{t_F} d\tau\; \delta R_X^{ID}(s^D, s^A, t)\right\}.$$

--- Eq.(SM-2.4)

Each next-parsimonious indel history is composed of two indel events. There are two types.

(a) Two successive deletions, $\left[\hat{M}_D(x, x+l-1), \hat{M}_D(2, \Delta L^A - l + 1)\right]$ with $l = 1, ..., \Delta L^A - 1$ and $x = 2, ..., \Delta L^A - l + 2$ (*e.g.*, Figure S2b). And (b) an insertion followed by a deletion, $\left[\hat{M}_I(x, l), \hat{M}_D(2, \Delta L^A + l + 1)\right]$ with $l = 1, ..., \min\{L_I^{CO}, L_D^{CO} - \Delta L^A\}$ and $x = 1, ..., \Delta L^A + 1$ (*e.g.*, Figure S2c). Thus, in this case, the total next-parsimonious contribution is given by:

$$\mu_{P(2)}\left[case\,(ii);\, \Delta L^A\right] = \mu_P\left[(a);\, \Delta L^A\right] + \mu_P\left[(b);\, \Delta L^A\right] \quad . \quad \text{--- Eq.(SM-2.5a)}$$

Here,

$$\mu_P\left[(a);\, \Delta L^A\right] \equiv \sum_{l=1}^{\Delta L^A - 1} \sum_{x=2}^{\Delta L^A - l + 2} \mu_P\left[\left(\left[\hat{M}_D(x, x+l-1), \hat{M}_D(2, \Delta L^A - l + 1)\right], [t_I, t_F]\right) \Big|\, (s^A, t_I)\right]$$

--- Eq.(SM-2.5b)

is the sum of contributions from the histories of type (a). And

$$\mu_P\left[(b);\, \Delta L^A\right] \equiv \sum_{l=1}^{\min\{L_I^{CO}, L_D^{CO} - \Delta L^A\}} \sum_{x=1}^{\Delta L^A + 1} \mu_P\left[\left(\left[\hat{M}_I(x, l), \hat{M}_D(2, \Delta L^A + l + 1)\right], [t_I, t_F]\right) \Big|\, (s^A, t_I)\right]$$

--- Eq.(SM-2.5c)

is the sum of contributions from the histories of type (b). Let

$\left\langle s^A \cdot [x, -l]\right| \equiv \left\langle s^A \right| \hat{M}_D(x, x+l-1)$ be the intermediate state in each type (a) history. Then, the

history's contribution is calculated as:

$$\mu_P\left[\left(\left[\hat{M}_D(x,x+l-1),\hat{M}_D(2,\Delta L^A-l+1)\right],[t_I,t_F]\right)\middle|\,(s^A,t_I)\right]$$
$$=\int_{t_I}^{t_F}dt\int_{t}^{t_F}dt'\begin{bmatrix}r_D(x,x+l-1;s^A,t)\,r_D(2,\Delta L^A-l+1;s^A\cdot[x,-l],t')\\ \times\exp\left\{-\int_t^{t'}d\tau\,\delta R_X^{ID}(s^A\cdot[x,-l],s^A,\tau)-\int_{t'}^{t_F}d\tau\,\delta R_X^{ID}(s^D,s^A,\tau)\right\}\end{bmatrix}.$$

--- Eq.(SM-2.5d)

Similarly, let $\langle s^A\cdot[x,+l]|\equiv\langle s^A|\hat{M}_I(x,l)$ be the intermediate state in each type (b) history.

Then, the history's contribution is calculated as:

$$\mu_P\left[\left(\left[\hat{M}_I(x,l),\hat{M}_D(2,\Delta L^A+l+1)\right],[t_I,t_F]\right)\middle|\,(s^A,t_I)\right]$$
$$=\int_{t_I}^{t_F}dt\int_{t}^{t_F}dt'\begin{bmatrix}r_I(x,l;s^A,t)\,r_D(2,\Delta L^A+l+1;s^A\cdot[x,+l],t')\\ \times\exp\left\{-\int_t^{t'}d\tau\,\delta R_X^{ID}(s^A\cdot[x,+l],s^A,\tau)-\int_{t'}^{t_F}d\tau\,\delta R_X^{ID}(s^D,s^A,\tau)\right\}\end{bmatrix}.$$

--- Eq.(SM-2.5e)

Eq.(SM-2.4) and Eqs.(SM-2.5a-e) can indeed be calculated at least numerically, given concrete indel rates and exit rates. For example, under Dawg's indel model (Eqs.(R1.7,8,9)), we have $\delta R_X^{ID}(s^D,s^A,\tau)=-(\lambda_I+\lambda_D)\Delta L^A$, and Eq.(SM-2.4) becomes:

$$\mu_{P(1)}\left[case\ (ii);\,\Delta L^A\right]=\lambda_D f_D(\Delta L^A)\,\frac{\exp\left((\lambda_I+\lambda_D)\Delta L^A(t_F-t_I)\right)-1}{(\lambda_I+\lambda_D)\Delta L^A}\quad\text{. --- Eq.(SM-2.4')}$$

Similarly, using $\delta R_X^{ID}(s^A\cdot[x,-l],s^A,\tau)=-(\lambda_I+\lambda_D)\,l$ and $\delta R_X^{ID}(s^A\cdot[x,+l],s^A,\tau)=+(\lambda_I+\lambda_D)\,l$, Eqs.(SM-2.5d,e) under Dawg's model are calculated, respectively, as:

$$\mu_P\left[\left(\left[\hat{M}_D(x,x+l-1),\hat{M}_D(2,\Delta L^A-l+1)\right],[t_I,t_F]\right)\middle|\,(s^A,t_I)\right]$$
$$=\frac{\lambda_D f_D(l)\,\lambda_D f_D(\Delta L^A-l)}{(\lambda_I+\lambda_D)(\Delta L^A-l)}\left[\frac{e^{(\lambda_I+\lambda_D)\Delta L^A(t_F-t_I)}-1}{(\lambda_I+\lambda_D)\Delta L^A}-\frac{e^{(\lambda_I+\lambda_D)l(t_F-t_I)}-1}{(\lambda_I+\lambda_D)l}\right],\quad\text{--- Eq.(SM-2.5d')}$$

$$\mu_P\left[\left(\left[\hat{M}_I(x,l),\hat{M}_D(2,\Delta L^A+l+1)\right],[t_I,t_F]\right)\middle|\,(s^A,t_I)\right]$$
$$=\frac{\lambda_I f_I(l)\,\lambda_D f_D(\Delta L^A+l)}{(\lambda_I+\lambda_D)(\Delta L^A+l)}\left[\frac{e^{(\lambda_I+\lambda_D)\Delta L^A(t_F-t_I)}-1}{(\lambda_I+\lambda_D)\Delta L^A}-\frac{1-e^{-(\lambda_I+\lambda_D)l(t_F-t_I)}}{(\lambda_I+\lambda_D)l}\right].\quad\text{--- Eq.(SM-2.5e')}$$

Substituting Eqs.(SM-2.5d',e') into Eqs.(SM-2.5a,b,c), we can concretely calculate the total next-parsimonious contribution. Figure S4 shows the ratio of Eq.(SM-2.5a) to Eq.(SM-2.4), as a function of ΔL^A (abscissa) and the expected number of indels per site ($(\lambda_I+\lambda_D)(t_F-t_I)$, different curves). Here, the exact overall indel rate ($\lambda_I+\lambda_D$) does not matter, because the probabilities are invariant under the simultaneous rescaling of the rate and the time interval

$(t_F - t_I)$ that keeps $(\lambda_I + \lambda_D)(t_F - t_I)$ unchanged. As indicated by Figure S4, each curve for a fixed $(\lambda_I + \lambda_D)(t_F - t_I)$ reaches an asymptotic value slightly above 1 (unity) when ΔL^A is sufficiently large. Thus, to define a threshold within which the parsimonious histories alone are likely to give a decent approximation of the multiplication factor, it is risky to use the point at which the ratio is 1 (unity). Here, we tentatively define the threshold, $(\Delta L)_{0.5}^{(NP)}$, as the value of ΔL^A at which the ratio is 0.5. With this definition, $(\Delta L)_{0.5}^{(NP)}$ is around 128, 31, 12, 6 and 3 when $(\lambda_I + \lambda_D)(t_F - t_I)$ is 0.01, 0.04, 0.1, 0.2 and 0.4, respectively (Table S1). Hence, we have a rough inversely proportional relationship: $(\Delta L)_{0.5}^{(NP)} \approx 1.2/[(\lambda_I + \lambda_D)(t_F - t_I)]$, under the parameter setting used here.

In case (iii) (Figure S1c), we assume that the descendant state has ΔL^D sites in between the flanking PASs. Thus, the ancestral and descendant states could be represented as $s^A = [L, R]$ and $s^D = [L, \upsilon_1^D, ..., \upsilon_{\Delta L^D}^D, R]$, respectively. The ancestries satisfy $\upsilon_i^D \notin \{L, R\}$ for every $i = 1, ..., \Delta L^D$, and $\upsilon_i^D \neq \upsilon_j^D$ for every pair (i, j) with $i \neq j$, and their details depend on the responsible indel history. (Actually, such details don't matter in the state space S^{II}, as explained in [22].) As long as $\Delta L^D \leq L_I^{CO}$, $N_{\min}[case\,(iii)] = 1$, and there is only one parsimonious indel history, $[\hat{M}_I(1, \Delta L^D)]$. The history consists of a single event that inserts the descendant sites in between the PASs. As in case (ii), each next-parsimonious indel history is composed of two indel events, and classified into two types. (c) Two successive insertions, $[\hat{M}_I(1, \Delta L^D - l), \hat{M}_I(x, l)]$ with $l = 1, ..., \Delta L^D - 1$ and $x = 1, ..., \Delta L^D - l + 1$. And (d) an insertion followed by a deletion, $[\hat{M}_I(1, \Delta L^D + l), \hat{M}_D(x, x + l - 1)]$ with $l = 1, ..., \min\{L_D^{CO}, L_I^{CO} - \Delta L^D\}$ and $x = 2, ..., \Delta L^D + 2$. The total parsimonious contribution ($\mu_{P(1)}[case\,(iii); \Delta L^D]$) and the total next-parsimonious contribution ($\mu_{P(2)}[case\,(iii); \Delta L^D]$) can be calculated as in case (ii). And their calculations are detailed in Appendix A1.1 of [43]. If calculated under the same setting as used for Figure S4 and with the same value of $(\lambda_I + \lambda_D)(t_F - t_I)$, their ratio with $\Delta L^D = L$ is identical to that in case (ii) with $\Delta L^A = L$, because of the symmetry between the probabilities under the time reversal. Thus, the same conclusions as in case (ii) can be drawn from Figure S4 also in this case.

In case (iv) (Figuer S1d), we assume that the ancestral and the descendant states have ΔL^A and ΔL^D sites, respectively, in between the flanking PASs. Thus, the ancestral

and descendant states could be represented as $s^A = \left[L, 1, ..., \Delta L^A, R\right]$ and

$s^D = \left[L, \upsilon_1^D, ..., \upsilon_{\Delta L^D}^D, R\right]$, respectively. Here, the descendant ancestries satisfy

$\upsilon_i^D \notin \{L, 1, ..., \Delta L^A, R\}$ for every $i = 1, ..., \Delta L^D$, and $\upsilon_i^D \neq \upsilon_j^D$ for every pair (i, j) with $i \neq j$, and their details depend on the responsible indel history. (Again, the details don't matter in S^{II}.) As long as $\Delta L^A \leq L_D^{CO}$ and $\Delta L^D \leq L_I^{CO}$, $N_{\min}\left[case\,(iv)\right] = 2$. In this case, there are three types of parsimonious indel histories (Figure S3). (e) The deletion of the ancestral sites followed by an insertion of ΔL^D sites, $\left[\hat{M}_D(2, \Delta L^A + 1), \hat{M}_I(1, \Delta L^D)\right]$ (Figure S3a). (f) An insertion immediately on the right of the ancestral sites to be deleted, followed by the deletion, $\left[\hat{M}_I(\Delta L^A + 1, \Delta L^D + l), \hat{M}_D(2, \Delta L^A + l + 1)\right]$ with $l = 0, ..., \min\{L_I^{CO} - \Delta L^D, L_D^{CO} - \Delta L^A\}$ (*e.g*, Figure S3, panels b and d). And (g) an insertion immediately on the left of the ancestral sites to be deleted, followed by the deletion, $\left[\hat{M}_I(1, \Delta L^D + l), \hat{M}_D(\Delta L^D + 2, \Delta L^A + \Delta L^D + l + 1)\right]$ also with $l = 0, ..., \min\{L_I^{CO} - \Delta L^D, L_D^{CO} - \Delta L^A\}$ (*e.g.*, Figure S3, panels c and e). In this case, each next-parsimonious indel history is composed of three indel events, and classified into one of 6 broad types: (h) two successive deletions followed by an insertion; (i) a deletion, followed by an insertion, followed by a deletion; (j) an insertion followed by two successive deletions; (k) a deletion followed by two successive insertions; (l) an insertion, followed by a deletion, followed by an insertion; and (m) two successive insertions followed by a deletion. And these six broad types can be further sub-classified into 24 sub-types, as described in Appendix A1.2 of [43]. There, the calculations of the total parsimonious contribution ($\mu_{P(2)}\left[case\,(iv); \Delta L^A, \Delta L^D\right]$) and the total next-parsimonious contribution ($\mu_{P(3)}\left[case\,(iv); \Delta L^A, \Delta L^D\right]$) are also detailed.

SM-3. Integral equation system for "exact" multiplication factors for local PWAs

In this section, we derive a system of integral equations to give practically exact solutions (or "exact" solutions, for short) of the multiplication factors for cases (i) and (ii). (Another system of integral equations, which gives "exact" multiplication factors for cases (i) and (iii), is derived in Appendix A1.3 of [43].)

Here, we assume that the indel rates are locally homogeneous, which means that the

rates do not depend on the exact positions that the indels hit, *as long as* they are confined in the region that accommodates the local history. Thus, we assume that the indel rate is *locally* homogeneous and the exit rate is *locally* an affine function of the (local) sequence length, but that they may be non-homogeneous *globally*. (In terms of equations, we *locally* assume Eqs.(5.1.1a,b) of [49] for the indel rates and Eq.(5.2.4) of [49] for the *local* exit rate, but we assume something like Eqs.(5.3.2a,b,c) of [49] for the *global* exit rate.) We are now considering only cases (i) and (ii), in which (local) ancestral and descendant states should be $s^A = \left[L, 1, ..., \Delta L^A, R\right]$ and $s^D = \left[L, R\right]$, respectively, with $\Delta L^A = 0,1,2,...$. Because of the local homogeneity, the exit rate $R_X^{ID}(s,t)$ of a state $s\,(\in S)$ in this context depends only on the (local) sequence length, $L(s) = 2 + \Delta L(s)$. Thus, $\Delta L(s)$ adequately represents the local sequence state s, and we let $R_X^{ID}(\Delta L(s), t)$ denote its (local) exit rate. The starting point of the equation system is the fundamental integral equation (Eq.(R4.5) of [22]) for the finite-time transition operator, $\hat{P}^{ID}(t_I, t_F)$. We sandwich the fundamental integral equation with $\left\langle s^A \right|$ and $\left| s^D \right\rangle$, and expand the instantaneous mutation operator, $\hat{Q}_M^{ID}(t) \equiv \hat{Q}_M^I(t) + \hat{Q}_M^D(t)$, using the components' definitions (*i.e.*, Eqs.(R3.12,13) of [22]). Because we know that no indels struck the flanking PASs (with ancestries L and R), we can ignore the effects of indels that hit the PASs. And, because we are now focusing on the local alignment, we will also ignore the indels completely outside of the region delimited by the PASs. Thus, we have:

$$\left\langle s^A \middle| \hat{P}^{ID}(t_I, t_F) \middle| s^D \right\rangle = \left\langle s^A \middle| s^D \right\rangle \exp\left\{-\int_{t_I}^{t_F} dt\, R_X^{ID}\left(\Delta L^A, t\right)\right\}$$
$$+ \sum_{x=1}^{\Delta L^A+1} \sum_{l=1}^{L_I^{CO}} \int_{t_I}^{t_F} dt \left[\exp\left\{-\int_{t_I}^{t} d\tau\, R_X^{ID}\left(\Delta L^A, \tau\right)\right\} g_I(l,t) \left\langle s^A \middle| \hat{M}_I(x,l)\, \hat{P}^{ID}(t, t_F) \middle| s^D \right\rangle\right]$$
$$+ \sum_{l=1}^{\min\{\Delta L^A, L_D^{CO}\}} \sum_{x=2}^{\Delta L^A - l + 2} \int_{t_I}^{t_F} dt \left[\exp\left\{-\int_{t_I}^{t} d\tau\, R_X^{ID}\left(\Delta L^A, \tau\right)\right\} g_D(l,t) \left\langle s^A \middle| \hat{M}_D(x, x+l-1)\, \hat{P}^{ID}(t, t_F) \middle| s^D \right\rangle\right].$$

--- Eq.(SM-3.1)

(Here, $g_I(l,t)$ is the rate of an insertion of length l, and $g_D(l,t)$ is the rate of a deletion of length l.) In the present setting, the number of sites between the PASs, $\Delta L(s)$, uniquely determines the local sequence state s (or, more precisely, the equivalence class of sequence states in the sense that they give the same probability of the finite-time transition to $\left\langle s^D \right| = \left\langle 0 \right|$). Thus, we let the local states denoted as $\left\langle s^A \right| = \left\langle \Delta L^A \right|$, $\left\langle s^D \right| = \left\langle 0 \right|$,

$\left\langle s^A \right| \hat{M}_I(x,l) = \left\langle \Delta L^A + l \right|$, and $\left\langle s^A \right| \hat{M}_D(x, x+l-1) = \left\langle \Delta L^A - l \right|$. We also introduce the notation,

$P^{ID}\left(\Delta L \mapsto \Delta L'; [t, t']\right) \equiv \left\langle \Delta L \right| \hat{P}^{ID}(t, t') \left| \Delta L' \right\rangle$. Then, taking advantage of the independence of the indel rates, exit rates and $P^{ID}\left(\Delta L \mapsto \Delta L'; [t, t']\right)$ on the position of each indel (x), we have:

$$P^{ID}\left(\Delta L^A \mapsto 0; [t_I, t_F]\right) = \delta(\Delta L^A, 0) \exp\left\{-\int_{t_I}^{t_F} dt\, R_X^{ID}\left(\Delta L^A = 0, t\right)\right\}$$
$$+ (\Delta L^A + 1) \sum_{l=1}^{L_I^{CO}} \int_{t_I}^{t_F} dt \left[\exp\left\{-\int_{t_I}^{t} d\tau\, R_X^{ID}\left(\Delta L^A, \tau\right)\right\} g_I(l,t)\, P^{ID}\left(\Delta L^A + l \mapsto 0; [t, t_F]\right)\right]$$
$$+ \sum_{l=1}^{\min\{\Delta L^A, L_D^{CO}\}} (\Delta L^A - l + 1) \int_{t_I}^{t_F} dt \left[\exp\left\{-\int_{t_I}^{t} d\tau\, R_X^{ID}\left(\Delta L^A, \tau\right)\right\} g_D(l,t)\, P^{ID}\left(\Delta L^A - l \mapsto 0; [t, t_F]\right)\right].$$

--- Eq.(SM-3.2)

Here $\delta(\Delta L, \Delta L')$ is Kronecker's delta, which equals 1 if $\Delta L = \Delta L'$, and 0 if $\Delta L \neq \Delta L'$. Eq.(SM-3.2) gives the desired system of integral equations for the "exact" probabilities, $P^{ID}\left(\Delta L^A \mapsto 0; [t_I, t_F]\right)$, with non-negative integers $\Delta L^A = 0,1,2,\ldots$. This equation holds for every non-negative integer ΔL^A and even if we replace the initial time t_I with any time in the closed interval $[t_I, t_F]$. Thus, the equations can be solved iteratively, starting with the "zero-event approximation" of the probability,

$P_{\langle 0 \rangle}^{ID}\left(\Delta L^A \mapsto 0; [t, t_F]\right) = \delta(\Delta L^A, 0) \exp\left\{-\int_{t}^{t_F} d\tau\, R_X^{ID}\left(\Delta L^A = 0, \tau\right)\right\}$, and calculating the approximation at the n_S th step, $P_{\langle n_S \rangle}^{ID}\left(\Delta L^A \mapsto 0; [t, t_F]\right)$, from the approximation at the previous step via the recursion relation:

$$P_{\langle n_S \rangle}^{ID}\left(\Delta L^A \mapsto 0; [t, t_F]\right) = \delta(\Delta L^A, 0) \exp\left\{-\int_{t}^{t_F} d\tau\, R_X^{ID}\left(\Delta L^A = 0, \tau\right)\right\}$$
$$+ (\Delta L^A + 1) \sum_{l=1}^{L_I^{CO}} \int_{t}^{t_F} dt' \left[\exp\left\{-\int_{t}^{t'} d\tau\, R_X^{ID}\left(\Delta L^A, \tau\right)\right\} g_I(l,t')\, P_{\langle n_S - 1 \rangle}^{ID}\left(\Delta L^A + l \mapsto 0; [t', t_F]\right)\right]$$
$$+ \sum_{l=1}^{\min\{\Delta L^A, L_D^{CO}\}} (\Delta L^A - l + 1) \int_{t}^{t_F} dt' \left[\exp\left\{-\int_{t}^{t'} d\tau\, R_X^{ID}\left(\Delta L^A, \tau\right)\right\} g_D(l,t')\, P_{\langle n_S - 1 \rangle}^{ID}\left(\Delta L^A - l \mapsto 0; [t', t_F]\right)\right].$$

--- Eq.(SM-3.2')

If N_{ID} iteration steps are performed, the resulting probability, $P_{\langle N_{ID} \rangle}^{ID}\left(\Delta L^A \mapsto 0; [t_I, t_F]\right)$ (= $\sum_{N=0}^{N_{ID}} \left\langle \Delta L^A \right| \hat{P}_{(N)}^{ID}(t_I, t_F) \left| 0 \right\rangle$, where $\hat{P}_{(N)}^{ID}(t_I, t_F)$ is the collection of terms with N indels each), is

the summation of the probabilities over all possibly responsible local histories consisting of up to (and including) N_{ID} indel events. After the iteration is finished, the multiplication factor will be obtained by following its definition (*i.e.*, Eq.(R6.3) in [22]). We have:

$$\tilde{\mu}_P^{\langle N_{ID}\rangle}\left[cases\,(i)\,\&\,(ii);\,\Delta L^A\right] \equiv \sum_{N=0}^{N_{ID}} \mu_{P(N)}\left[cases\,(i)\,\&\,(ii);\,\Delta L^A\right]$$
$$= P_{\langle N_{ID}\rangle}^{ID}\left(\Delta L^A \mapsto 0;[t_I,t_F]\right) \Big/ P\left[\left([\,],[t_I,t_F]\right) \middle| \,(s^A,t_I)\right] \qquad \text{--- Eq.(SM-3.3)}$$
$$= \exp\left\{+\int_{t_I}^{t_F} dt\, R_X^{ID}\left(\Delta L^A, t\right)\right\} P_{\langle N_{ID}\rangle}^{ID}\left(\Delta L^A \mapsto 0;[t_I,t_F]\right).$$

The accuracy of the numerical solutions will depend on how finely we partition the time interval $[t_I,t_F]$. If the interval is partitioned into N_P equal-sized sub-intervals, we could in principle achieve an accuracy of $O\left((N_P)^{-4}\right)$ under Simpson's rule (*e.g.*, [63]). However, as the number of sub-intervals increases, it would take longer to complete the calculation. A naïve implementation of the aforementioned numerical iteration would have the time complexity of $O\left(N_{ID}(L^{CO})^2(N_P)^2\right)$ and the space complexity of $O\left(L^{CO}N_P\right)$, when we want the probabilities taking account of up to N_{ID} indels per local region, with $\Delta L^A = 0,1,2,\ldots,\Delta L^A_{\max}(\leq L^{CO})$. Here we set $L_I^{CO} = L_D^{CO} \equiv L^{CO}$. This becomes impractically slow when either L^{CO} or N_P is large, *e.g.*, around 1000 or greater. It is likely that N_P does not have to be this large, as it would be usually enough to set N_P around 100 or smaller. However, L^{CO} will often be around 1000 or greater, indeed making the naïve algorithm too slow to be practical. Fortunately, we can avoid this problem by rewriting the recursion equation, Eq.(SM-3.2'), as:

$$P_{\langle n_S\rangle}^{ID}\left(\Delta L^A \mapsto 0;[t,t_F]\right) = \delta(\Delta L^A,0)\exp\left\{-\int_t^{t_F} d\tau\, R_X^{ID}\left(\Delta L^A=0,\tau\right)\right\}$$
$$+\int_t^{t_F} dt'\left[\exp\left\{-\int_t^{t'} d\tau\, R_X^{ID}\left(\Delta L^A,\tau\right)\right\}\Phi_{\langle n_S\rangle}^{ID}\left(\Delta L^A \mapsto 0;[t',t_F]\right)\right]. \qquad \text{--- Eq.(SM-3.4a)}$$

Here, the "auxiliary function," $\Phi_{\langle n_S\rangle}^{ID}\left(\Delta L^A \mapsto 0;[t',t_F]\right)$, is given by:

$$\Phi_{\langle n_S\rangle}^{ID}\left(\Delta L^A \mapsto 0;[t,t_F]\right) \equiv (\Delta L^A+1)\sum_{l=1}^{L_I^{CO}}\left[g_I(l,t)\,P_{\langle n_S-1\rangle}^{ID}\left(\Delta L^A+l \mapsto 0;[t,t_F]\right)\right]$$
$$+\sum_{l=1}^{\min\{\Delta L^A,L_D^{CO}\}}\left[(\Delta L^A-l+1)g_D(l,t)\,P_{\langle n_S-1\rangle}^{ID}\left(\Delta L^A-l \mapsto 0;[t,t_F]\right)\right]. \qquad \text{--- Eq.(SM-3.4b)}$$

Consider the following "two-sub-step" algorithm. In the first sub-step (in each iteration step), it calculates $\Phi_{\langle n_S\rangle}^{ID}\left(\Delta L^A \mapsto 0;[t,t_F]\right)$'s via Eq.(SM-3.4b) and stores them for all

$\Delta L^A = 0,1,2,..., L^{CO}$ at all time points, $t = t_I + i\,\frac{t_F - t_I}{N_P}$ with $i = 0,1,..., N_P$. And, in the second sub-step, it uses them to calculate the probabilities $P^{ID}_{\langle n_S \rangle}\left(\Delta L^A \mapsto 0; [t, t_F]\right)$ via Eq.(SM-3.4a) for the same sets of values of ΔL^A and t. This algorithm can reduce the time-complexity to $O\left(N_{ID} L^{CO}(L^{CO} + N_P) N_P\right)$ while keeping the space complexity to be $O\left(L^{CO} N_P\right)$. This algorithm does finish in a practical amount of time (typically on the order of an hour or shorter when implemented in Perl). But it may still be too slow to perform each time we evaluate the probability of a local MSA. Good news is that a single run of the iteration algorithm *inevitably* calculates the probabilities for all $\Delta L^A = 0,1,2,..., \Delta L^A_{\max} (\le L^{CO})$ at all temporal partitioning points, $t = t_I + i\,\frac{t_F - t_I}{N_P}$ ($i = 1,..., N_P - 1$), as well as at $t = t_I$ (originally desired) and $t = t_F$ (trivial). Thus, once we calculate the probabilities with a fixed set of model parameters, we could use them to calculate the probabilities of various alignments (under various phylogenetic trees), as long as the model parameters remain unchanged. In any case, the time and space complexities might be further reduced without considerably compromising the accuracy by a clever beforehand discarding of terms unlikely to contribute significantly to the final probabilities. (And the computation will speed up at least 10-fold if implemented, *e.g*, in C.) Figure 2 shows the ratios of the multiplication factors, Eq.(SM-3.3) at $N_{ID} = 1, 2, 5, 10, 20$ iteration steps, to that at $N_{ID} = 200$ steps. When $N_{ID} \ge 2$, we actually started from $N_{ID} = 2$, at which the probabilities ($P^{ID}_{\langle 2 \rangle}\left(\Delta L^A \mapsto 0; [t, t_F]\right)$'s) were calculated as explained in section SM-2, instead of from $N_{ID} = 0$ as mentioned above, in order to enhance the accuracy of the approximation. As indicated by Figure 2, the accuracy of the probabilities improves in a step-wise manner as the number of iterations increases.

Following the similar procedures, this time starting from the integral equation, Eq.(R4.4) in [22], we can also derive a system of integral equations for the multiplication factors for cases (i) and (iii), as described in Appendix A1.3 of [43]. Thanks to the symmetry between the probabilities under the time reversal, Figure 2 can also be interpreted as the results of numerical calculations of this equation system under the same setting.

SM-3.1. Fitting power-law to finite-time transition probabilities

To examine how well the power-law function fits the finite-time transition probabilities ($P^{ID}\left(0 \mapsto \Delta L^D; [t_I, t]\right)$ with $t \in [t_I, t_F]$) of case (iii) local PWAs, we performed the correlation and linear regression analyses on the log-log plots between ΔL^D and

$\tilde{\mu}_P^{\langle N_{ID}=200\rangle}\left[case\,(iii);\Delta L^D;[t_I,t]\right]$. We used $\log(\Delta L^D)$ as the independent variable (X) and

$\log\left(\tilde{\mu}_P^{\langle N_{ID}=200\rangle}\left[case\,(iii);\Delta L^D;[t_I,t]\right]\right)$ as the dependent variable (Y). We weighted each point

with ΔL^D (=1,2,…,300), by $\tilde{\mu}_P^{\langle N_{ID}=200\rangle}\left[case\,(iii);\Delta L^D;[t_I,t]\right]$, in order to mimic the population of the observed points in a *virtual* analysis based on these finite-time probabilities. These analyses were performed with $\lambda_I:\lambda_D$ = $1:1$, $1:3$ and $3:1$, and with other parameter setting described in section M1 of Methods.

SM-4. Analytical expressions of parsimonious and next-parsimonious contributions (2): for local MSAs

Compared to contributions to local PWAs, those to local MSAs are much more complex. In this section, we consider some simple but common patterns, under a tree T with three OTUs, corresponding to the external nodes, n_1, n_2 and n_3. Here, we regard its single internal node as the root node n^{Root} for simplicity (panel a of Figure 4). Let b_m ($m=1,2,3$) be the branch that connects the nodes n^{Root} and n_m. Let $s_m \in S^{II}$ ($m=1,2,3$) be the (local) sequence state at node n_m. Then, we consider the gap-configurations (or, more precisely, the homology structures) of the MSAs of the three sequences, $\alpha[s_1,s_2,s_3]$, as well as the consistent sequence states $s^{Root} \in S^{II}$ at the root node n^{Root}. As in the previous sections, we focus on the portion of MSAs delimited by a pair of PASs, whose ancestries are denoted as L and R. Here we consider four typical situations (see Figure 4; see also Subsection 3.4 of [49] for complexities concerning this issue). (I) None of $\{s_1,s_2,s_3\}$ has any site in between the PASs (Figure 4, panel b). (II) s_1 and s_2 share the identical set of sites in between the PASs, but s_3 has no site in between (panel c). (III) s_1 has a set of sites in between the PASs, but neither s_2 nor s_3 has even a single site in between (panel d). And (IV) s_1 has a set of sites in between the PASs, but s_3 has no site in between, and s_2 lacks a run of some, but not all, of contiguous sites of s_1 in between the PASs (panel e). These situations are not restricted to the 3-OTU trees but widely applicable to each portion surrounding a trivalent node of any tree topology, although they never exhaust all gap configurations. The time at n^{Root} will be represented as t_I, and the time at node n_m will be represented as $t_{F:m}$. The indel parameters along branch b_m will be indicated by the subscript "$:m$."

Case (I) is represented by the external sequence states $s_1=s_2=s_3=[L,R]$ (Figure 4b). In this case, we have $N_{\min}[case\,(I)]=0$. And the set of fewest-indel local histories, $\Lambda_\Psi^{ID}[N_K=0;C_K;\alpha[s_1,s_2,s_3];T]$, is composed only of a no-indel history:

$$\left\{ \vec{\hat{M}}(b_1) = \vec{\hat{M}}(b_2) = \vec{\hat{M}}(b_3) = [\ \ ] \right\}, \qquad \text{--- Eq.(SM-4.1)}$$

with $s^{Root} = s_0^{Root} = [L, R]$. Thus, according to Eq.(SM-1.15), supplemented by Eq.(SM-1.16) and Eq.(R1.9), the total parsimonious contribution is:

$$\breve{\mathrm{M}}_{P(0)}[case\ (I)] = 1. \qquad \text{--- Eq.(SM-4.2)}$$

Here we used $\mu_P\left[s_0^{Root}, s_0^{Root}, n^{Root}; C_{\mathrm{K}}\right] = 1$. No single-event local history can result in the gap configuration in this case. Each next-parsimonious indel history consists of two indels, and it can be represented as:

$$\left\{ \vec{\hat{M}}(b_m) = \left[\hat{M}_I(1,l), \hat{M}_D(2,l+1)\right],\ \vec{\hat{M}}(b_{m'}) = [\ \ ]\ \ for\ {}^{\forall}m' \in \{1,2,3\} \setminus \{m\} \right\},$$

--- Eq.(SM-4.3)

with $m \in \{1,2,3\}$, $l \in \left\{1,2,...,L_{:m}^{CO}\ (\equiv \min\{L_{I:m}^{CO}, L_{D:m}^{CO}\})\right\}$, and with $s^{Root} = s_0^{Root}$ again. Thus, the total next-parsimonious contribution can be calculated similarly to Eqs.(SM-2.2a,b). We have:

$$\breve{\mathrm{M}}_{P(0)}[case\ (I)] = \sum_{m=1,2,3} \sum_{l=1}^{L^{CO}} \mu_P\left[\left(\left[\hat{M}_I(1,l), \hat{M}_D(2,l+1)\right], [t_I, t_{F:m}]\right) \middle| \left(s_0^{Root}, t_I\right)\right]\Bigg|_{b_m}. \quad \text{--- Eq.(SM-4.4a)}$$

Each summand can be calculated from Eq.(SM-2.2b), by replacing s^A with s_0^{Root} and also replacing the time and rate parameters with those assigned to each branch. Especially, under Dawg's model, each summand is calculated as:

$$\mu_P\left[\left(\left[\hat{M}_I(1,l), \hat{M}_D(2,l+1)\right], [t_I, t_{F:m}]\right) \middle| \left(s_0^{Root}, t_I\right)\right]\Bigg|_{b_i}$$
$$= \lambda_{I:m} f_{I:m}(l)\ \lambda_{D:m} f_{D:m}(l)\ \frac{\exp\left(-(\lambda_{I:m} + \lambda_{D:m}) l (t_{F:m} - t_I)\right) - 1 + (\lambda_{I:m} + \lambda_{D:m}) l (t_{F:m} - t_I)}{\left((\lambda_{I:m} + \lambda_{D:m}) l\right)^2}.$$

--- Eq.(SM-4.4b)

If the three branches share the same time interval and the indel rate parameters, the total next-parsimonious contribution, Eq.(SM-4.4a), is exactly three times Eq.(SM-2.2a) for a PWA. Indeed, this total next-parsimonious contribution on a general tree can be calculated by summing Eq.(SM-2.2a) (with appropriate parameters) over all branches of the tree. Following the same line of reasoning as around Eq.(SM-2.3), this total contribution is roughly proportional to the summation of the squared branch lengths over all branches. This means that a richer sampling of the homologous sequences will not significantly increase, or might rather slightly decrease, this total contribution, as long as the maximum evolutionary distance

remains at a similar level. Incidentally, any root sequence state of the type $s^{Root} = \left[L, 1,..., \Delta L^{Root}, R\right]$ is also consistent with $\alpha[s_1, s_2, s_3]$ in this case. Such a state, however, would require at least three indels, in order to delete the extra sites ($1,..., \Delta L^{Root}$) independently along the three branches. Thus, the contributions from the local indel histories with such root states would be smaller in general.

Case (II) is represented by the external sequence states $s_1 = s_2 = \left[L, 1,..., \Delta L^{D12}, R\right]$ and $s_3 = \left[L, R\right]$ (Figure 4c). In this case, the "phylogenetic correctness" condition (see, *e.g.*, [46,47]) dictates that the root state s^{Root} must have all the sites with ancestries $1,..., \Delta L^{D12}$. In this case, we have $N_{\min}\left[case\,(II)\right] = 1$. And the set of parsimonious local histories, $\Lambda_{\Psi}^{ID}\left[N_K = 1; C_K; \alpha[s_1, s_2, s_3]; T\right]$, consists of a single element:

$$\left\{\vec{\hat{M}}(b_1) = \vec{\hat{M}}(b_2) = [\ ], \vec{\hat{M}}(b_3) = \left[\hat{M}_D(2, \Delta L^{D12} + 1)\right]\right\}, \quad \text{--- Eq.(SM-4.5)}$$

with $s^{Root} = s_0^{Root} = \left[L, 1,..., \Delta L^{D12}, R\right]$. Again, according to Eq.(SM-1.15) supplemented by Eq.(SM-1.16) and Eq.(R1.9), the total parsimonious contribution turns out to be exactly the same as Eq.(SM-2.4) for case (ii) of PWAs, with the parameters replaced with those assigned to the branch b_3, and with ΔL^A replaced with ΔL^{D12}. Especially, under Dawg's model, we have:

$$\breve{\mathrm{M}}_{P(1)}\left[case\,(II); \Delta L^{D12}\right] = \lambda_{D:3} f_{D:3}(\Delta L^{D12}) \frac{\exp\left((\lambda_{I:3} + \lambda_{D:3})\Delta L^{D12}(t_{F:3} - t_I)\right) - 1}{(\lambda_{I:3} + \lambda_{D:3})\Delta L^{D12}}.$$

--- Eq.(SM-4.6)

As in case (ii) of PWAs, each next-parsimonious indel history is composed of two indel events, and there are two types of such histories. One is based on type (a) in case (ii):

$$\left\{\vec{\hat{M}}(b_1) = \vec{\hat{M}}(b_2) = [\ ], \vec{\hat{M}}(b_3) = \left[\hat{M}_D(x, x + l - 1), \hat{M}_D(2, \Delta L^{D12} - l + 1)\right]\right\}, \quad \text{--- Eq.(SM-4.7a)}$$

with $l = 1,..., \Delta L^{D12} - 1$, $x = 2,..., \Delta L^{D12} - l + 2$, and also with $s^{Root} = s_0^{Root}$. And the other is based on type (b) in case (ii):

$$\left\{\vec{\hat{M}}(b_1) = \vec{\hat{M}}(b_2) = [\ ], \vec{\hat{M}}(b_3) = \left[\hat{M}_I(x, l), \hat{M}_D(2, \Delta L^{D12} + l + 1)\right]\right\}, \quad \text{--- Eq.(SM-4.7b)}$$

with $l = 1,..., \min\{L_{I:3}^{CO}, L_{D:3}^{CO} - \Delta L^{D12}\}$, $x = 1,..., \Delta L^{D12} + 1$, and also with $s^{Root} = s_0^{Root}$ again.

Thus the total next-parsimonious contribution is given by:

$$\breve{\tilde{\mathrm{M}}}_{P(2)}\left[case\,(II);\,\Delta L^{D12}\right] = \breve{\mathrm{M}}_P[(a);\,\Delta L^{D12}] + \breve{\mathrm{M}}_P[(b);\,\Delta L^{D12}]\,. \quad \text{--- Eq.(SM-4.8)}$$

Here $\breve{\tilde{\mathrm{M}}}_P[(a);\,\Delta L^{D12}]$ and $\breve{\tilde{\mathrm{M}}}_P[(b);\,\Delta L^{D12}]$ are given by exactly the same equations as Eqs.(SM-2.5b,d) and Eqs.(SM-2.5c,e), respectively, with the aforementioned due replacements. Especially, under Dawg's model, the contributions by the individual next-parsimonious indel histories are given by Eqs.(SM-2.5d',e') with the due replacements. Thus, Figure S4 can also be interpreted exactly as the comparison between the total parsimonious contribution and the total next-parsimonious contribution in the current case. Incidentally, root sequences with some additional ancestral sites in between the PASs of $s_0^{Root} \equiv \left[L, 1,...,\Delta L^{D12}, R\right]$ are also consistent with $\alpha[s_1,s_2,s_3]$ in this case. However, such root sequence states require at least three indels each to give rise to $\alpha[s_1,s_2,s_3]$. This is because the additional ancestral sites need to be deleted independently along b_1 and b_2, even if they are deleted simultaneously with the sites with the ancestries $1,...,\Delta L^{D12}$ along b_3. Thus, in general, the indel histories consistent with such root states are expected to contribute much less to the multiplication factor.

Case (III) is represented by the external sequence states $s_1 = \left[L, 1,...,\Delta L^{D1}, R\right]$ and $s_2 = s_3 = \left[L, R\right]$ (Figure 4d). In this case, the phylogenetic correctness condition does not require the root state to have any site in between the PASs. Thus we have $s_0^{Root} = \left[L, R\right]$. As in case (II), we have $N_{\min}\left[case\,(III)\right] = 1$. And the set of parsimonious local histories, $\Lambda_{\Psi}^{ID}\left[N_{\mathrm{K}} = 1; C_{\mathrm{K}}; \alpha[s_1,s_2,s_3]; T\right]$, consists of a single element:

$$\left\{\vec{\hat{M}}(b_1) = \left[\hat{M}_I(1, \Delta L^{D1})\right], \vec{\hat{M}}(b_2) = \vec{\hat{M}}(b_3) = [\;\;]\right\}, \quad \text{--- Eq.(SM-4.9)}$$

with $s^{Root} = s_0^{Root}$. Again, as in case (II), the contribution by this local history turns out to be exactly the same as Eq.(A1.1.1) (in Appendix A1.1 of [43]) for case (iii) of PWAs, with the parameters replaced with those assigned to the branch b_1, and with ΔL^D replaced with ΔL^{D1}. Especially, under Dawg's model, we have:

$$\breve{\mathrm{M}}_{P(1)}\left[case\,(III);\,\Delta L^{D1}\right] = \lambda_{I:1} f_{I:1}(\Delta L^{D1})\,\frac{1-\exp\left(-(\lambda_{I:1}+\lambda_{D:1})\Delta L^{D1}(t_{F:1}-t_I)\right)}{(\lambda_{I:1}+\lambda_{D:1})\Delta L^{D1}}\,. \quad \text{--- Eq.(SM-4.10)}$$

As in case (iii) of PWAs, each next-parsimonious indel history is composed of two indel events. Unlike case (iii) of PWAs, however, there are three types of such histories. Two of them (classified as "type (A)") are similar to those in case (iii), but the other one (classified as

“type (B)”) is totally new. Specifically, the first one is based on type (c) in case (iii):

$$\left\{\vec{\hat{M}}(b_1)=\left[\hat{M}_I(1,\Delta L^{D1}-l),\hat{M}_I(x,l)\right],\vec{\hat{M}}(b_2)=\vec{\hat{M}}(b_3)=[\ ]\right\},\qquad \text{--- Eq.(SM-4.11a)}$$

with $l=1,...,\Delta L^{D1}-1$, $x=1,...,\Delta L^{D1}-l+1$, and also with $s^{Root}=s_0^{Root}$. The second one is based on type (d) in case (iii):

$$\left\{\vec{\hat{M}}(b_1)=\left[\hat{M}_I(1,\Delta L^{D1}+l),\hat{M}_D(x,x+l-1)\right],\vec{\hat{M}}(b_2)=\vec{\hat{M}}(b_3)=[\ ]\right\},\qquad \text{--- Eq.(SM-4.11b)}$$

with $l=1,...,\min\{L_{D:1}^{CO},L_{I:1}^{CO}-\Delta L^{D1}\}$, $x=2,...,\Delta L^{D1}+2$, and also with $s^{Root}=s_0^{Root}$ again. The third one involves events along b_2 and b_3, instead of along b_1:

$$\left\{\vec{\hat{M}}(b_1)=[\ ],\vec{\hat{M}}(b_2)=\vec{\hat{M}}(b_3)=\left[\hat{M}_D(2,\Delta L^{D}+1)\right]\right\}.\qquad \text{--- Eq.(SM-4.11c)}$$

It is consistent with the root state $s^{Root}=s_1=\left[L,1,...,\Delta L^{D1},R\right]$ instead of $s_0^{Root}=\left[L,R\right]$. It should be noted that there is only one local history of the third type. In this case, therefore, the total next-parsimonious contribution is given by:

$$\breve{\mathrm{M}}_{P(2)}\left[case\,(III);\,\Delta L^{D1}\right]=\breve{\mathrm{M}}_{P(2)}\left[case\,(III);\,(A);\,\Delta L^{D1}\right]+\breve{\mathrm{M}}_{P(2)}\left[case\,(III);\,(B);\,\Delta L^{D1}\right]$$

--- Eq.(SM-4.12a)

$$\breve{\mathrm{M}}_{P(2)}\left[case\,(III);\,(A);\,\Delta L^{D1}\right]\equiv\breve{\mathrm{M}}_{P}[(c)]+\breve{\mathrm{M}}_{P}[(d)],\qquad \text{--- Eq.(SM-4.12b)}$$

$$\breve{\mathrm{M}}_{P(2)}\left[case\,(III);\,(B);\,\Delta L^{D1}\right]\equiv\breve{\mathrm{M}}_{P}[(3rd)].\qquad \text{--- Eq.(SM-4.12c)}$$

Here, $\breve{\mathrm{M}}_{P}[(c)]$ and $\breve{\mathrm{M}}_{P}[(d)]$ are the summed contributions of the type (c)-based and type (d)-based histories, respectively. They are given by exactly the same equations as Eqs.(A1.1.2b,d) and Eqs.(A1.1.2c,e), respectively, in [43], with the aforementioned due replacements. Under Dawg’s model, these two terms are given by summations of Eqs.(A1.1.2d’,e’) in [43] with the due replacements. Thus, Figure S4 can also be interpreted as the comparison between the total contribution of these two types of next-parsimonious indel histories ($\breve{\mathrm{M}}_{P(2)}\left[case\,(III);\,(A);\,\Delta L^{D1}\right]$) and the total parsimonious contribution ($\breve{\mathrm{M}}_{P(1)}\left[case\,(III);\,\Delta L^{D1}\right]$). Meanwhile, $\breve{\mathrm{M}}_{P}[(3rd)]$ is the contribution from the unique next-parsimonious indel history of the 3rd type (*i.e.*, type (B)), Eq.(SM-4.11c). According to Eq.(SM-1.15) supplemented by Eq.(SM-1.16) and Eq.(R1.9), it is expressed as:

$$\breve{\mathrm{M}}_P[(3rd)] = \mu_P\left[s^{Root} = s_1, s_0^{Root}, n^{Root}; C_{\mathrm{K}}\right] \exp\left\{ - \sum_{m=1,2,3} \int_{t_I}^{t_{F:m}} dt\, \delta R_{X:m}^{ID}(s^{Root} = s_1, s_0^{Root}, t)\Big|_{b_m} \right\}$$

$$\times \prod_{m=2,3} \int_{t_I}^{t_{F:m}} dt \left[r_{D:m}(2, \Delta L^{D1}+1; s^{Root} = s_1, t) \exp\left\{ -\int_t^{t_{F:i}} d\tau\, \delta R_{X:m}^{ID}(s_m, s^{Root} = s_1, t) \right\}\Big|_{b_m} \right].$$

--- Eq.(SM-4.13)

Under Dawg's model, we have $\delta R_{X:m}^{ID}(s^{Root} = s_1, s_0^{Root}, t)\big|_{b_m} = (\lambda_{I:m} + \lambda_{D:m})\Delta L^{D1}$ for $m = 1,2,3$, and $\delta R_X^{ID}(s_m, s^{Root} = s_1, t)\big|_{b_m} = -(\lambda_{I:m} + \lambda_{D:m})\Delta L^{D1}$ for $m = 2,3$. Moreover, if we assume the uniform distribution of the root sequence length, we have $\mu_P\left[s^{Root} = s_1, s_0^{Root}, n^{Root}; C_{\mathrm{K}}\right] = 1$. Thus, Eq.(SM-4.13) is reduced to:

$$\breve{\mathrm{M}}_P[(3rd)] = \exp\left(-(\lambda_{I:1} + \lambda_{D:1})\Delta L^{D1}(t_{F:1} - t_I)\right)$$

$$\times \prod_{m=2,3} \left\{ \lambda_{D:m} f_{D:m}(\Delta L^{D1}) \frac{1 - \exp\left(-(\lambda_{I:m} + \lambda_{D:m})\Delta L^{D1}(t_{F:m} - t_I)\right)}{(\lambda_{I:m} + \lambda_{D:m})\Delta L^{D1}} \right\}.$$

--- Eq.(SM-4.13')

Figure 5 shows the ratio of $\breve{\mathrm{M}}_P[(3rd)]$ $(= \breve{\mathrm{M}}_{P(2)}\left[case\,(III); (B); \Delta L^{D1}\right])$ to the total parsimonious contribution, Eq.(SM-4.10), when all three branches have the same length and are assigned the same indel model as that used for Figure S4. Because the ratio compares the multiplication factors concerning the indel events along different branches, its value actually depends on several factors. It would be convenient to keep in mind that the ratio could be approximated by $\lambda_{D:2} f_{D:2}(\Delta L^{D1})(t_{F:2} - t_I)\, \lambda_{D:3} f_{D:3}(\Delta L^{D1})(t_{F:3} - t_I) \big/ \left[\lambda_{I:1} f_{I:1}(\Delta L^{D1})(t_{F:1} - t_I)\right]$ when $(\lambda_{I:m} + \lambda_{D:m})\Delta L^{D1}(t_{F:m} - t_I)$'s are sufficiently smaller than 1 for all $m = 1,2,3$. In general, as ΔL^{D1} gets larger, the ratio is expected to decrease, because the relative frequencies of long indels ($f_{I:m}(\Delta L^{D1})$ and $f_{D:m}(\Delta L^{D1})$) are small in general. The ratio is expected to be much smaller than 1 in general. However, it may become quite large when the relative frequency of deletions compared to insertions (*i.e.*, the ratio $\lambda_{D:m}/\lambda_{I:m}$) is considerably larger than 1, or when the lengths of b_2 and b_3 are much larger than that of b_1 (*i.e.*, $t_{F:2} - t_I, t_{F:3} - t_I >> t_{F:1} - t_I$). Such situations are similar to those causing the "Felsenstein zone" regarding a substitution model, where a non-parsimonious substitution history at a site is most likely to occur along a tree (see, *e.g.*, Chapter 9 of [5]). Under the conditions used to draw Figure 5, an indel history of the 3rd type (*i.e.*, type (B)) has a probability much smaller than

that of the parsimonious indel history. The former is less than 5% of the latter even when as much as 0.4 indels per site are expected to occur. This is probably because the type (B) history requires an exquisite spatial coordination of deletions along different branches. And the result implies that the "Felsenstein zone" of indels should generally be quite narrow, consisting of the cases where a node is connected with branches with extremely unequal lengths.

Case (IV) is represented by the external sequence states, $s_1 = \left[L, 1, ..., \Delta L^{D1}, R\right]$, $s_2 = \left[L, 1, ..., i, j, ..., \Delta L^{D1}, R\right]$, and $s_3 = \left[L, R\right]$, with $1 \leq i+1 < j \leq \Delta L^{D1}+1$ but $(i, j) \neq (0, \Delta L^{D1}+1)$ (Figure 4e). (Here "$1, ..., 0$" and "$\Delta L^{D1}+1, ..., \Delta L^{D1}$" should be considered to be empty.) In this case, the phylogenetic correctness condition requires the root state to have sites with ancestries $1, ..., i$ and $j, ..., \Delta L^{D1}$, on top of the PASs. Thus, we have $s_0^{Root} = s_2 = \left[L, 1, ..., i, j, ..., \Delta L^{D1}, R\right]$. Here, the minimum number of indels is $N_{\min}\left[case\ (IV)\right] = 2$. And the set of parsimonious local histories, $\Lambda_{\Psi}^{ID}\left[N_{\mathrm{K}} = 2; C_{\mathrm{K}}; \alpha[s_1, s_2, s_3]; T\right]$, consists of *two* histories. One starts with the root state $s^{Root} = s_0^{Root}\ (= s_2)$, and is represented as:

$$\left\{\vec{\hat{M}}(b_1) = \left[\hat{M}_I(i+1, j-i-1)\right], \vec{\hat{M}}(b_2) = [\ ], \vec{\hat{M}}(b_3) = \left[\hat{M}_D(2, \Delta L^{D1} - j + i + 2)\right]\right\}.$$

--- Eq.(SM-4.14a)

The other starts with the root state $s^{Root} = s_1 = \left[L, 1, ..., \Delta L^{D1}, R\right]$, which differs from $s_0^{Root}\ (= s_2)$. It is represented as:

$$\left\{\vec{\hat{M}}(b_1) = [\ ], \vec{\hat{M}}(b_2) = \left[\hat{M}_D(i+2, j)\right], \vec{\hat{M}}(b_3) = \left[\hat{M}_D(2, \Delta L^{D1}+1)\right]\right\}.$$ --- Eq.(SM-4.14b)

The total parsimonious contribution and the total next-parsimonious contribution are calculated in Appendix A2 of [43].

SM-5. Algorithm to compute first-approximate MSA probability

As briefly mentioned in section R5 of Results and discussion, we developed an algorithm that, under a given phylogenetic tree of the sequences and a given indel model (including its parameters), calculates the first-approximate probability that a given MSA actually occurs, using only the parsimonious indel histories consistent with the MSA. As a byproduct, the algorithm also calculates the relative probabilities among the parsimonious indel histories

(more precisely, among the parsimonious ancestral state sets). In this section, we will describe the algorithm. Then, in Sections SM-6,7,8, we will describe some analyses that were performed to validate the algorithm.

In this study, when we refer to a "MSA," we consider only its *homology structure* [39], and other details (including residue states) are ignored. For example, the "probability of a MSA" means the probability of *the homology structure of* the MSA under a given *genuine* indel evolutionary model. It should be noted here that the algorithm proposed here *assumes that the input MSA is correct*. Under this assumption, the algorithm approximately calculates the probabilities concerning the MSA.

SM-5.1. Outline

Panel A of Figure S5 shows a flowchart of the procedures comprising our entire algorithm. Broadly speaking, the algorithm consists of three parts: (i) the "pre-processing" procedures that finally partition the entire input MSA into gapped segments (*i.e.*, local MSAs) and gapless segments separating them (steps ia-ic); (ii) enumerating the parsimonious local indel histories that can explain each gapped segment (step ii); and (iii) calculating the first-approximation of the augmented multiplication factor ($\breve{\mathrm{M}}_{P(N_{\min}[C_{\mathrm{K}}])}\left[\alpha[s_1, s_2, \ldots, s_{N^X}]; s_0^{Root}; C_{\mathrm{K}} \,\middle|\, T\right]$, given by Eq.(SM-1.15)) contributed from each gapped segment (C_{K}) (step iii). The final results thus produced are put together, along with the overall factor (Eq.(SM-1.13)) to provide the first-approximation of the total occurrence probability of the entire MSA (Eq.(SM-1.12)) as well as the relative probabilities among the parsimonious local indel histories that could explain each gapped segment (step iv). Panel B of Figure S5 schematically illustrates the procedures constituting the pre-processing part (steps ia-ic). The steps (ii) and (iii) will be described in Subsections SM-5.2 and SM-5.3, respectively.

After an input MSA is given [Figure S5, step (o)], the algorithm first reduces the MSA to a binary pattern. In the binary pattern, each cell specified by a row (sequence) and a column (site) is given any of the two states: "presence" (denoted as "1") when the cell is occupied by a residue, or "absence" (denoted as "0") when it is filled with a gap [step (ia)]. Then the algorithm decomposes the MSA into "gap-pattern block"s, or "block"s for short, each of which consists of contiguous columns with the same presence/absence pattern [step (ib)]. Among such blocks, those containing no absence state play a distinct role as separators. If the MSA is correct, the existence of a gapless column indicates that no indel events occurred on or pierced the column. This is a corollary of the phylogenetic correctness

condition (*e.g.*, [46,47]). Thus, gapless columns flanking a gapped segment *genuinely* delimit the indel events potentially responsible for the segment, *even if they constitute non-parsimonious histories*. We have another note. In general, two contiguous gapless columns do not preclude indels in between them (*e.g.*, a next-parsimonious history, Eq.(SM-4.3), yielding a case (I) local MSA). Nevertheless, the algorithm described here ignores such indels between contiguous gapped columns, because it is only interested in parsimonious indel histories.

Then the algorithm makes a "cluster" out of a run of contiguous blocks containing the absence state and not separated from each other by gapless columns [step (ic)]. Thus, each cluster spans between a gapless segment and the next gapless segment (or a MSA end). In this paper, we simply refer to such a cluster of gap-pattern blocks as a "gapped segment" (or a local MSA). As explained in [22], indel events and the probability of a local indel history in each gapped segment can be considered independently of events in the other gapped segments (even if we allow for non-parsimonious indel histories), as long as the indel model fulfills conditions (i), (ii) and (iii) (given in section R1 of Results and discussion).

After the pre-processing part (step (i)) explained above, the two core parts follow: enumerating parsimonious local indel histories for each gapped segment (step (ii)), and calculating the multiplication factor from the segment (step (iii)). They will be explained in SM-5.2 and SM-5.3 below. The "post-processing" step (iv) will also be explained in SM-5.3.

SM-5.2. Enumerating all parsimonious local indel histories

The first core part of our algorithm is itself an algorithm. It attempts to enumerate all parsimonious local indel histories that can yield each gapped segment. This core part consists of two subparts. (1) First it constructs an initial candidate for the local parsimonious indel histories, by identifying the unique Dollo parsimonious history [64] for each gap-pattern block, and by merging together indel events of the same type in effectively contiguous blocks and along the same branch of the phylogenetic tree (Figure S6). (2) Then it iteratively searches for local indel histories whose events are fewer than or as many as those in the current candidate parsimonious histories (Figure S7). And it updates the set of candidate histories if such a history is found. It should be noted that, because we consider the input MSA to have resulted from an evolutionary process, the candidate indel histories must conform to the phylogenetic correctness condition (*e.g.*, [46,47]). We used the Dollo parsimonious state [64] (in each gap-pattern block) as a starting point because it conforms to this condition. [NOTE: The Dollo parsimony criterion [64] seeks for an indel history consisting of the fewest events that can explain the gap-pattern, while only allowing for at

most one insertion (per column or block) in order to keep the phylogenetic correctness.] In the following, we will explain these sub-parts in more detail.

(1) *Constructing an initial candidate of parsimonious local indel histories*. The first candidate history is constructed based on the block-wise Dollo parsimonious indel histories. The Dollo parsimonious indel history for each block can be easily and quickly constructed by a round-trip traversal of the (rooted) phylogenetic tree, first bottom-up and second top-down. In the bottom-up traversal, each node (n) is assigned the number of child nodes each of which has at least one extant descendant node with the "presence" state. Let the number denoted as $N_{CDP}(n)$. When reaching the top (*i.e.*, the root node n^{Root}), the root is assigned the "presence" state if $N_{CDP}(n^{Root}) \geq 2$, otherwise it is assigned the "absence" state. Then, in the top-down traversal, each node (again n) is assigned the "presence" state, either if (a) $N_{CDP}(n) \geq 2$, or if (b) $N_{CDP}(n) = 1$ and its parent is assigned the "presence" state. Otherwise, the node is assigned the "absence" state. Then, indels are inferred to have occurred only along the branches whose ends are in different "presence"/"absence" states. Once the Dollo parsimonious history is constructed for each block belonging to the gapped segment, the algorithm tries to reduce the number of indels by merging the effectively contiguous indel events of the same type (either all insertions or all deletions) and along the same branch in the sequence phylogeny (Figure S6). The "effectively contiguous" indel events can be either events in literally contiguous blocks (Figure S6, panel A) or events separated only by a (run of) block(s) that is (are) devoid of the "presence" state in any 'downstream' nodes (in the virtual temporal direction such that the event is viewed as a 'deletion') (panel B). [NOTE: What the single-quotes exactly mean will be explained below (in SM-5.2.1).] When two events of the same type along the same branch are intervened by a block with the "presence" state in some 'downstream' nodes (the red "1" in panel C), however, the events are left unmerged. [NOTE: If we deal with the homology structure *at this stage*, the events could be merged even in the situation in Figure S6C. For future use, however, we decided that this stage should deal with each gap configuration *faithfully* as created by a true indel process, even if its homology structure indicates other treatments. And we also decided that each input MSA should be converted to its homology structure *at a pre-processing step* (detailed in Methods of [38]).] In most cases, this sub-part determines the unique parsimonious local indel history for each gapped segment.

(2) *Iteratively updating the set of candidate parsimonious local indel histories*. Not always and yet considerably frequently, the first sub-part doesn't suffice to enumerate the parsimonious local indel histories. For example, in the situation illustrated in Figure S7, panel A, there could be another parsimonious history (panel C) on top of the initial history

constructed in the first sub-part (panel B). In another example (panel D), there even exists a history (panel F) that requires less indel events than the intermediate candidate history (panel E), which requires as many indels as the initial, Dollo parsimonious history (not shown). Such histories can be found by iteratively updating the set of candidate parsimonious local indel histories, via "branch-and-merge" operations (panels G, H, F). A branch-and-merge operation begins with "branching" a 'deletion' event, that is, re-interpreting a 'deletion' event along a branch (panel G) as multiple independent 'deletions', each along one of the 'child' branches (panel H). [NOTE: The single-quotes will be explained below (in SM-5.2.1).] Then, the "merging" process merges each resulting 'deletion' event with the effectively contiguous 'deletion' event(s), if at all, creating a new local indel history (panel F in this example). If the newly created history requires fewer indel events than the current candidate histories, then the new history replaces the current candidates. If the new history requires as many events as the current candidate(s), it joins the set of current candidates. Otherwise, the new history is discarded and, if some special conditions are met, the algorithm tries a more complex "branch-and-merge" operation as an attempt to exhaust all promising histories (detailed in M1.2.2 of [48]).

If you will, this second sub-part could be called a **"local multi-path downhill search algorithm."** From each point, *i.e.*, a local indel history, it examines only its neighborhoods, which are separated from the point by a single "branch-and-merge" operation. In this sense, it is a "**local** search." Then, it keeps *only* those histories that consists of fewer indels than, or as few indels as, the current candidate. Thus it is "**downhill**." At the same time, it keeps *all* histories that are found to have the same, "current-smallest," number of indels. Hence it has the qualifier, "**multi-path**."

SM-5.2.1. Assigning virtual temporal directions and ordering indel events

To exhaust (almost) all promising histories, the "branch-and-merge" processes (explained in SM-5.2, item (2)) are iterated from the "most influential" 'deletion' events to the "least influential" 'deletion' events (Figure S8). (Each "most influential" 'deletion' event 'deletes' a relevant sub-sequence from the largest number of aligned sequences.) Let us first explain what these single-quoted terms mean.

First, if the tree of the aligned sequences is rooted (panel A of Figure S8, left), it is converted to an unrooted tree (panel B). Then, all the indel events (panel A, right) are re-interpreted as 'deletions' (panel C). This could be done because the time direction could be arbitrarily assigned on an unrooted tree, and because an insertion can be regarded as a deletion in the opposite time direction. The time direction may not be assigned consistently to

all branches, for example when insertions and deletions co-exist along a branch. However, this doesn't matter and we will assign a unique '*virtual* time direction' *to each indel event*, because it is only 'deletion' events with consistent directions that can be merged together, and because this re-interpretation is just a means to determine the order of the events that will go through the "branch-and-merge" operations. (And a term will be single-quoted when it applies under this virtual time direction.)

Now, the 'deletions' will be sorted in descending order of the number of 'deleted' sequences (panel D), and they will be processed from top to bottom of the list. The order is determined uniquely, except the ambiguity in the ordering among events that 'delete' the same number of sequences. This ambiguity is not expected to matter seriously, because such events won't be merged together in any "branch-and-merge" process.

A list of 'deletions' to be examined accompanies each candidate local indel history. Each time a "branch-and-merge" operation is tried on the 'deletion' at the top of the list, the list is updated by removing the top 'deletion' just examined. If a "branch-and-merge" operation succeeds in finding a new promising candidate history, the new history is accompanied by a new list created by replacing the examined top 'deletion' with the resulting new 'deletion(s).' (The latter will be incorporated in the right order according to the number of aligned sequences that the sub-sequence was 'deleted from.')

SM-5.3. First-approximate calculation of absolute occurrence probability and relative probabilities

The second core part of our algorithm calculates the first approximation of the *ab initio* occurrence probability of a given entire MSA under a given phylogenetic tree and a given indel evolutionary model, using only the contributions from parsimonious indel histories that are consistent with the MSA. The calculation is based on Eqs.(SM-1.12-16) for the probability of a given MSA, $\alpha[s_1, s_2, ..., s_{N^X}]$. [NOTE: The current latest version actually calculates the MSA probability based on Eqs.(SM-4.20-22,18,13) of [22]. Together, these equations express the multiplication factor from a local MSA as the product of the multiplication factors of the constituent local PWAs.] The current version of the implementation of this core part only calculates the probability under Dawg's indel model [32], whose indel rate parameters (Eqs.(R1.7,8,9)) are spatially and temporally homogeneous. And the current version uses exclusively a uniform length distribution of the ancestral sequence (s^{Root}) at the root (n^{Root}):

$$P\left[\left(s^{Root}, n^{Root}\right)\right] \propto 1. \quad \text{--- Eq.(SM-5.3.1)}$$

Thus, we always have $\mu_P\left[s^{Root}, s_0^{Root}, n^{Root}; C_{\mathrm{K}}\right]=1$ for every possible s^{Root} and for every potentially indel-accommodating region (C_{K} with $^{\forall}\mathrm{K}\in\{1,...,\mathrm{K}_{\max}\}$). Here, as the "reference root state" (s_0^{Root}), we do *not* use the concatenated root states of the block-wise Dollo parsimonious indel histories. Instead, as s_0^{Root}, we use an array consisting solely of all sites corresponding to the gapless columns. Under a space-homogeneous model, this poses no problem. Let $N_{GLC}\left(\alpha[s_1,s_2,...,s_{N^X}]\right)$, or N_{GLC} for short, be the number of gapless columns in $\alpha[s_1,s_2,...,s_{N^X}]$. Then, from Eq.(R1.9), we have:

$$R_X^{ID}(s_0^{Root},t)=(\lambda_I+\lambda_D)N_{GLC}+\Delta^{Dawg}[\lambda_I,\lambda_D,f_D(.)],$$ --- Eq.(SM-5.3.2)

$$\delta R_X^{ID}(s,s_0^{Root},t)[C_{\mathrm{K}}]=(\lambda_I+\lambda_D)\left\{L\left(s[C_{\mathrm{K}}]\right)-L\left(s_0^{Root}[C_{\mathrm{K}}]\right)\right\}=(\lambda_I+\lambda_D)L\left(s[C_{\mathrm{K}}]\right).$$

--- Eq.(SM-5.3.3)

Here, $s[C_{\mathrm{K}}]$ is the sub-sequence of the sequence s confined in the region C_{K}. We also used the fact that $s_0^{Root}[C_{\mathrm{K}}]$ is always empty.

Then, the first approximation of each multiplication factor (SM-1.14) is given by:

$$\tilde{\breve{\mathrm{M}}}_P^{\langle 1st\rangle}\left[\alpha[s_1,s_2,...,s_{N^X}];s_0^{Root};C_{\mathrm{K}}\mid T\right]\equiv\breve{\mathrm{M}}_{P\,(N=N_{\min}[C_{\mathrm{K}}])}\left[\alpha[s_1,s_2,...,s_{N^X}];s_0^{Root};C_{\mathrm{K}}\mid T\right],$$

--- Eq.(SM-5.3.4)

where the right hand side is given by Eq.(SM-1.15) with $N=N_{\min}[C_{\mathrm{K}}]$. Using this, the first approximation of the probability of the entire MSA is given by a reduced form of (Eq.R1.5) (or Eq.(SM-1.12)). Their explicit expressions are:

$$P^{\langle 1st\rangle}\left[\alpha[s_1,s_2,...,s_{N^X}]\mid T\right]=P_0\left[s_0^{Root}\mid T\right]\prod_{\mathrm{K}=1}^{\mathrm{K}_{\max}}\tilde{\breve{\mathrm{M}}}_P^{\langle 1st\rangle}\left[\alpha[s_1,s_2,...,s_{N^X}];s_0^{Root};C_{\mathrm{K}}\mid T\right],$$

--- Eq.(SM-5.3.5a)

with

$$P_0\left[s_0^{Root}\mid T\right]\equiv P\left[\left(s_0^{Root},n^{Root}\right)\right]\times\exp\left\{-(\lambda_I+\lambda_D)\,|T|N_{GLC}+\Delta^{Dawg}[\lambda_I,\lambda_D,f_D(.)]\,|T|\right\}.$$

--- Eq.(SM-5.3.5b)

Here, $|T|\equiv\sum_{b\in\{b\}_T}|b|$ is the total length over all branches in the tree (T). In general, the set of regions that can accommodate local indel histories, $\{C_{\mathrm{K}}\}_{\mathrm{K}=1,...,\mathrm{K}_{\max}}$, also contains the positions sandwiched by adjacent gapless columns within each single gapless segment. In the

first approximation, the contribution, $\tilde{\tilde{\mathrm{M}}}_P^{\langle 1st\rangle}\left[\alpha[s_1,s_2,...,s_{N^X}];\, s_0^{Root};\, C_{\mathrm{K}} \mid T\right]$, from each such sandwiched position is always trivial (*i.e.*, unity). Thus, Eq.(SM-5.3.5a) could be further simplified as:

$$P^{\langle 1st\rangle}\left[\alpha[s_1, s_2,..., s_{N^X}] \mid T\right] = P_0\left[s_0^{Root} \mid T\right] \prod_{K=1}^{K_{\max}^0} \tilde{\tilde{\mathrm{M}}}_P^{\langle 1st\rangle}\left[\alpha[s_1,s_2,...,s_{N^X}];\, s_0^{Root};\, C_{\mathrm{K}}^0 \mid T\right].$$

--- Eq.(SM-5.3.5a')

Here, $\left\{C_K^0\right\}_{K=1,...,K_{\max}^0}$ is the set of all gapped segments in the MSA. It is a subset of $\left\{C_{\mathrm{K}}\right\}_{\mathrm{K}=1,...,\mathrm{K}_{\max}}$, and thus $K_{\max}^0 \le \mathrm{K}_{\max}$ always holds. This Eq.(SM-5.3.5a'), supplemented by Eqs.(SM-1.15,16) and Eqs.(SM-5.3.3,5b), is the major output of the second core part, and of the entire algorithm. [NOTE: But the current latest version uses the local-PWA-based expressions, as noted around the top of this subsection.]

As a byproduct, the second core part also outputs the relative probabilities among the parsimonious local indel histories that can give rise to the same local MSA confined in each gapped segment, C_K^0. The relative probability of each parsimonious local history, $\left\{\vec{\hat{M}}(b)\right\}_T\left[C_K^0\right]$, is calculated as:

$$\begin{aligned} P_{\mathrm{Re}l}^{\langle 1st\rangle}\left[\left\{\vec{\hat{M}}(b)\right\}_T\left[C_K^0\right]\right] &\equiv P\left[\left\{\vec{\hat{M}}(b)\right\}_T\left[C_K^0\right] \,\middle|\, \Lambda_\Psi^{ID}\left[N = N_{\min}[C_K^0];\, C_K^0;\, \alpha[s_1,s_2,...,s_{N^X}];\, T\right]\right] \\ &= \breve{\mathrm{M}}_P\left[\left\{\vec{\hat{M}}(b)\right\}_T\left[C_K^0\right];\, s_0^{Root};\, T\right] \Big/ \tilde{\tilde{\mathrm{M}}}_P^{\langle 1st\rangle}\left[\alpha[s_1,s_2,...,s_{N^X}];\, s_0^{Root};\, C_K^0 \mid T\right] \end{aligned}.$$

--- Eq.(SM-5.3.6a)

with

$$\begin{aligned} &\breve{\mathrm{M}}_P\left[\left\{\vec{\hat{M}}(b)\right\}_T\left[C_K^0\right];\, s_0^{Root};\, T\right] \\ \equiv\ & \mu_P\left[s^{Root}, s_0^{Root}, n^{Root};\, C_{\mathrm{K}}\right] \mathrm{M}_P\left[\left\{\vec{\hat{M}}(b)\right\}_T\left[C_K^0\right] \,\middle|\, \left(s^{Root}, n^{Root}\right)\right] \\ &\times \exp\left\{-\sum_{b\in\{b\}_T} \int_{t(n^A(b))}^{t(n^D(b))} d\tau\, \delta R_X^{ID}(s^A(b), s_0^{Root}, \tau)[C_{\mathrm{K}}]\right\}\Bigg|_{\substack{s(n^{Root})=s^{Root},\\ \langle s^D(b)| = \langle s^A(b)|\hat{M}_1(b)\cdots\hat{M}_{N(b)}(b) \\ for\ \forall b\in\{b\}_T}} \end{aligned}.$$

--- Eq.(SM-5.3.6b)

SM-6. Comparing parsimonious local indel histories with true history

Rigorously speaking, unless the record is kept on the true local indel history that created each observed gapped segment, we cannot compare it with the predicted (parsimonious) histories. Nevertheless, if we have the ancestral sequence states at all the internal nodes aligned with the "extant" sequences at the external nodes, we can *approximately* judge whether the true local indel history matches one of the predicted (parsimonious) histories, by comparing the gap states of all the true ancestral sequences (in the segment in question) with the ancestral gap states in each predicted history. [It should be noted, however, that the judgment is only approximately correct, because the same set of ancestral sequence states could result from more than one local indel history if non-parsimonious histories are also allowed.] Dawg [32] can output the alignment of ancestral sequences at the labeled internal nodes with the "extant" sequences at the external nodes. Thus, we took advantage of this function and examined whether the true ancestral gap states in each instance of a gapped segment match those predicted by one of the parsimonious local indel histories. [NOTE: More precisely speaking, we compared the ancestral sequence states superposed to *the homology structure of* each local MSA of extant sequences.] If there is a match, we registered the instance as "parsimonious," and recorded which parsimonious history can produce the true ancestral gap states. Otherwise, we registered the instance as "non-parsimonious." Dawg sometimes creates gapped segments containing null columns, in each of which all extant sequences are occupied by gaps. In this study, such null columns were simply removed before the analyses.

SM-7. Correlation analysis to validate predicted absolute occurrence probabilities of gapped segments

To examine whether or not our first approximation of the augmented multiplication factor, Eq.(SM-5.3.4), works well, we first counted instances of gapped segments that occurred in each of the simulated sets A1 and A2 without reaching either MSA end, and that showed a particular gap-configuration (more precisely, a homology structure), say, G_a. Then we compared the count of instances (*i.e.*, the absolute frequency) of gap-configuration G_a with its theoretical prediction, $N_{TH}^{\langle 1st \rangle}\left[G_a \mid T\right]$. The prediction was calculated using Eq.(SM-5.3.4) (for a gapped segment C_{K} that exhibits G_a) as:

$$N_{TH}^{\langle 1st \rangle}\left[G_a \mid T\right] = N_T \exp\left\{-\Delta^{Dawg}[\lambda_I, \lambda_I, f_D(.)]|T| - \lambda_D |T|\right\} \\ \times \tilde{\tilde{\mathrm{M}}}_P^{\langle 1st \rangle}\left[\alpha[s_1, s_2, ..., s_{N^X}];\, s_0^{Root}; C_{\mathrm{K}} \mid T\right] \exp\left\{-\lambda_D |T|\right\} \quad \text{. --- Eq.(SM-7.1)}$$

Here N_T is the total number of sites in the root sequences where insertions/deletions

potentially occur. Roughly speaking, $\exp\left\{-\Delta^{Dawg}[\lambda_I, \lambda_I, f_D(.)]|T| - \lambda_D|T|\right\}$ is the probability that the left-flanking gapless column remained undeleted. And $\exp\left\{-\lambda_D|T|\right\}$ is the conditional probability that the right-flanking gapless column remained undeleted, given the gapped segment and the left-flanking gapless column. In this study, we simply set $N_T = 100{,}000 \times 1{,}000 = 10^8$, which is the total number of bases in the root ancestral sequences in each input set. We used only those gap-configurations each of which is expected to occur 5 or more times in each dataset.

We compared the absolute frequencies predicted by Eq.(SM-7.1) against their actual frequencies in each simulated dataset by performing the correlation and linear regression analyses between their square roots. We did so based on the following rationale. The count of each gap-configuration in each simulated dataset is expected to roughly follow a Poison distribution, in which the standard error of the count of events is the square root of its mean. Therefore, the square root of the simulated count is expected to have a standard error that is roughly uniform independently of the gap-configuration. This uniformity of the standard error is a major assumption underlying the correlation and linear regression analyses.

Before the analyses in this and the next sections, we pre-processed the simulated MSAs so that MSAs with a same homology structure [39] will be represented identically. See Methods of [38] for details.

SM-8. Correlation analysis to validate predicted relative probabilities among parsimonious local indel histories

To examine whether or not our formula for the relative probability, Eq.(SM-5.3.6a), works well with each of the simulated sets, 1A, 1B, 3P, 3M and 3F, we first calculated Eq.(SM-5.3.6a) for all alternative parsimonious local indel histories of all "parsimonious" instances of gapped segments that do not reach either MSA end. Then, we distributed the histories enumerated for each input set into 20 non-overlapping bins of 5% width that jointly span the open interval, $(0,1)$, of the theoretical relative probability. (The histories with the relative probability = 1 were excluded from the analyses because they could cause the performance to be unfairly overrated.) In each bin, we counted all instances of alternative histories considered, and we also counted actual instances of "correct" histories, whose ancestral gap states matched the true ones. Then, we compared the simulated proportion (*i.e.*, relative frequency) of "correct" histories in each bin with the theoretically predicted probability that the history is "correct," $\bar{P}_C^{Th}(bin)$. The $\bar{P}_C^{Th}(bin)$ for each bin was calculated

by averaging the relative probabilities given by Eq.(SM-5.3.6a) over all instances of the considered alternative histories in the bin. We performed the correlation and linear regression analyses, using the actual relative frequency in each simulated dataset as the independent variable (X), and using the predicted probability as the dependent variable (Y). For the analyses, we used averages weighted by the reciprocal of the variance of the predicted probability, *i.e.*, $\{weight\} = \dfrac{|bin|}{\bar{P}_C^{Th}(bin)\left(1-\bar{P}_C^{Th}(bin)\right)}$, where $|bin|$ is the total number of instances of considered alternative histories in the bin.

Similar correlation and linear regression analyses were conducted also on the most likely (ML) parsimonious local indel histories alone, as well as on the least likely (LL) parsimonious histories alone.

SM-9. Accuracy of HMM of Kim and Shinha applied to case (iv) local PWAs

Here, we specifically examine the model of Kim and Sinha [36] in the light of our *ab initio* theoretical formulation. Their model is a generalized HMM, and calculates the probability of a PWA between the ancestral and descendant sequences along a branch as a product of block-wise probabilities. In their HMM, a block is either a column of a PAS, a run of gaps in the ancestor aligned with a run of residues in the descendant, or a run of gaps in the descendant aligned with a run of residues in the ancestor. Each PWA is actually a part of a MSA of given sequences at the external nodes and one of alternative sets of sequences at internal nodes. Ancestral gaps aligned with descendant gaps are removed before evaluating the probability of a PWA. Because their purpose is to find a most likely indel history and a resulting set of consistent ancestral sequence states at internal nodes, they are not interested in an indel event that begins and/or ends in the middle of a block (as in Figure S2, panels b and c). Thus, they only consider those events that insert/delete the entire blocks in single steps.

We now calculate the probabilities of the local PWAs that were considered in the cases (i)-(iv) in Section R2 (Figures S1), via the model of [36]. And we compare the results to those via our theoretical formulation under Dawg's parameters (Eqs.(R1.7-9)). In the following, the probabilities via Kim and Sinha will be calculated according to Eq.(2) and Figure 1C of their paper [36], and the probabilities via our formulation will be calculated according to the prescriptions in section SM-2 (and in Appendix A1 of Part II). We set $t_F - t_I = |b|$ in the following calculations. (Here $|b|$ denotes the length of branch b). Via the model of [36], the PWA probability in case (i) is calculated as:

$$P_{KS}\left[case\ (i)\right] = (1-p_I)^2\,(1-p_D)^2 \ , \qquad \text{--- Eq.(SM-9.1)}$$

where p_I and p_D are the "transition probabilities of the 'Insertion' and 'Begin deletion'

states,” respectively. The “reference” PWA probability calculated via our formulation (Eqs.(R1.2,3)) is:

$$P_{ref}\left[case\,(i)\right] = \exp\left(-\Delta^{Dawg}|b| - 2(\lambda_I + \lambda_D)|b|\right) \times\left[1 + \mu_{P(2)}[case\,(i)] + \sum_{n=3}^{+\infty}\mu_{P(n)}[case\,(i)]\right]. \quad \text{--- Eq.(SM-9.2)}$$

Here, Δ^{Dawg} is the abbreviation of the “universal” factor for the indel exit rate (*i.e.*, $\Delta^{Dawg}[\lambda_I, \lambda_D, f_D(.)]$ in Eq.(R1.9)). And $\mu_{P(2)}[case\,(i)]$ is concretely expressed in Eq.(SM-2.2a). Now, assuming that $(\lambda_I + \lambda_D)|b|$ is sufficiently small, we expand the expression in the square brackets into the power series in $\lambda_I|b|$ and $\lambda_D|b|$, which will collectively be denoted as $\lambda|b|$ when considering the order of magnitude. From Eqs.(SM-2.2a,b’), we get $\mu_{P(2)}[case\,(i)] = \sum_{l=1}^{L^{CO}}\lambda_I f_I(l)\lambda_D f_D(l)|b|^2/2 + O\left((\lambda|b|)^3\right)$. Moreover, the expansion of $\mu_{P(n)}\left[\gamma_K;\left(\alpha(s^A, s^D), [t_I, t_F]\right) \mid (s^A, t_I)\right]$ generally starts with $O\left((\lambda|b|)^n\right)$ terms. Thus, we have:

$$P_{ref}\left[case\,(i)\right] = \exp\left(-\Delta^{Dawg}|b| - 2(\lambda_I + \lambda_D)|b|\right) \times\left[1 + \lambda_I\lambda_D|b|^2\left(\sum_{l=1}^{L^{CO}} f_I(l) f_D(l)/2\right) + O\left((\lambda|b|)^3\right)\right]. \quad \text{--- Eq.(SM-9.2')}$$

This and Eq.(SM-9.1) will provide the baseline when examining the probabilities via the HMM of Kim and Sinha in other cases.

In case (ii), the PWA probability under the HMM of Kim and Sinha is:

$$P_{KS}\left[case\,(ii); \Delta L^A\right] = (1-p_I)^3(1-p_D)^2\, p_D\, \mathrm{Pr}_D(\Delta L^A). \quad \text{--- Eq.(SM-9.3)}$$

Here $\mathrm{Pr}_D(l)$ is the “probability distribution on the deletion length (l),” which is assumed as shared among different branches. To facilitate the comparison, we consider the ratio of the probability in case (ii) to that in case (i), which yields:

$$P_{KS}\left[case\,(ii); \Delta L^A\right] \Big/ P_{KS}\left[case\,(i)\right] = (1-p_I)\, p_D\, \mathrm{Pr}_D(\Delta L^A). \quad \text{--- Eq.(SM-9.4)}$$

Meanwhile, the probability via our formulation is:

$$P_{ref}\left[case\,(ii); \Delta L^A\right] = \exp\left(-\Delta^{Dawg}|b| - (\lambda_I + \lambda_D)(2 + \Delta L^A)|b|\right) \times\left[\sum_{n=1}^{+\infty}\mu_{P(n)}[case\,(ii); \Delta L^A]\right]. \quad \text{--- Eq.(SM-9.5)}$$

Here, $\mu_{P(1)}[case\,(ii); \Delta L^A]$ is given by Eq.(SM-2.4’), and $\mu_{P(2)}[case\,(ii); \Delta L^A]$ is given by Eq.(SM-2.5a) supplemented with Eqs.(SM-2.5b,c,d’,e’). The ratio of Eq.(SM-9.5) to Eq.(SM-9.2) is:

$$P_{ref}\left[case\,(ii);\,\Delta L^A\right]/P_{ref}\left[case\,(i)\right]$$
$$= e^{-(\lambda_I+\lambda_D)\Delta L^A|b|}\left[\sum_{n=1}^{+\infty}\mu_{P(n)}[case\,(ii);\,\Delta L^A]\right]\left[1+\sum_{n=2}^{+\infty}\mu_{P(n)}[case\,(i)]\right]^{-1} \quad . \text{--- Eq.(SM-9.6)}$$

Expanding this expression into the power series in $\lambda|b|$, we have:

$$P_{ref}\left[case\,(ii);\,\Delta L^A\right]/P_{ref}\left[case\,(i)\right]$$
$$= \lambda_D|b|f_D(\Delta L^A)+\tfrac{1}{2}\lambda_D(\lambda_I+\lambda_D)|b|^2 G_D(\Delta L^A)+O\left((\lambda|b|)^3\right). \quad \text{--- Eq.(SM-9.6'a)}$$

Here $G_D(\Delta L^A)$ is defined as:

$$G_D(\Delta L^A) \equiv -\Delta L^A f_D(\Delta L^A)+\frac{\lambda_D}{\lambda_I+\lambda_D}\sum_{l=1}^{\Delta L^A-1}(\Delta L^A-l+1)f_D(l)f_D(\Delta L^A-l)$$
$$+\frac{\lambda_I}{\lambda_I+\lambda_D}(\Delta L^A+1)\sum_{l=1}^{\min\{L_I^{CO},L_D^{CO}-\Delta L^A\}} f_I(l)f_D(\Delta L^A+l) \quad . \quad \text{--- Eq.(SM-9.6'b)}$$

(Figure 7 of [43] shows the ratio $G_D(\Delta L^A)/f_D(\Delta L^A)$ as a function of ΔL^A.)

Similarly, via the HMM of [36], the ratio of the PWA probability in case (iii) to that in case (i) is expressed as:

$$P_{KS}\left[case\,(iii);\,\Delta L^D\right]/P_{KS}\left[case\,(i)\right]=\frac{p_I}{1-p_I}\,\mathrm{Pr}_I(\Delta L^D). \quad \text{--- Eq.(SM-9.7)}$$

Here $\mathrm{Pr}_I(l)$ is the "probability distribution on the insertion length (l)," which also is assumed as shared among different branches. The ratio via our formulation is obtained by the power-series expansion in $\lambda|b|$ of Eq.(A1.1.1') and Eq.(A1.1.2a) supplemented with Eqs.(A1.1.2b,c,d',e') (all in Appendix of [43]). The result is:

$$P_{ref}\left[case\,(iii);\,\Delta L^D\right]/P_{ref}\left[case\,(i)\right]=\left[\sum_{n=1}^{+\infty}\tilde{\mu}_P^{(n)}[case\,(iii);\,\Delta L^D]\right]\left[1+\sum_{n=2}^{+\infty}\tilde{\mu}_P^{(n)}[case\,(i)]\right]^{-1}$$
$$=\lambda_I|b|f_I(\Delta L^D)+\tfrac{1}{2}\lambda_I(\lambda_I+\lambda_D)|b|^2 G_I(\Delta L^D)+O\left((\lambda|b|)^3\right) \quad .$$

--- Eq.(SM-9.8a)

Here $G_I(\Delta L^D)$ is defined as:

$$G_I(\Delta L^D) \equiv -\Delta L^D f_I(\Delta L^D)+\frac{\lambda_I}{\lambda_I+\lambda_D}\sum_{l=1}^{\Delta L^D-1}(\Delta L^D-l+1)f_I(\Delta L^D-l)f_I(l)$$
$$+\frac{\lambda_D}{\lambda_I+\lambda_D}(\Delta L^D+1)\sum_{l=1}^{\min\{L_D^{CO},L_I^{CO}-\Delta L^D\}} f_I(\Delta L^D+l)f_D(l) \quad . \quad \text{--- Eq.(SM-9.8b)}$$

(Thanks to the symmetry between the probabilities under the time reversal, Figure 7 of [43] also gives the ratio $G_I(\Delta L^D)/f_I(\Delta L^D)$ as a function of ΔL^D, when calculated under the same setting.)

Now we compare the results under Kim and Sinha's HMM (Eq.(SM-9.4) and Eq.(SM-9.7)) with the corresponding results obtained via our formulation (Eq.(SM-9.6'a) and

Eq.(SM-9.8a)). In the method of [36], the substitutions $p_I = c_I |b|$ and $p_D = c_D |b|$ are made first. Then c_I and c_D are estimated from the total frequencies of insertions and deletions, respectively, along the external branches observed from the input MSA. Similarly, $\Pr_I(\Delta L^A)$ and $\Pr_D(\Delta L^A)$ are estimated from the observed length histograms for insertions and deletions, respectively. Let $\{b\}_{PE}$ be the set of branches used for parameter estimations. Then, using the summations of the "reference" results, Eq.(SM-9.6'a) and Eq.(SM-9.8a), both over $\{b\}_{PE}$, we expect to have:

$$\frac{E[c_D]}{\lambda_D} = 1 + \frac{\lambda_I + \lambda_D}{2} \langle |b| \rangle_{|b|} \sum_{\Delta L^A = 1}^{L_D^{CO}} G_D(\Delta L^A) + O\left((\lambda |b|)^2\right), \quad \text{--- Eq.(SM-9.9a)}$$

$$\frac{E[c_I]}{\lambda_I} = 1 + \frac{\lambda_I + \lambda_D}{2} \langle |b| \rangle_{|b|} \sum_{\Delta L^D = 1}^{L_I^{CO}} G_I(\Delta L^D) + O\left((\lambda |b|)^2\right), \quad \text{--- Eq.(SM-9.9b)}$$

$$\frac{E[c_D]}{\lambda_D} E\left[\Pr_D(\Delta L^A)\right] = f_D(\Delta L^A) + \frac{\lambda_I + \lambda_D}{2} \langle |b| \rangle_{|b|} G_D(\Delta L^A) + O\left((\lambda |b|)^2\right),$$

--- Eq.(SM-9.9c)

$$\frac{E[c_I]}{\lambda_I} E\left[\Pr_I(\Delta L^D)\right] = f_I(\Delta L^D) + \frac{\lambda_I + \lambda_D}{2} \langle |b| \rangle_{|b|} G_I(\Delta L^D) + O\left((\lambda |b|)^2\right).$$

--- Eq.(SM-9.9d)

Here $E[X]$ denotes the expected value of the estimated parameter X, which is the average of estimated X over all indel processes under the given set of conditions (the tree and model parameters). And we also used the notation, $\langle X(b) \rangle_{|b|} \equiv \left[\sum_{b \in \{b\}_{PE}} |b| X(b)\right] \Big/ \sum_{b \in \{b\}_{PE}} |b|$.

[NOTE: The actual values of c_I and c_D estimated by the method of [36] may be slightly smaller than Eqs.(SM-9.9b,c), because the denominator in their method is the total number of MSA columns, instead of the average numbers of possible indel positions in ancestral sequences.] Usually, $\frac{1}{2}(\lambda_I + \lambda_D)\langle |b| \rangle_{|b|}$ is quite small, at most $O(10^{-1})$ and typically $O(10^{-2})$. Thus, as long as the actual parameters, λ_I, λ_D, $f_I(\Delta L^D)$, and $f_D(\Delta L^A)$, do not considerably vary across branches, and provided that the MSA is sufficiently long and accurate, the estimated values of c_I and c_D, respectively, should approximate λ_I and λ_D fairly well. Also, under the same situation, the estimated values of $\Pr_I(l)$ and $\Pr_D(l)$, respectively, should approximate $f_I(l)$ and $f_D(l)$ fairly well, as long as the ratios $G_I(l)/f_I(l)$ and $G_D(l)/f_D(l)$ are sufficiently less than $\left[\frac{1}{2}(\lambda_I + \lambda_D)\langle |b| \rangle_{|b|}\right]^{-1}$. However, it does not actually matter so much whether or not the estimated Kim-Sinha parameters (c_I, c_D,

$\Pr_I(l)$ and $\Pr_D(l)$) approximate Dawg's indel parameters (λ_I, λ_D, $f_I(l)$ and $f_D(l)$) fairly well. What actually matters is how accurately Eq.(SM-9.4) and Eq.(SM-9.7) approximate Eq.(SM-9.6'a) and Eq.(SM-9.8a), respectively, using the estimated parameters. In an extreme case where all branches have the same branch length, the approximation should be nearly perfect. This is because, in this case, $|b| \approx \langle |b| \rangle_{|b|}$ for all branches, and thus because we can use Eqs.(SM-9.9a-d) without any significant modifications to estimate the probabilities (not involving case (iv)). [It should be noted here that Eq.(SM-9.4) and Eq.(SM-9.7), respectively, contain extra multiplication factors, $(1-p_I)$ and $(1-p_I)^{-1}$, compared to the corresponding Eq.(SM-9.6'a) and Eq.(SM-9.8a). However, these factors should remain close to 1, because $p_I = c_I |b|$ should normally be at most $O(10^{-1})$.] In contrast, the approximations by Eq.(SM-9.4) and Eq.(SM-9.7) could considerably deteriorate, *e.g.*, when $\left|(\lambda_I + \lambda_D)\left(|b| - \langle |b| \rangle_{|b|}\right)\right|$ times the ratios, $G_D(\Delta L^A)/f_D(\Delta L^A)$ and $G_I(\Delta L^D)/f_I(\Delta L^D)$, respectively, become comparable to or greater than 1 (unity). Because $G_D(\Delta L^A)$ and $G_I(\Delta L^D)$ are mostly contributed from next-parsimonious local histories containing overlapping indels, we can interpret the result as follows. "Overlapping indels start to make Kim and Sinha's method poorly approximate the (case (ii) and (iii)) local PWA probabilities when the involved gap is long and the branch lengths show a large variation." Let's assume that there is a good reason to believe that the Dawg indel parameters (λ_I, λ_D, $f_I(l)$ and $f_D(l)$) are shared among all branches. Then, one way to mitigate the aforementioned effects of overlapping indels may be to set:

$$c_D\left(|b|\right)\Pr_D\left(l,|b|\right) = \lambda_D f_D(l) + \lambda_D \frac{\lambda_I + \lambda_D}{2}|b| G_D(l), \quad \text{--- Eq.(SM-9.10a)}$$

$$c_I\left(|b|\right)\Pr_I\left(l,|b|\right) = \lambda_I f_I(l) + \lambda_I \frac{\lambda_I + \lambda_D}{2}|b| G_I(l), \quad \text{--- Eq.(SM-9.10b)}$$

and to fit λ_I, λ_D, $f_I(l)$ and $f_D(l)$ according to these equations supplemented with Eqs.(SM-9.6'b,8b). Now, as indicated by Figure 7 of [43], under the power-law indel length distributions, the ratios $G_D(\Delta L^A)/f_D(\Delta L^A)$ and $G_I(\Delta L^D)/f_I(\Delta L^D)$ are less than 4 in absolute value when the gap is 300 residues long or shorter. Therefore, the 2nd-order terms will begin to substantially influence the results when $\left|\frac{1}{2}(\lambda_I + \lambda_D)\left(|b| - \langle |b| \rangle_{|b|}\right)\right|$ is larger than, say, 0.1. Such a situation will be quite rare in practical sequence analyses. Even if we encounter such a rare case, then local histories with more than 2 indels will begin to account

for a substantial fraction of the probability. Considering this way, we expect that the method of Kim and Sinha [36] will pretty well approximate the probabilities of local PWAs belonging to cases (ii) and (iii) as long as the branch lengths are reasonable for phylogenetic analyses.

Finally, we consider case (iv). Indel histories giving rise to the local sequence states in this category are shown, *e.g.*, in Figure S3 (in this paper), and panel A of Figure 6 of [49]. In such a situation, an aligner will reconstruct a PWA that is like one of the two PWAs in Figure S1, panel d (if the reconstruction is correct). And Kim-Sinha's method assigns a probability according to the reconstructed PWA. Whether it is like the left one or the right one in Figure S1d, the assigned probability is the same, and its ratio to the probability of case (i) is:

$$P_{KS}\left[case\,(iv);\,\Delta L^A,\Delta L^D\right]\big/P_{KS}\left[case\,(i)\right]=p_I\,\Pr_I(\Delta L^D)\,p_D\,\Pr_D(\Delta L^A)\,. \quad \text{--- Eq.(SM-9.11)}$$

Via our formulation, how to calculate the probability in this case was briefly described near the bottom of section SM-2. (And it is detailed in Appendix A1.2 of [43].) In this case, each parsimonious history consists of two indels, and each next-parsimonious history consists of three indels. Because there are as many as 24 types of next-parsimonious histories, here we only consider the parsimonious histories. Then, the lowest-order contribution of the multiplication factor, $\mu_{P(2)}[case\,(iv);\,\Delta L^A,\Delta L^D]$, is given by Eq.(A1.2.1a), supplemented with Eqs.(A1.2.1b,c,d,e',f',g'), all in [43]. [NOTE: In [43], $\mu_{P(2)}[case\,(iv);\,\Delta L^A,\Delta L^D]$ is denoted as $\tilde{\mu}_P^{(2)}[case\,(iv)]$.] Expanding each term into a power series in $\lambda|b|$, we get the following expression for the ratio:

$$\begin{aligned}
&P_{ref}\left[case\,(iv);\,\Delta L^A,\Delta L^D\right]\big/P_{ref}\left[case\,(i)\right]\\
&=e^{-(\lambda_I+\lambda_D)\Delta L^A|b|}\left[\sum_{n=2}^{+\infty}\mu_{P(n)}[case\,(iv);\,\Delta L^A,\Delta L^D]\right]\left[1+\sum_{n=2}^{+\infty}\mu_{P(n)}[case\,(i)]\right]^{-1}\\
&=\lambda_D\,\lambda_I\,|b|^2\left[\tfrac{1}{2}f_D(\Delta L^A)\,f_I(\Delta L^D)+\left\{\sum_{l=0}^{\min\{L_I^{CO}-\Delta L^D,\,L_D^{CO}-\Delta L^A\}}f_I(\Delta L^D+l)\,f_D(\Delta L^A+l)\right\}\right]\\
&\qquad+O\left((\lambda|b|)^3\right)\,.
\end{aligned}$$

--- Eq.(SM-9.12)

In case (iv), as opposed to in cases (ii) and (iii), the PWA probabilities via the HMM of [36] differ considerably from that via our formulation *even when* $\langle|b|\rangle_{|b|}<<1$ and $|b|<<1$. Under these conditions, $p_I\,\Pr_I(\Delta L^D)$ and $p_D\,\Pr_D(\Delta L^A)$ quite accurately approximate $\lambda_I\,|b|\,f_I(\Delta L^D)$ and $\lambda_D\,|b|\,f_D(\Delta L^A)$, respectively (see Eqs.(SM-9.9c,d)). Hence, the $O\left((\lambda|b|)^3\right)$ terms in

Eq.(SM-9.12) can be neglected. Thus, we have:

$$\frac{P_{ref}\left[case\,(iv);\,\Delta L^A,\Delta L^D\right]\big/P_{ref}\left[case\,(i)\right]}{P_{KS}\left[case\,(iv);\,\Delta L^A,\Delta L^D\right]\big/P_{KS}\left[case\,(i)\right]} \approx \frac{3}{2} + \sum_{l=1}^{\min\{L_I^{CO}-\Delta L^D,\, L_D^{CO}-\Delta L^A\}} \frac{f_I(\Delta L^D + l)\, f_D(\Delta L^A + l)}{f_D(\Delta L^A)\, f_I(\Delta L^D)} \;.$$

--- Eq.(SM-9.13)

Table 3 shows this ratio for representative cases. The second term on the right hand side of Eq.(SM-9.13) is the effect of overlapping indels (as in Figure S3, panels d and e). When $\Delta L^A = \Delta L^D = 1$, this term is expected to be quite small; for example, it is about 0.167 if $f_I(l) = f_D(l) \propto l^{-1.6}$. And it gets more and more influential when ΔL^A and/or ΔL^D gets larger, and it substantially exceeds 1 (unity) in some cases (Table 3). Actually, a similar effect was incorporated in the HMM of Knudsen and Miyamoto [50]. Their HMM could only accommodate geometric indel length distributions. Consequently, the relevant term was independent of ΔL^A and ΔL^D. Coming back to Eq.(SM-9.13), the first term on the right hand side, 3/2, differs from 1 (unity) because the HMM of [36] does not fully take account of the non-overlapping indel histories (*e.g.*, panels a-c of Figure S3), either. [NOTE: More precisely, their HMM takes account of contributions only from either panels a and b or panels a and c, depending on the local HMM it deals with, but not from all of panels a, b and c.] This error is actually shared by most of the standard, or nearly standard, HMMs and transducers used thus far as probabilistic models of indels (such as those cited in Background of Part I). Taking these results into consideration, a possible major improvement on the model of Kim and Sinha [36] would be achieved through modifying the HMM structure, so that the probability of an insertion and an immediately adjacent deletion (or that of the opposite configuration) will be given by Eq.(SM-9.13) or its extension that includes the terms of higher-orders in $\lambda|b|$.

Additional References

63. Press WH, Teukolsky SA, Vetterling WT, Flannery BP. Numerical Recipes in C: The Art of Scientific Computing, 2nd edition. Cambridge (UK): Cambridge University Press; 1992.

64. Farris JS. Phylogenetic analysis under Dollo's law. Syst Zool. 1977;26:77-88.

Supplementary tables

Table S1. Various "threshold gap lengths" for local PWAs of cases (ii) and (iii)

$X = (\lambda_I + \lambda_D)(t_F - t_I)$ [a]	$(\Delta L)_{0.5}^{(NP)}$ [b]	$(\Delta L)_{0.5}^{(1)}$ [c]	$(\Delta L)_{0.5}^{(2)}$ [c]	$(\Delta L)_{0.5}^{(5)}$ [c]
0.01 indels/site	128	160	> 300	> 300
0.04 indels/site	31	41	99	272
0.1 indels/site	12	17	42	119
0.2 indels/site	6	8	22	66
Approximate relation [d]	$Y \approx 1.2/X$	$Y \approx 1.6/X$	$Y \sim 4/X$	$Y \sim 11/X$ (?)

NOTE: See section M1 of Methods for details on the parameter settings. Because of the symmetry under the time reversal when $\lambda_I = \lambda_D$, the identical results apply to the local PWAs in both case (ii) and case (iii). This table is adapted from Table 1 of [43].

[a] The expected number of indels per site.

[b] The number of ancestral sites in between the PASs, *i.e.*, ΔL^A or ΔL^D, at which the total next-parsimonious contribution is 1/2 (=0.5) of the total parsimonious contribution.

[c] $(\Delta L)_{0.5}^{(N_{ID})}$ is the value of ΔL^A or ΔL^D at which the total contribution from local histories involving up to (and including) N_{ID} indels each account for 1/2 (=0.5) of the "exact" multiplication factor for the local PWA.

[d] A rough (inversely proportional) relationship between each threshold gap length (Y) and the expected number of indels per site (X).

Table S2. Goodness of power-law for finite-time transition probabilities of case (iii) local PWAs

$\lambda_I : \lambda_D$ [a]	$(\lambda_I + \lambda_D)(t_F - t_I)$ [b]	Correlation coefficient	Exponent [c] (Std. Err.(γ))	Coefficient [d] (Std.Err.($\log A$))
1 : 1	0.001 indels/site	-0.9999998	1.5994 (5.8×10^{-5})	0.000223 (9.6×10^{-5})
	0.01 indels/site	-0.9999997	1.5998 (7.6×10^{-5})	0.00222 (0.00013)
	0.04 indels/site	-0.9999946	1.5993 (0.00030)	0.00881 (0.00050)
	0.1 indels/site	-0.999968	1.5981 (0.00074)	0.0217 (0.0012)
	0.2 indels/site	-0.99989	1.5955 (0.0014)	0.0421 (0.0023)
1 : 3	0.001 indels/site	-0.9999998	1.5998 (5.2×10^{-5})	0.000111 (8.6×10^{-5})
	0.01 indels/site	-0.9999994	1.6036 (0.00011)	0.00111 (0.00017)
	0.04 indels/site	-0.999990	1.6143 (0.00042)	0.00442 (0.00069)
	0.1 indels/site	-0.99994	1.6340 (0.0010)	0.01090 (0.0016)
	0.2 indels/site	-0.99981	1.6623 (0.0019)	0.0213 (0.0029)
3 : 1	0.001 indels/site	-0.9999998	1.5990 (6.3×10^{-5})	0.000334 (0.00010)
	0.01 indels/site	-0.9999997	1.5960 (7.3×10^{-5})	0.00333 (0.00012)
	0.04 indels/site	-0.999995	1.5846 (0.00028)	0.01317 (0.00047)
	0.1 indels/site	-0.99997	1.5636 (0.00065)	0.0323 (0.0011)
	0.2 indels/site	-0.99991	1.5329 (0.0012)	0.0625 (0.0021)

NOTE on Table S2 (previous page): This table shows the results of the correlation and regression analyses. The independent variable (X) is $\log(\Delta L)$, where ΔL is the local PWA size. The dependent variable (Y) is $\log\left(\tilde{\mu}_P^{\langle N_{ID}=200\rangle}\left[case\,(iii);\,\Delta L;\,[t_I,t]\right]\right)$, where

$\tilde{\mu}_P^{\langle N_{ID}=200\rangle}\left[case\,(iii);\,\Delta L;\,[t_I,t]\right]$ is the "exact" multiplication factor associated with a local PWA of case (iii). See subsection SM-3.1 of Supplementary methods for more details. See section M1 of Methods for the parameter setting. The results apply also to case (ii) local PWAs with due modifications.

[a] λ_I is the total insertion rate per site per unit time. λ_D is the total deletion rate per site per unit time.

[b] The expected number of indels per site.

[c] The power-law exponent, *i.e.*, γ of the approximate power-law, $\tilde{\mu}_P^{\langle N_{ID}=200\rangle}[\Delta L] \approx A(\Delta L)^{-\gamma}$.

[d] The power-law coefficient, *i.e.*, A of the approximate power-law, $\tilde{\mu}_P^{\langle N_{ID}=200\rangle}[\Delta L] \approx A(\Delta L)^{-\gamma}$.

Table S3. Correlation and regression analyses on absolute frequencies of local MSA homology structures

Dataset	Homology structures analyzed	Number of homology structures	Correlation coefficient	Slope (Std. Err.)	Y-intercept (Std. Err.)
1A	All	3,396	0.99958	0.99064 (0.00050)	-0.229 (0.014)
	Rare invisible indels [a]	3,390	0.99958	0.99065 (0.00050)	-0.228 (0.014)
1B	All	11,157	0.99752	0.96467 (0.00064)	-0.706 (0.016)
	Rare invisible indels [a]	9,831	0.99917	0.97221 (0.00040)	-0.532 (0.011)

NOTE: The independent variable (X) is the square root of the actual absolute frequency of each homology structure in each simulated dataset. The dependent variable (Y) is the square root of the absolute frequency predicted by Eq.(SM-7.1) in Supplementary methods. We analyzed homology structures that are predicted to occur 5 times or more in each of the MSA sets 1A and 1B. See section M2 of Methods for details on the simulations. This table is a modified version of Table 1 of [48].

[a] Homology structures of local MSAs each of which is expected to undergo less than one unobservable indel.

Table S4. Correlation and regression analyses on relative frequencies of correct parsimonious indel histories

Dataset	Parsimonious indel histories used	Number of histories [a]	Correlation coefficient	Slope (Std. Err.)	Y-intercept (Std. Err.)
1A	All	317,400	0.999948	1.0105 (0.0025)	-0.0045 (0.0011)
	Most likely (ML)	119,676	0.999793	0.9987 (0.0049)	0.0058 (0.0038)
	Least likely (LL)	119,856	0.999683	1.0105 (0.0090)	-0.0097 (0.0024)
1B	All	7,252,601	0.999967	1.0132 (0.0019)	-0.00153 (0.00023)
	Most likely (ML)	917,499	0.999848	0.99911 (0.00411)	0.0125 (0.0032)
	Least likely (LL)	925,036	0.999441	0.99905 (0.0118)	-0.0136 (0.0017)
3P	All	152,051	0.9994	0.9839 (0.0081)	0.0055 (0.0035)
	Most likely (ML)	70,041	0.9966	1.021 (0.020)	-0.023 (0.013)
	Least likely (LL)	70,041	0.9991	1.036 (0.016)	-0.0032 (0.0046)
3M	All	808,462	0.999988	0.9986 (0.0011)	-0.00016 (0.00018)
	Most likely (ML)	181,501	0.9994	1.017 (0.008)	-0.017 (0.007)
	Least likely (LL)	181,496	0.99983	1.034 (0.0067)	-0.00037 (0.00036)
3F	All	2,355,122	0.999990	1.0028 (0.0010)	-0.00013 (0.00004)
3F	Most likely (ML)	140,485	0.99987	1.0051 (0.0037)	-0.0024 (0.0031)
3F	Least likely (LL)	140,478	0.99985	1.0060 (0.0062)	-0.0019 (0.0007)

NOTE on Table S4 (previous page): Here we analyzed all instances of local MSAs in each of which one of 2 or more parsimonious indel histories was "correct." The independent variable (X) is the simulated proportion that the alternative parsimonious indel histories in each bin actually yielded the corresponding homology structures of the local MSAs ("simulated relative frequency"). The dependent variable (Y) is the average of the predicted relative probabilities of the histories in each bin ("predicted relative frequency"). Note that weighted analyses were conducted. For details on the simulations and the correlation/regression analysis, see section M2 of Methods and SM-8 of Supplementary methods, respectively. The upper half of this table was adapted from Table 2 of [48].

[a] The number of instances of alternative parsimonious histories of the specified type (All/ML/LL).

Supplementary figures (with legends)

a Case (i)

A		L	R
D		L	R

b Case (ii)

A		L	1	2	3	R
D		L	-	-	-	R

c Case (iii)

A		L	-	-	R
D		L	υ_1	υ_2	R

d Case (iv)

A		L	1	2	3	-	-	R
D		L	-	-	-	υ^D_1	υ^D_2	R

or

(

A		L	-	-	1	2	3	R
D		L	υ^D_1	υ^D_2	-	-	-	R

)

Figure S1. Four types of local gap configurations in PWA between ancestral and descendant sequences.

a Case (i). **b** Case (ii) with $\Delta L^A = 3$. **c** Case (iii) with $\Delta L^D = 2$. **d** Case (iv) with $\Delta L^A = 3$ and $\Delta L^D = 2$.

In each PWA, each site (a cell) is assigned an ancestry. In the leftmost column of each PWA, the boldface italic 'A' and 'D' stand for an ancestor (s^A) and a descendant (s^D), respectively. The boxes shaded in magenta and cyan represent unpreserved ancestral sites and inserted descendant sites, respectively. In panel **d**, the PWA on the right (in parentheses) is equivalent to the PWA on the left, as far as the homology structure alone is concerned. This figure was adapted from Figure 2 of [43].

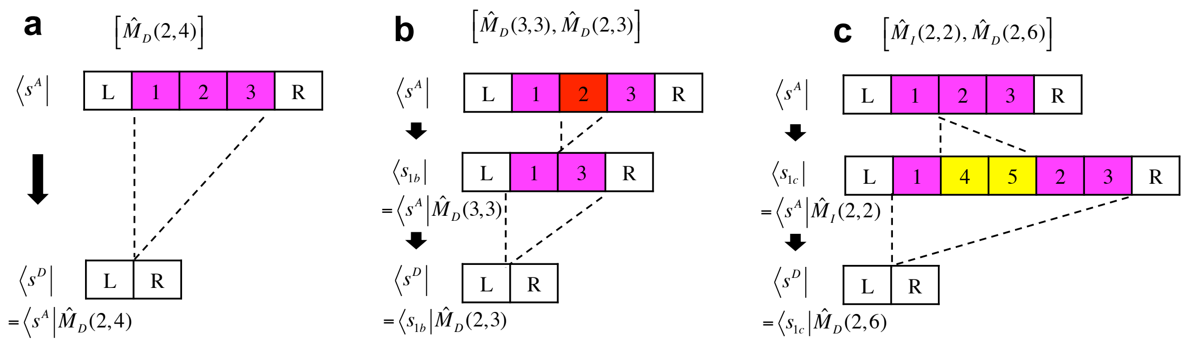

Figure S2. Parsimonious and next-parsimonious indel histories yielding case (ii) local PWA.

a The parsimonious history, consisting of a single deletion. **b** and **c** Examples of non-parsimonious indel histories. That in **b** consists of two consecutive deletions. That in **c** consists of an insertion and a subsequent deletion.

Each of these indel histories yields the local PWA in Figure S1, panel **b**. The boxes shaded in magenta and red represent ancestral sites to be deleted. The yellow-shaded boxes represent inserted sites to be deleted.

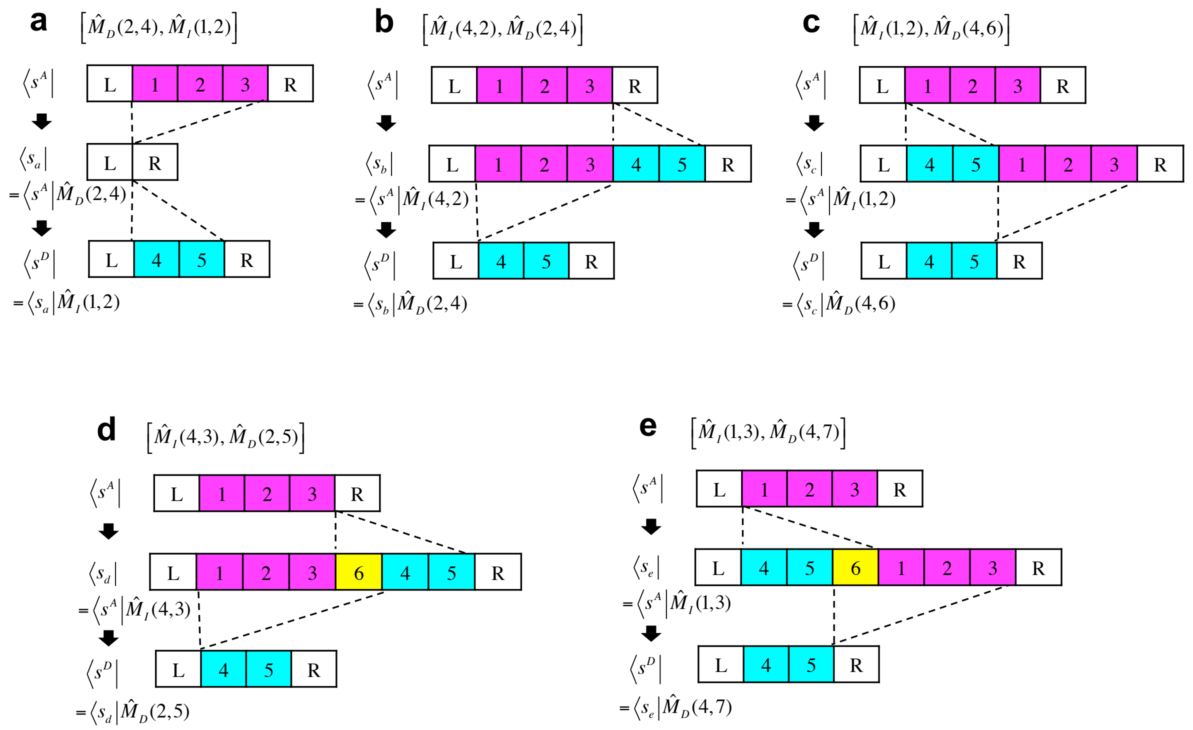

Figure S3. Parsimonious indel histories yielding case (iv) local PWA.

Each panel shows a parsimonoius indel history that results in the local PWA in Figure S1, panel **d**. Panels **a**, **b** and **c** exhaust the histories with non-overlapping indels. Panels **d** and **e** exemplify the histories with overlapping indels. The boxes shaded in magenta, cyan and yellow, respectively, represent ancestral sites to be deleted, descendant sites that were inserted, and inserted sites to be deleted.

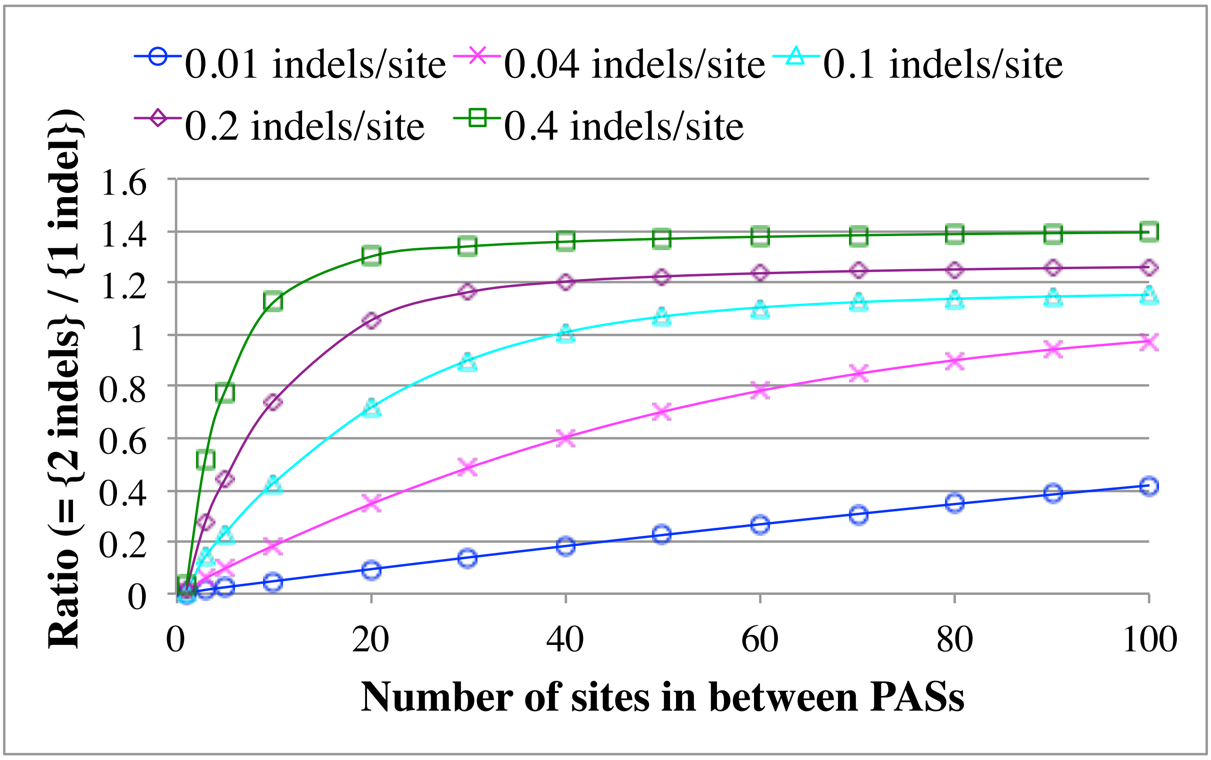

Figure S4. Goodness of first approximation for case (ii) (and (iii)) local PWAs.
The graph shows the ratio of the total next-parsimonious contribution (by 2-indel histories) to the total parsimonious contribution (by 1-indel histories) for case (ii) or (iii) local PWAs, as the function of the number of sites (ΔL^A in case (ii) and ΔL^D in case (iii), abscissa) and the distance ($(\lambda_I + \lambda_D)(t_F - t_I)$ indels/site, different curves). See section M1 of Methods for the parameter setting. This figure is a modified version of Figure 3A of [43].

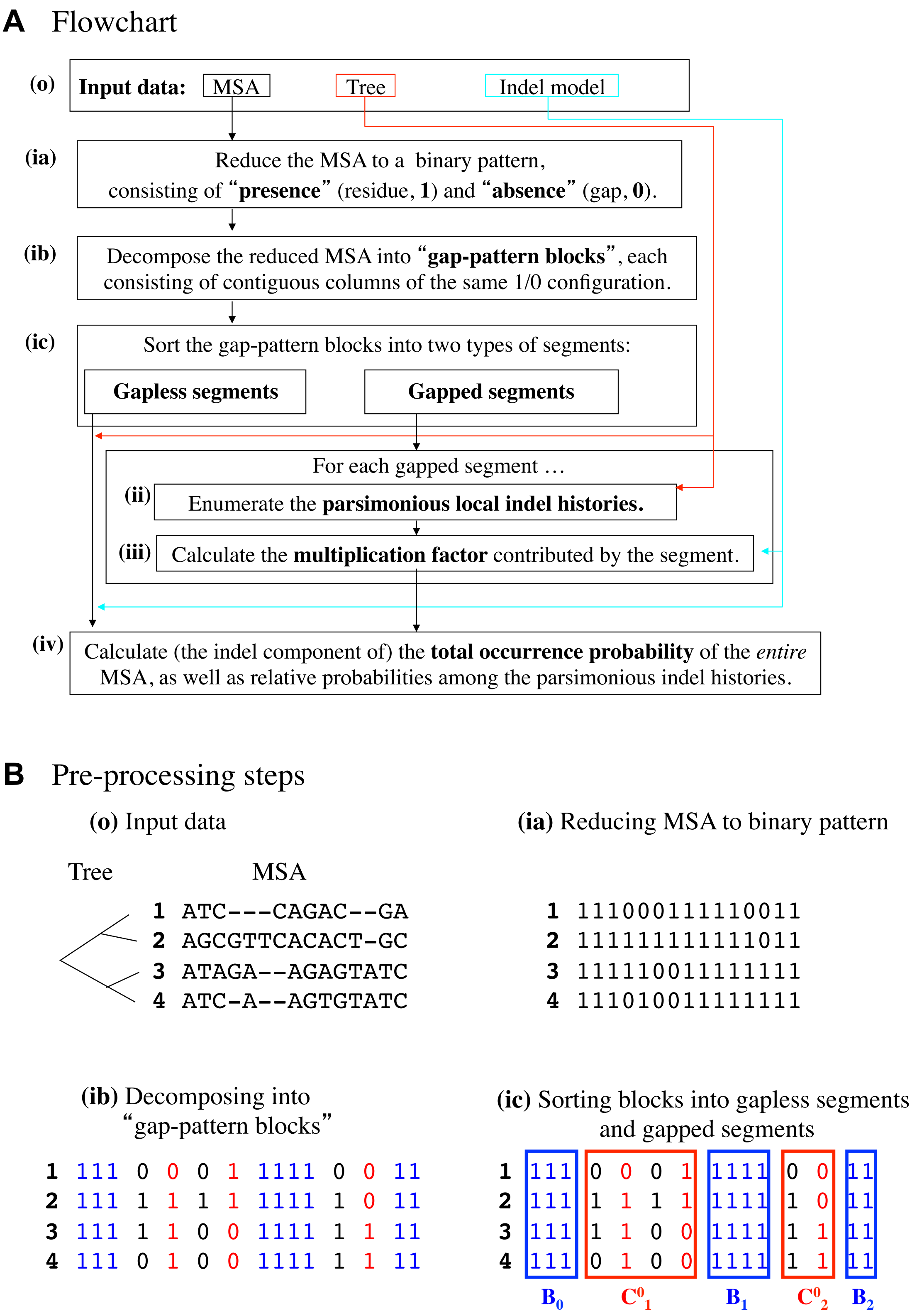

Figure S5. Overall workflow of our algorithm to calculate first-approximate *ab initio* MSA probability.

The entire algorithm consists of steps (ia), (ib), (ic), (ii) and (iii), processing the input (o) into the final output at step (iv). **A** The flowchart. **B** The schematic illustration of the

pre-processing steps (ia-ic). The input data [**(o)**] consists mainly of a MSA (of DNA sequences here) and a phylogenetic tree of the aligned sequences (labeled with boldface numbers). An evolutionary model via indels is assumed to be given but is omitted here. Step **(ia)** reduces the input MSA to a binary 1/0 pattern, in which 1 and 0 represent the "presence" (of a residue) and the "absence" (*i.e.*, a gap), respectively. Step **(ib)** decomposes the binary pattern into "gap-pattern block"s, or "block"s for short, each of which consists of contiguous columns of a given 1/0 pattern. Here each block is represented as a rectangular array of neighboring cells with a particular color. Step **(ic)** sorts the blocks into gapless segments and gapped segments. Each gapless segment is represented as contiguous blue cells enclosed by a blue rectangle labeled B_k (with $k = 0,1,2$). And each gapped segment is represented as contiguous cells enclosed by a red rectangle labeled C_K^0 (with $K = 1,2$). See section SM-5.1 for more details. [NOTE: The set of all gapped segments, $\{C_K^0\}_{K=1,2,...}$, is a subset of $\{C_K\}_{K=1,2,...,K_{max}}$, which is the set of all regions that can accommodate local indel histories along the tree.] This figure was adapted from Figure 1 of [48].

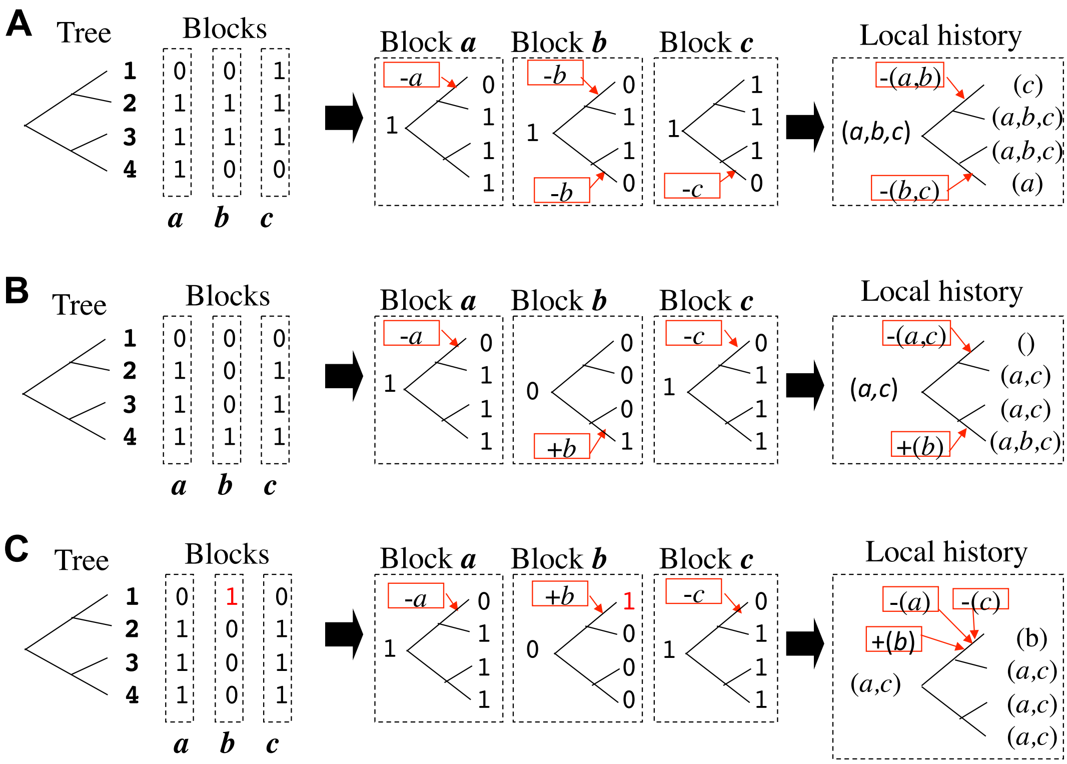

Figure S6. Merging indel events in effectively contiguous gap-pattern blocks.

In each panel, a gapped segment consisting of contiguous gap-pattern blocks ("block"s), and a phylogenetic tree of aligned sequences (left) is given. Then, the Dollo parsimonious history for each block is first inferred (middle). Second, the indel histories in the effectively contiguous blocks are merged if they are of the same type and occur along the same branch (right). As in Figure S5 B, a "1" and a "0" represent the presence state (*i.e.*, a residue) and the absence state (*i.e.*, a gap), respectively. Note that each column under the "Blocks" (left) represents a gap-pattern block, and not necessarily a single column, in the MSA. In the indel histories in the middle step, "+x" and "-y" represent the insertion of block "x" and the deletion of block "y", respectively. In the local indel histories in the final step (on the right), blocks in the same parentheses after the "+" or the "-" sign, respectively, are inserted or deleted simultaneously. **A** Merging indel events in literally contiguous blocks. **B** Merging indel events in two blocks separated by a (run of) block(s) in which no downstream nodes with the "presence" state interrupt the merger. **C** In this case, the deletions of block *a* and block *c*, both along the exterior branch leading to sequence 1, cannot be merged because they are interrupted by the downstream node with the "presence" state (the red "1") in block *b*. This figure was adapted from Figure 2 of [48].

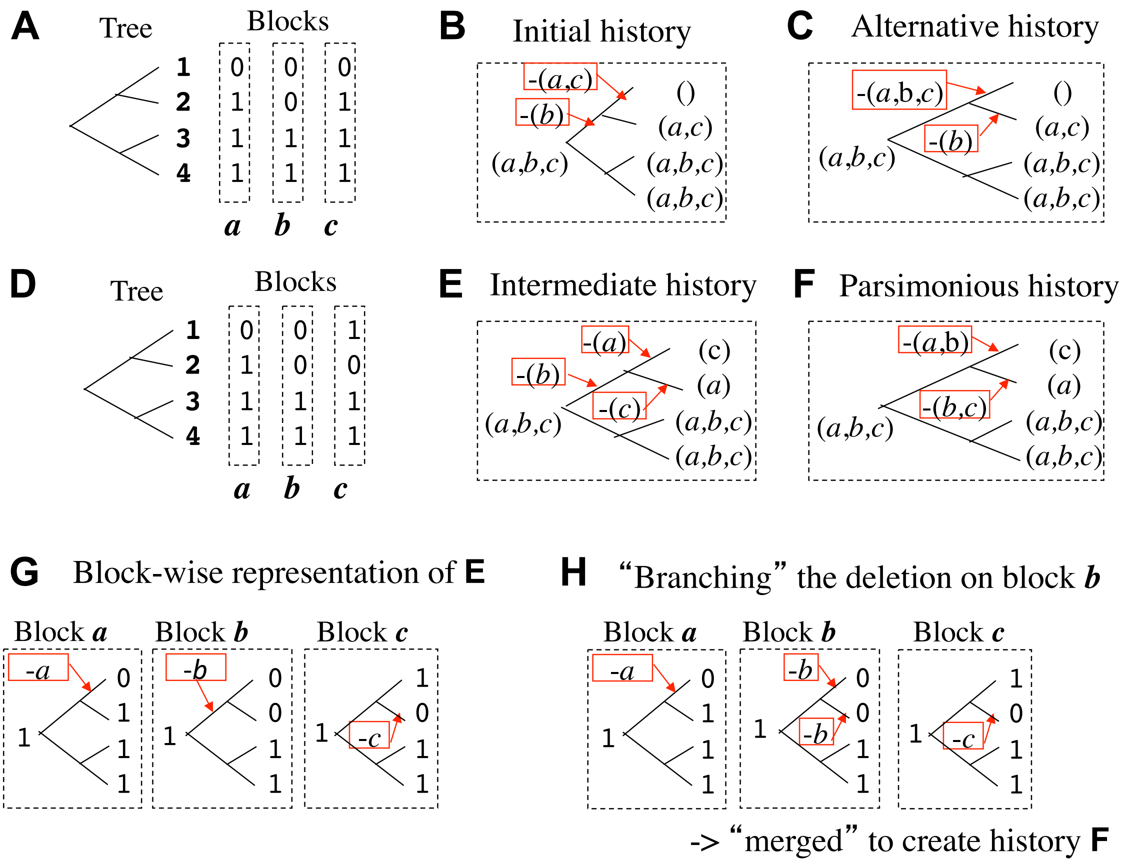

Figure S7. Looking for parsimonious local indel histories.

For the gap-configuration (under the "Blocks") and the tree shown in **A**, the initial step infers the history in **B**, but there is actually another parsimonious history **(C)**. For the gap-configuration and the tree shown in **D**, using the history in **E** as an "intermediate" point always reachable from the initial history, we can find the actual parsimonious history shown in **F**. Panels **G** and **H** exemplify a "branch-and-merge" operation performed on the situation in **D**. **G** Looking closely at the indel history in **E**, we see that a deletion of a subsequence in block *b* occurs along the branch of the common ancestor of sequences 1 and 2. With this history as a starting point, in the "branching" step **(H)**, the deletion is re-interpreted as deletions along the child branches. Finally, merging the resulting deletions with the effectively contiguous deletion(s) gives the local indel history in **F** in this example. This figure was adapted from Figure 3 of [48].

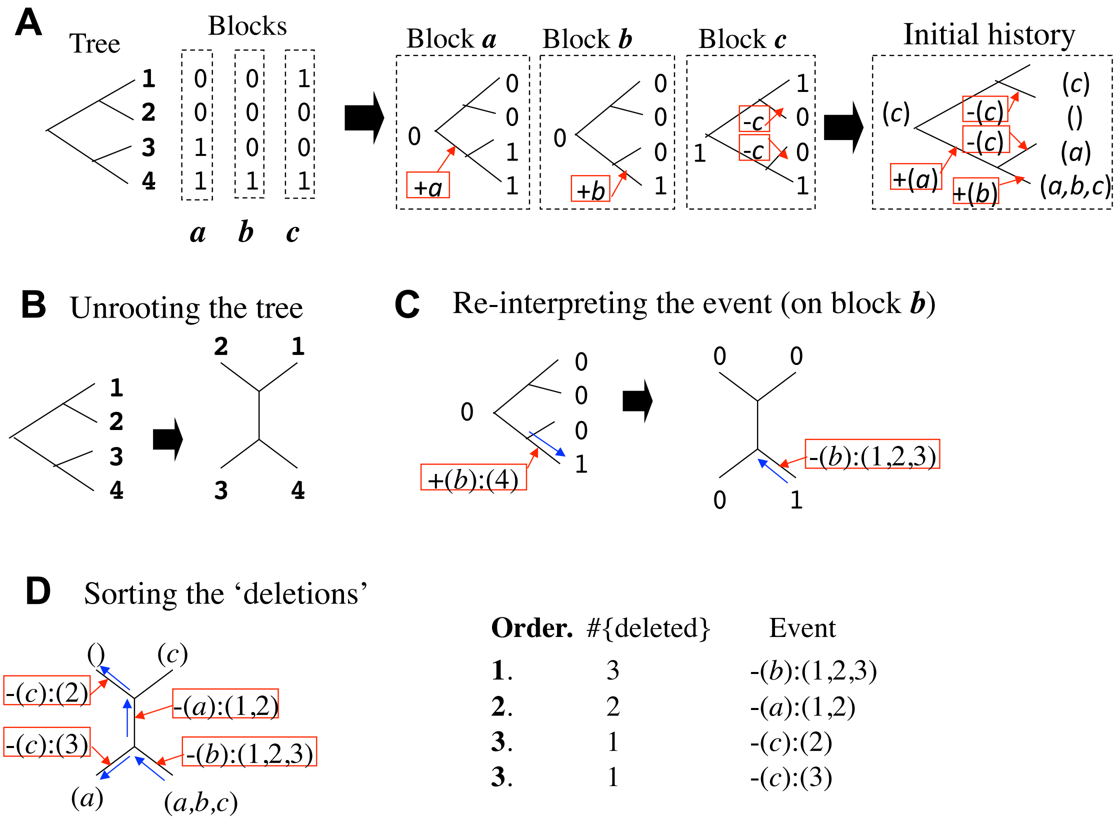

Order.	#{deleted}	Event
1.	3	-(b):(1,2,3)
2.	2	-(a):(1,2)
3.	1	-(c):(2)
3.	1	-(c):(3)

Figure S8. 4. Sorting indel events that will undergo "branch-and-merge" processes. **A** The initial local indel history (right), given a gapped segment (under the "Blocks") and a sequence tree (left). **B** If the input tree is rooted, it gets unrooted. **C** Then, an insertion event (as in block *b* in this example) can be re-interpreted as a 'deletion' event by reversing the (virtual) time direction (represented by a blue arrow). Here, "+(*b*):(4)" denotes the insertion of block *b* into sequence 4, and "-(b):(1,2,3)" denotes the 'deletion' of block *b* from (the 'last common ancestor' of) sequences 1, 2, and 3. Similarly, "+(a):(3,4)" in the original history will also be re-interpreted as "-(a):(1,2)." **D** In this way, we can re-interpret all the indel events as 'deletions' (left), and sort them in descending order of the number of sequences undergoing the 'deletion' (right). This figure was adapted from Figure 4 of [48].

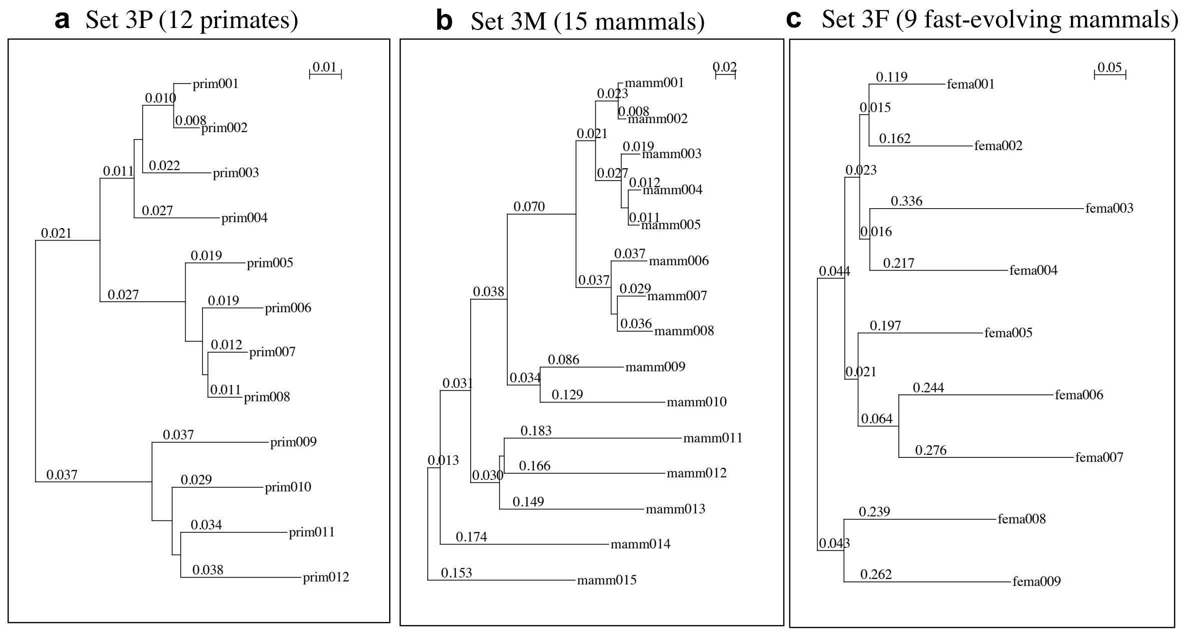

Figure S9. Phylogenetic trees used for simulated DNA sequence evolution.

a The tree of 12 primates, used for Set 3P. **b** The tree of 15 mammals, used for Set 3M. **c** The tree of 9 fast-evolving mammals, used for Set 3F. This figure was adapted from Figure 2 of [38]. See [38] for more details on these trees. See section M2 of Methods for the settings for the simulations.

a Set 3P

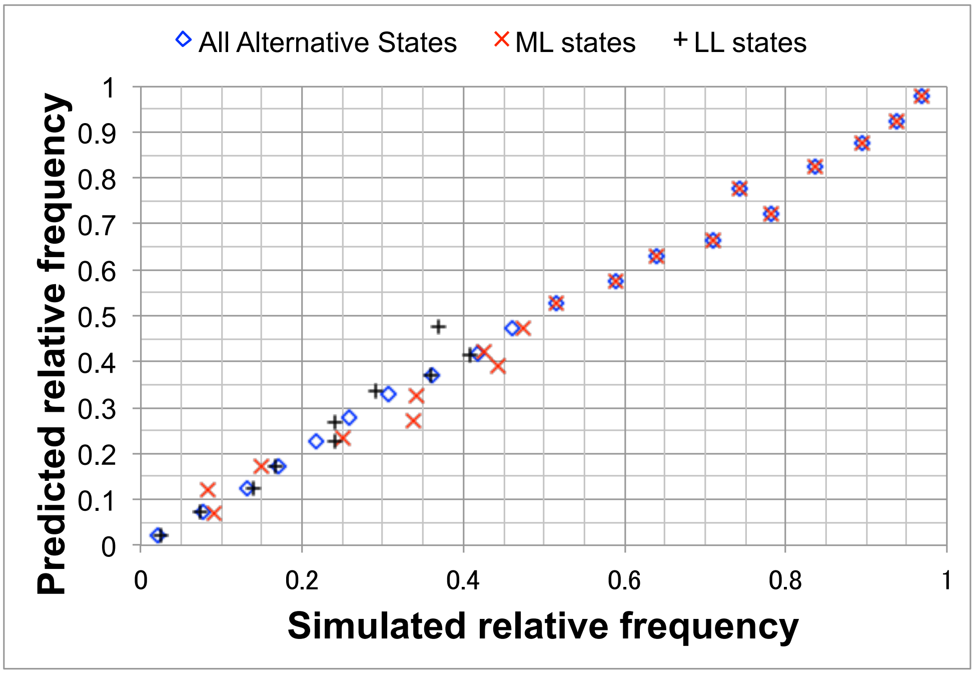

b Set 3F

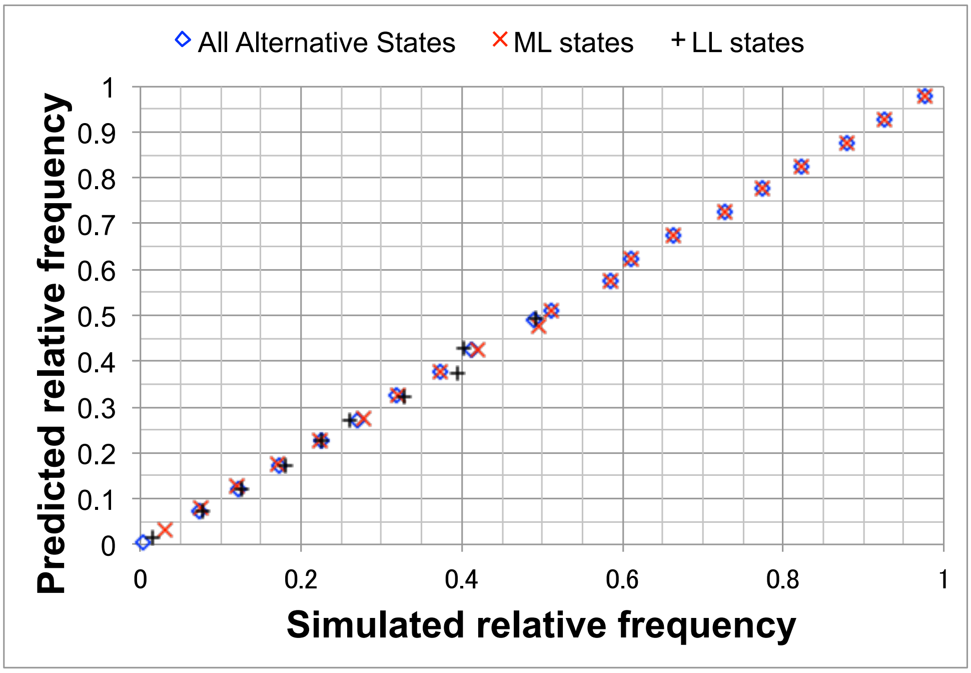

Figure S10. Simulation analyses on relative frequencies among local indel histories, using Set 3P (a) and Set 3F (b).

The same notation and convention apply as those for Figure 7.

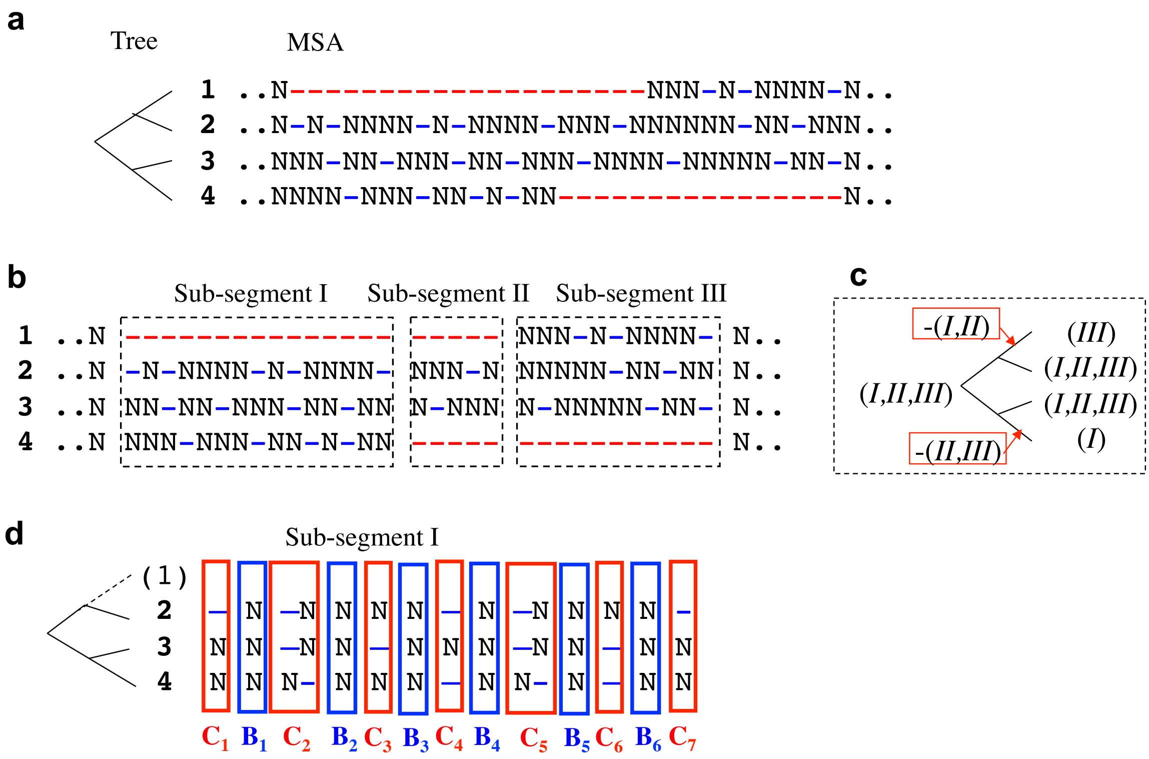

Figure S11. Problem with long gaps and its solution by hierarchical partitioning. **a** A gapped segment with long gaps (red) often contains lots of short gaps (blue). This makes the simple partitioning less effective. **b** A coarse-grained partitioning according only to the configuration of long gaps, chopping a gapped segment into a number of sub-segments (I, II and III in this example). **c** A broad indel history resulting in the configuration of long gaps. **d** Fine-grained partitioning of the Sub-segment I in panel **b** according to the configurations of short gaps. This measure decomposes the 'big' problem into a set of sub-problems. The 'big' problem needs to consider potentially numerous indel histories, whereas each sub-problem needs only to consider a few candidate histories. Ignoring the sequence containing the long gap enables this measure. The ignored sequence is indicated by the parenthesized sequence ID and the dotted branch leading to it. Each column in this figure should be regarded as a gap-pattern block rather than a single site. This figure was adapted from Figure 30 of [48].
